# Foraminifera as a model of eukaryotic genome dynamism

**DOI:** 10.1128/mbio.03379-23

**Published:** 2024-02-08

**Authors:** Caitlin Timmons, Kristine Le, H. B. Rappaport, Elinor G. Sterner, Xyrus X. Maurer-Alcalá, Susan T. Goldstein, Laura A. Katz

**Affiliations:** 1 Department of Biological Sciences, Smith College, Northampton, Massachusetts, USA; 2 Division of Invertebrate Zoology, American Museum of Natural History, New York, New York, USA; 3 Department of Geology, University of Georgia, Georgia, Athens, USA; 4 University of Massachusetts Amherst, Program in Organismic and Evolutionary Biology, Amherst, Massachusetts, USA; University of California, Los Angeles, Los Angeles, California, USA

**Keywords:** genome evolution, eukaryotic life cycles, polyploidy, nuclear architecture, endoreplication, amoebae, protists, microbial eukaryote

## Abstract

In contrast to the canonical view that genomes cycle only between haploid and diploid states, many eukaryotes have dynamic genomes that change content throughout an individual’s life cycle. However, the few detailed studies of microeukaryotic life cycles render our understanding of eukaryotic genome dynamism incomplete. Foraminifera (Rhizaria) are an ecologically important, yet understudied, clade of microbial eukaryotes with complex life cycles that include changes in ploidy and genome organization. Here, we apply fluorescence microscopy and image analysis techniques to over 2,800 nuclei in 110 cells to characterize the life cycle of *Allogromia laticollaris* strain Cold Spring Harbor (CSH), one of few cultivable foraminifera species. We show that haploidy and diploidy are brief moments in the *A. laticollaris* life cycle and that *A. laticollaris* nuclei endoreplicate up to 12,000 times the haploid genome size. We find that *A. laticollaris* reorganizes a highly endoreplicated nucleus into thousands of haploid genomes through a non-canonical mechanism called *Zerfall*, in which the nuclear envelope degrades and extrudes chromatin into the cytoplasm. Based on these findings, along with changes in nuclear architecture across the life cycle, we believe that *A. laticollaris* uses spatio-temporal mechanisms to delineate germline and somatic DNA within a single nucleus. The analyses here extend our understanding of the genome dynamics across the eukaryotic tree of life.

**IMPORTANCE:**

In traditional depictions of eukaryotes (i.e., cells with nuclei), life cycles alternate only between haploid and diploid phases, overlooking studies of diverse microeukaryotic lineages (e.g., amoebae, ciliates, and flagellates) that show dramatic variation in DNA content throughout their life cycles. Endoreplication of genomes enables cells to grow to large sizes and perhaps to also respond to changes in their environments. Few microeukaryotic life cycles have been studied in detail, which limits our understanding of how eukaryotes regulate and transmit their DNA across generations. Here, we use microscopy to study the life cycle of *Allogromia laticollaris* strain CSH, an early-diverging lineage within the Foraminifera (an ancient clade of predominantly marine amoebae). We show that DNA content changes significantly throughout their life cycle and further describe an unusual process called *Zerfall*, by which this species reorganizes a large nucleus with up to 12,000 genome copies into hundreds of small gametic nuclei, each with a single haploid genome. Our results are consistent with the idea that all eukaryotes demarcate germline DNA to pass on to offspring amidst more flexible somatic DNA and extend the known diversity of eukaryotic life cycles.

## INTRODUCTION

Our understanding of eukaryotic genome organization draws heavily from studies of “model” plants and animals, while the microeukaryotic lineages (a.k.a. protists) that comprise the bulk of eukaryotic biodiversity are understudied (1, 2). Genome dynamics, including fluctuations in DNA content within eukaryotic life cycles, remain underappreciated despite documentation across diverse eukaryotic lineages (reviewed in 3, 4). For example, in animals such as lampreys, copepods, and nematodes, elimination of germline-limited DNA during development (i.e., chromatin diminution) leads to substantial differences in DNA content between germline and somatic cells (5). Ciliates, a group of microbial eukaryotes with distinct somatic and germline genomes, undergo a similar process, eliminating ~30%–95% of their germline DNA during the development of a new somatic genome (6, 7). Meanwhile, *Amoeba proteus* undergoes a form of cyclic polyploidy which consists of extensive endoreplication immediately following karyokinesis and then chromatin extrusion to eliminate the replicated DNA before the subsequent nuclear cycle (3, 8).

Foraminifera (Rhizaria) are a diverse (~10,000–15,000 species) and ancient (~600 million years ago) clade of predominantly marine amoebae that build “shells” (test or theca) (9, 10). As some foraminifera tests readily fossilize, this clade has been critical in paleoecological studies aiming to reconstruct environmental conditions and estimate biological productivity across different timescales (e.g., references 11–13). Despite their utility as paleoindicators, little is known about the diversity of genomes within this clade, as most studies of foraminiferal genome dynamics were conducted prior to modern molecular and microscopy techniques (e.g., references 14–18). Nevertheless, these older studies suggest that foraminifera have dynamic and flexible genomes (reviewed in reference 10).

A “typical” foraminifera life cycle alternates between haploid and diploid generations, but there are many deviations from this classical model. In the typical life cycle, a haploid individual has a single nucleus that increases in size and DNA content and ultimately produces gametes, which fuse to form diploid offspring. During the diploid phase, individual cells become multinucleated and eventually undergo meiosis, releasing numerous haploid daughter cells (10; reviewed in reference 19). Fluctuations in ploidy and genome content during life cycle transitions are common in foraminifera (20–22), evidenced by endoreplication via whole-genome duplication or copy number variation in haploid, and perhaps also diploid, individuals (15, 23). Endoreplication and nuclear division also lead to heterokaryosis—the coexistence of genetically distinct nuclei within a single individual—in some members of the foraminiferal clade Globothalamea (10, 22, 24, 25). Prior to the generation of gametes, at least some foraminifera undergo dramatic genome rearrangements through a process termed *Zerfall* (i.e., “decay” [10, 14, 21]). During *Zerfall*, the nuclear envelope of the single, haploid, endoreplicated nucleus degrades, releasing all of the genetic material into the cytoplasm where it eventually forms hundreds of gametes (14, 21). Though first described by Føyn in 1936, *Zerfall* is infrequently observed even in detailed life cycle studies (15, 17, 20, 26).

Detailed studies of foraminiferan life cycles remain challenging, as most species are currently uncultivable. This study focuses on the monothalamid *Allogromia*, an early-diverging foraminiferal lineage (27, 28) characterized by an organic single-chambered test and one of the few currently cultivable foraminifera. In the last century, two papers—Arnold (20) and McEnery and Lee (29)—provide in-depth descriptions of *Allogromia’*s life cycle. Arnold (20) is a 70 page treatise based largely on light microscopy, while McEnery and Lee (29) make inferences from observations and microspectrophotometry data. While it is possible that these authors did not use the same strain in their studies, both demonstrated that *Allogromia laticollaris* has a non-canonical life cycle that alternates between uninucleate and multinucleate stages. However, these authors disagree on ploidy levels at some stages and on the placement of critical life cycle transitions such as *Zerfall*, gametogenesis, and meiosis, leaving the overall life cycle unresolved (20, 29). Additional studies on this species, incorporating PCR and fluorescence microscopy, demonstrated that the genome content varies with food source (bacteria alone vs bacteria + mixed eukaryotic algae [30]) and show potential differential amplification of rDNA and beta-tubulin genes across life cycle stages (23).

Here, we characterize the life cycle of *A. laticollaris* strain CSH (Cold Spring Harbor) through brightfield and fluorescence microscopy, making inferences about genome size and ploidy fluctuations through image analysis. We document cell growth, life cycle duration, and morphological transitions through longitudinal observations of 96 isolated cells and populations in culture flasks, finding life cycle synchrony among individuals. We also develop reliable protocols to stain DNA and newly transcribed RNA in *Allogromia*. Applying these techniques to 2,860 nuclei in 110 cells (among ~1,000 observed), we demonstrate that *A. laticollaris* CSH’s nuclear architecture and DNA content varies substantially across life stages. Finally, we present detailed observations on the stages of *Zerfall*, a thus far enigmatic and dramatic genome rearrangement process that exemplifies the inherent plasticity of eukaryotic genomes. Through these observations, we suggest that *Allogromia* uses spatio-temporal mechanisms to distinguish germline and somatic DNA and that this may expand our understanding of eukaryotic genome organization.

## MATERIALS AND METHODS

### Culture acquisition and maintenance

*Allogromia laticollaris* CSH cultures were originally isolated from Cold Spring Harbor, NY, in 1960 (31) and then maintained by a variety of scientists (in order: Sam Bowser and Jeff Travis [Wadsworth Center, Albany New York], Laura Parfrey [Smith College], and Yana Eglit [Dalhousie]) before the cultures returned to Smith College in spring 2021 from Yana Eglit. Cultures were stored in a humid incubator at 24°C on a 12-h/12-h light/dark cycle in ventilated flasks with a 32-ppt solution of Volvic water and Instant Ocean sea salt. We fed cultures *Isochrysis* algae, maintained in F/2 Nutrient Solution (UTEX, Austin, TX) as described in reference 30.

### Brightfield microscopy life cycle observations

Using brightfield microscopy (Olympus CKX53), we aimed to (i) estimate the duration of each *Allogromia laticollaris* CSH life cycle stage (ii), estimate growth rates, and (iii) explore reproductive synchrony among cells. To this end, we tracked 96 individual cells across two “generations,” defined as the time from one reproductive phase to another. We isolated eight reproductive cells from four culture flasks and observed them daily until juveniles emerged (~1 day). We transferred juvenile cells into two 24-well plates such that each row contained six offspring from a single reproductive cell. We maintained plates with the culture conditions described above and imaged (Olympus CKX53) and recorded life stage transitions for each cell daily during reproductive stages and biweekly otherwise. To track “generation 2,” we transferred offspring from two “early emerging” (total life duration, <25 days), four “middle emerging” (total life duration, 25–35 days), and two “late emerging” (total life duration, >35 days) reproductive cells from “generation 1” to two new 24-well plates and then replicated the other details from “generation 1” (see Fig. S1 for experimental setup). We classify cells as “early,” “middle,” or “late” emerging (File S1) based on life cycle stage durations from a previous pilot experiment using the same methods, which tracked individual cells from juvenile to adult. Using the images taken, we measured the cell area and diameter with ImageJ (W. S. Rasband, ImageJ, U.S. National Institutes of Health, Bethesda, MD).

### Fixation and nuclear staining

We hand picked *A. laticollaris* CSH cells from culture flasks by pipetting into sterile 1.5-mL tubes, in which we performed all fixation, permeabilization, and wash steps. After removing excess liquid, we fixed cells for 30 minutes at room temperature in a 200-µL solution of 3.2% glyoxal (Sigma-Aldrich, St. Louis, MO), 4% ethanol, and 1% glacial acetic acid (Sigma-Aldrich), with the final solution at pH 2 (32).

Afterwards, we performed three 5-minute washes of fixed cells in 200 µL of 1× phosphate-buffered saline (PBS). Cells were then permeabilized in 200 µL 100% methanol for 15 minutes at room temperature. After permeabilization, we performed three 5-minute washes in 200 µL 1× PBS. Cells were then incubated in 1.5-mL tubes with 100 µL 0.01 µg/µL Hoechst 33342 (Invitrogen, Carlsbad, CA) solution for 20 minutes in the dark, shaking on a shaker platform set to 125 rpm to ensure that the solution was evenly distributed. We performed another series of three 5-minute washes in 200 µL 1× PBS, removing excess liquid after the last wash before pipetting cells in 10 µL liquid to charged SuperFrost slides (Fisher, Waltham, MA). We mounted cells in a drop of SlowFadeGold (Invitrogen), sealed slides with a cover slip and nail polish, and stored them in the dark at 4°C until imaging. We applied this fixation and nuclear staining protocol to *Saccharomyces cerevisiae* cells, *Homo sapiens* epithelial cells, and *Allium cepa* root tip cells to provide “standard” ratios of nuclear fluorescence to DNA content to make DNA content and genome size inferences in *Allogromia* and demonstrate our protocol’s efficacy on a variety of eukaryotic cells.

### Nascent RNA labeling via click chemistry

We labeled nascent RNA in *Allogromia laticollaris* CSH cells using the Click-iT RNA Alexa Fluor 488 Imaging Kit (Invitrogen). We pipetted cells into small petri dishes in 195 µL seawater, fed cells with 5 µL of previously frozen *Isochrysis* algae, and incubated cells at 24°C overnight. The next day, we incubated cells in 1 mM ethynyl-uridine (EU) solution for 20 minutes and then transferred them to sterile 1.5-mL tubes. We fixed cells in a 200-µL solution composed of 32% paraformaldehyde, RNAlater (Invitrogen), and TRIzol (Invitrogen) in a 12.5:85.5:2 ratio. Following fixation, we washed cells three times for five minutes each using 200 µL of 1× PBS. We permeabilized cells in 200 µL 0.5% Tween-20 solution for 30 minutes, followed by another series of three 5-minute washes in 200 µL 1× PBS. We then incubated *A. laticollaris* cells in 500 μL Click-iT reaction cocktail containing AlexaFluor 488 azide for 30 minutes and washed once with 1 mL Click-iT Rinse Buffer. We washed the cells once more in 200 µL 1× PBS, before staining DNA and preparing slides in the same manner as described in the previous section.

### Fluorescent imaging

We collected fluorescent images using a Leica TCS SP5 laser-scanning confocal microscope (Leica, Mannheim, Germany) with a 63× oil immersion objective. We used a UV laser with an excitation wavelength of 405 nm to collect Hoechst (DNA) signal and brightfield images and an argon laser with an excitation wavelength of 488 nm to collect AlexaFluor 488 azide (RNA) signal. We captured images of cell cross-sections using a resolution of 1,024 × 1,024 px with an acquisition speed of 200 Hz and a line average of 16 scans. We captured cross-sections of individual nuclei in the same manner except with a line average of 32 scans. We collected z-stacks at a resolution of 1,024 × 1,024 px, acquisition speed of 200 Hz, a line average of 6 scans, and a step size of 0.13 µm. Smart gain and offset varied slightly across all images to adjust for variability in fixation quality and Hoechst penetration.

### Image analysis

We measured the volume and fluorescence intensity of each nucleus in z-stacks of *A. laticollaris* CSH, *S. cerevisiae*, *H. sapiens*, and *A. cepa* cells using Nikon NIS-Elements image analysis software. We measured the diameter of each cell in brightfield images using ImageJ software (W. S. Rasband, ImageJ, U.S. National Institutes of Health, Bethesda, MD). We analyzed z-stacks for individual cells using the General Analysis 3 feature of NIS-Elements Advanced Research software (Nikon, Tokyo, Japan). We manually determined the threshold setting for each z-stack to ensure that nuclear volumes were defined accurately before measurement. For each nucleus, we recorded the volume, total fluorescence intensity (measured in F), and mean fluorescence intensity.

We used *S. cerevisiae*, *H. sapiens*, and *A. cepa* nuclei as standards for comparison of *A. laticollaris* CSH measurements. We calculated the average fluorescence intensity of nuclei from each of the three standards and used these measurements to calculate the average ratio of base pairs to fluorescent unit (bp/F) for each species. Using two methods, we estimated the DNA content in each *A. laticollaris* CSH nucleus. Method 1 multiplies the total fluorescence in each *A. laticollaris* CSH nucleus by the *S. cerevisiae* bp/F ratio to estimate the nuclear DNA content. We used this method since the inferred haploid *A. laticollaris* CSH nuclei are closest in size and fluorescence to *S. cerevisiae* nuclei and our calculated *S. cerevisiae* bp/F ratio is most consistent with ratios reported in previous literature for similar staining protocols (33). Method 2 multiplies the total fluorescence in each *A. laticollaris* CSH nucleus by the average bp/F ratio across all standard nuclei to estimate the nuclear DNA content. We used this method since it accounts for our staining protocol’s performance on differently sized nuclei. Method 2 produces a standard deviation approximately 14 times that of method 1. Ultimately, we used method 1 to estimate the *A. laticollaris* CSH haploid genome size.

## RESULTS

### The *Allogromia laticollaris* life cycle progresses through distinct morphological phases

**Fig 1:**
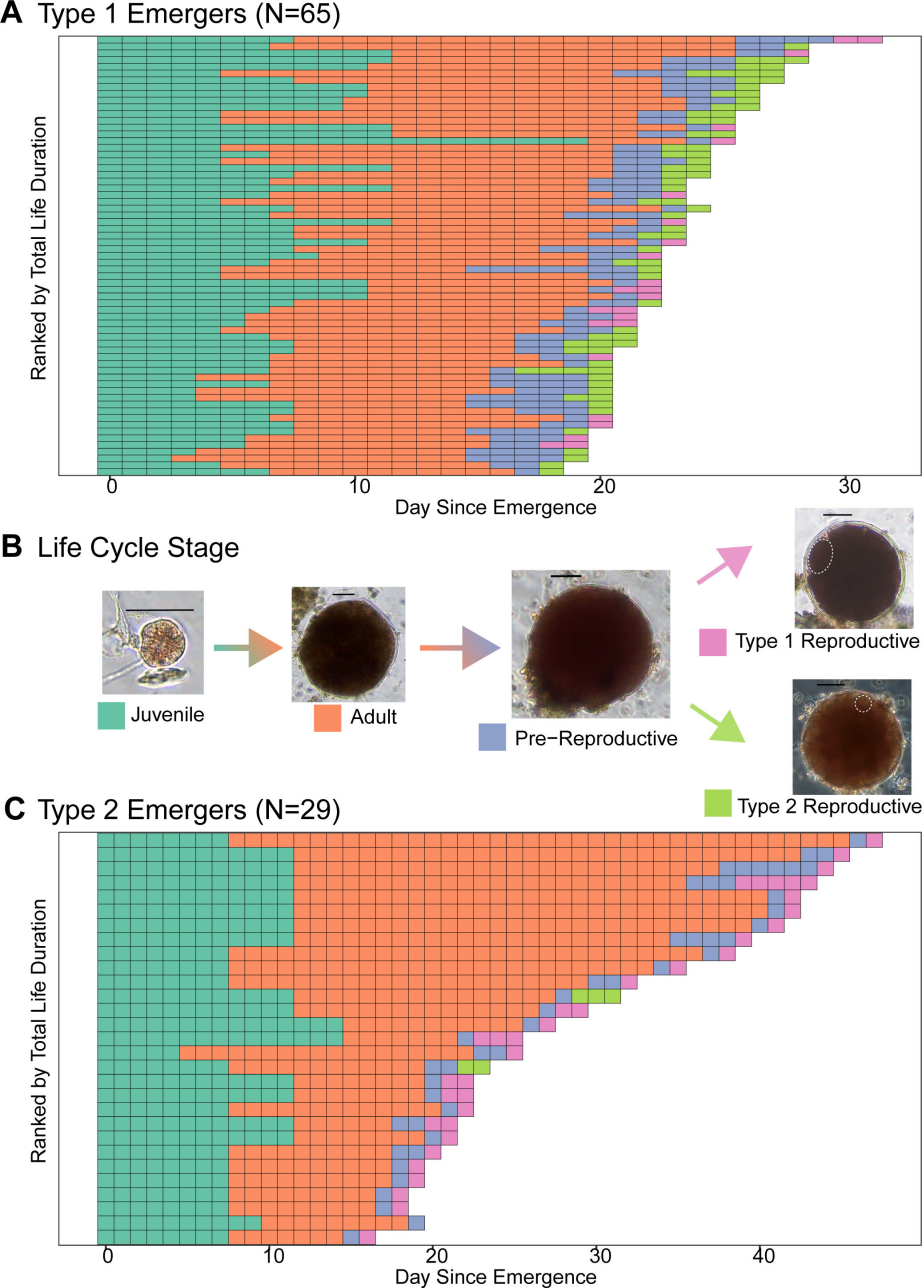
Stacked bar plot of life stage durations separated by the type of reproductive cell emerged from and life cycle characterization based on light microscopy. The life cycle of *A. laticollaris* CSH begins with juveniles that emerge from parental test and grow into adults, which are defined by their size, dark pigmentation, and complex pseudopodia. Adult cells transition into pre-reproductive cells and in the process retract their pseudopods. Pre-reproductive cells develop into either Type 1 or Type 2 reproductive cells that then give rise to juveniles; the outline of a next-generation juvenile is shown as a dashed circle. Each tile represents one cell on a given day after emergence, with tiles colored by life stage. Cells are ranked by their total life duration on the *y*-axis. (**A**) Life stage durations of Type 1 emergers (inferred to be uninucleate; see text). (**C**) Life stage durations of Type 2 emergers (inferred to be multinucleate; see text). (**B**) Example cells at each life cycle stage. Scale bars are 100 µm. Type 1 reproductive cells give rise to fewer, larger juveniles while Type 2 cells give rise to a greater number of smaller juveniles.

We track offspring of individual adults and characterize life cycle stages as juvenile, adult, pre-reproductive, and reproductive (Fig. 1B; Table S1). Juveniles are lightly pigmented with simple pseudopodial networks and transition into adult cells that we define based on their size (100–310 µm in diameter; Table S2; File S1), dark pigmentation, and complex pseudopodial networks (Fig. 1B). Adult cells transition to pre-reproductive and reproductive stages, during which the cell retracts its pseudopods and divides its cytoplasm into membrane-bound juveniles (Fig. 1B; Table S2). We identify two types of reproductive cells: Type 1 and Type 2. This categorization is based on the number and size of juveniles produced by the reproductive cells in comparison to each other; Type 1 reproductive cells (221 µm average diameter) produce fewer (~13), larger juveniles than Type 2 reproductive cells (205 µm average diameter), which produce smaller and more than double (~30) the number of juveniles (Fig. 1B; Table S2). Juveniles that emerge from Type 1 reproductive cells are called Type 1 emergers while those that emerge from Type 2 reproductive cells are named Type 2 emergers. To our surprise, we find that transitions across life stages are relatively synchronous, even among “siblings” raised separately (Fig. 1A and C; see synchronicity section below). Overall, the total life cycle duration of Type 1 emergers is 31 ± 10 days and 25 ± 3 days for Type 2 emergers (Table S2).

We find that individuals can either develop into the same or alternate type of reproductive cell as their parent. Most juvenile cells observed developed into the opposite type of reproductive cell as their parent; 72% (47/65) of Type 1 emergers developed into Type 2 reproductive cells and 93% (27/29) of Type 2 emergers developed into Type 1 reproductive cells (Fig. 1A and C; File S1). *Allogromia laticollaris* CSH cells also appear to undergo synchronous life cycle transitions when cultured in communal environments (i.e., culture flasks). By observing three culture flasks daily for a month, we find that most cells in a flask are in the same life stage at any given time and undergo life stage transitions at similar times (Table S3; File S2). For example, in flask 1, most cells transitioned from large adults to a mixture of juveniles, adults, and pre-reproductive cells after 5 days and then to small juveniles and medium adults after another 8 days. Finally, after 10 more days (23 days total), most cells were once again large adults, pre-reproductive, and reproductive cells (Table S3).

*Allogromia laticollaris* CSH also exhibits alternative life cycle pathways; out of the 96 cells tracked by light microscopy, 3 cells deviated from the life cycle sequence described above (Fig. S2). One cell lived for 2 months without progressing beyond the adult stage (Fig. S2A) and is included in the life stage duration data set shown in Fig. 1. The remaining two cells reproduced via budding and are the offspring of cells included in our light microscopy experiment but are not included in Fig. 1. The first budding cell (Fig. S2B) was the sole “juvenile” from its parent at a size of 200 µm, which is more typical of a large adult than a juvenile. This cell budded 15 days after emergence and gave rise to five juveniles that were each larger than 100 µm in diameter. The other budding cell had normal adult characteristics (see above) prior to budding a single offspring (Fig. S2C).

### Fluorescence microscopy reveals distinct nuclear architectures across life cycle stages

**Fig 2:**
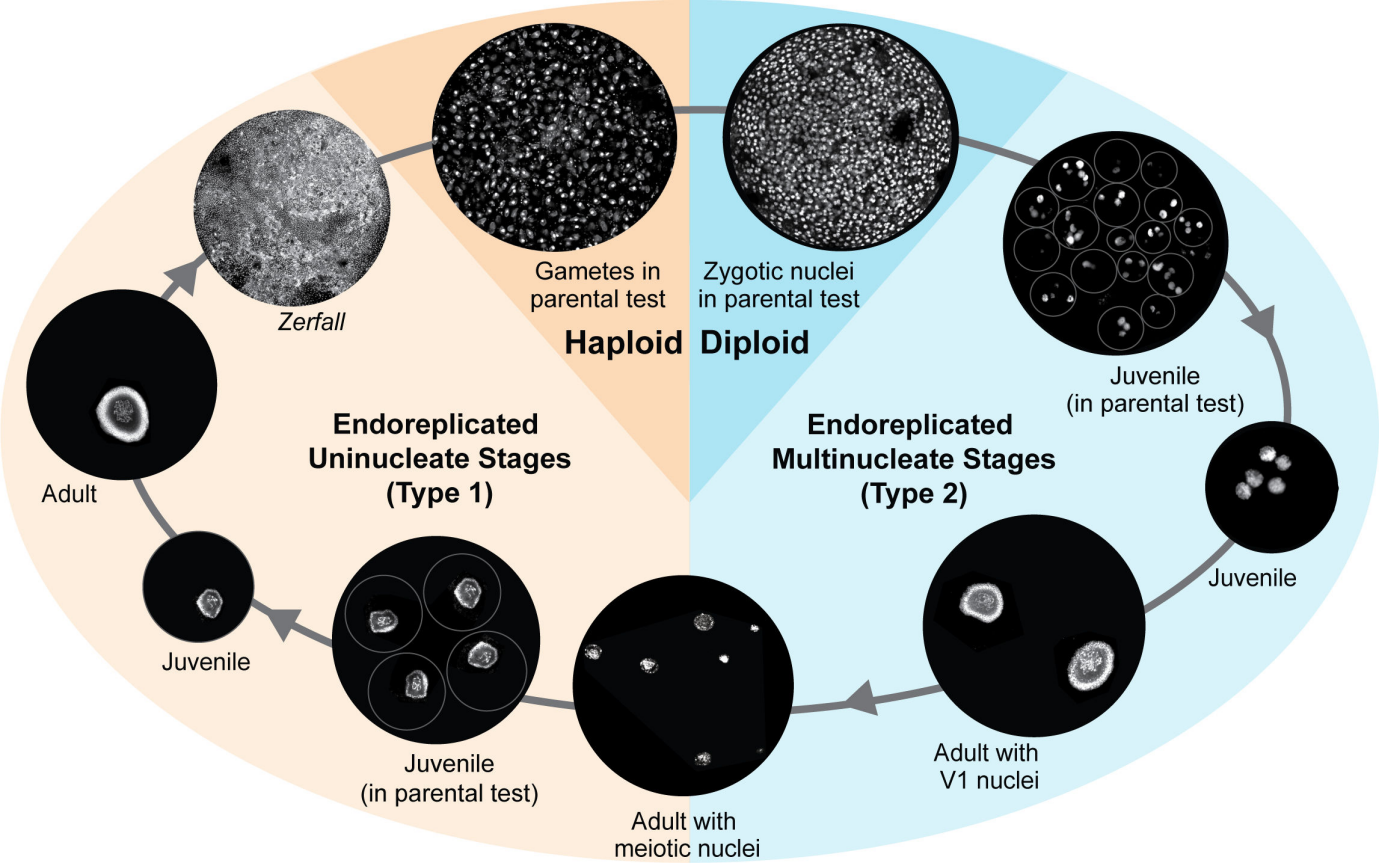
Proposed life cycle for *Allogromia laticollaris* CSH: the *Allogromia* life cycle includes brief haploid and diploid stages, with all other stages containing endoreplicated genomes. Cells are represented by black circles containing images of Hoechst-stained DNA captured by a confocal laser scanning microscope and depicted here in white (see File S4 for complete images). Blue colors are Type 2 cells where the euploidy is 2N (i.e., diploid), while warmer colors are Type 1 cells where the euploidy is N (i.e., haploid). Beginning with the haploid stage, amoeboid gametes fuse within the parent test (i.e., autogamy) to form diploid zygotic nuclei. These zygotic nuclei are compartmentalized to generate multinucleate juveniles, which, upon release from the parent, grow into adults with uniform V1 nuclei (see Table 1). Meiosis occurs within multinucleate adults, with centralized chromosomes forming meiotic bouquets (MB) that are surrounded by a chromatin ring. Meiosis appears asynchronous among nuclei within a cell, since cells in meiosis have heterogeneous nuclear architecture (see Fig. 3). Following meiosis, the resulting “haploid” nuclei (still endoreplicated to ~26N [Table 1]) are partitioned into uninucleate individuals that emerge from the parent test and grow into adults. Large uninucleate adults reset their genomes through *Zerfall* (see Fig. S5), a process that includes the elimination of the nuclear envelope to release chromatin into the cytoplasm and then the generation of threads that resolve into multiple haploid genome complements (Table 1). These haploid genomes are eventually surrounded with nuclear envelope and membranes to generate the amoeboid gametes that are typical of this species. Not shown here are life cycle stages in which haploid and diploid adults produce offspring of the same ploidy through a process called schizogony.

Based on fluorescence confocal microscopy analysis of 2,860 nuclei in 110 cells, we characterize nuclear architecture according to the number and structure of nuclei across life cycle stages (Table 1; File S3). Below, we describe the number and shape of nuclei and the state of both chromosomes (when visible) and chromatin structure within a nucleus. We first discuss general features shared across life cycle stages and then describe stage-specific features, beginning with Type 1 emergers—which we find are uninucleate—followed by Type 2 emergers, which are multinucleate (Table 1; Fig. 2).

**TABLE 1:** Image analysis on *A. laticollaris* CSH cells at different life stages shows variability in nuclear size and architecture as well as genome content^*a*^

Inferred life stage	Nuclear architecture	No. of cells imaged	No. of nuclei measured	Cell diameter (µm)	Nuclear volume (µm^3^)	Total fluor (MF)	DNA content (C)
Haploid complement^*b*^	H	3	707	2.6 × 10^2^	0.30(0.20–0.30)	0.15(0.087–0.20)	1.3(0.77–1.8)
Gamete	H	2	572	2.0 × 10^2^	6.3(4.8–7.2)	0.11(0.080–0.14)	1.0(0.70–1.3)
Zygote	H	10	556	3.0 × 10^2^ (2.7–3.0 × 10^2^)	13(9.0–20.)	0.22(0.13–0.34)	1.9(1.1–3.0)
Multinuc. juv. in parent test	HDP	10	264	1.9 × 10^2^ (1.8–2.7 × 10^2^)	51(25–98)	0.81(0.40–1.8)	7.2(3.5–16)
Multinuc. juvenile	H	15	69	36(31, 32, 34–44)	86(52–1.2 × 10^2^)	53(21–77)	4.7 × 10^2^(1.8–68 × 10^2^)
Multinuc. adult	V1	6	14	2.4 × 10^2^ (2.0–3.5 × 10^2^)	2.6 × 10^3^(1.6–4.5 × 10^3^)	55(22–140)	4.9 × 10^2^(1.9–12 × 102)
	V2	12	65	2.9 × 10^2^ (2.3–3.5 × 10^2^)	7.1 × 10^2^ (4.2–15 × 10^2^)	9.6(5.0–20.)	85(0.44–1.8 × 10^2^)
	MB	18	121	2.4 × 10^2^ (1.7–3.5 × 10^2^)	3.2 × 10^2^ (1.7–6.1 × 10^2^)	6.2(2.2–14)	55(20–120)
	H	8	37	2.0 × 10^2^ (2.0–2.5 × 10^2^)	1.5 × 10^2^ (1.0–2.5 × 10^2^)	3.0(1.6–9.0)	26(14–79)
	Other	3	11	1.7 × 10^2^ (1.7–2.4 × 10^2^)	4.4 × 10^2^ (3.4–6.1 × 10^2^)	7.8(5.0–15)	69(44–140)
Uninuc. juv in parent test	V	4	20	3.1 × 10^2^ (2.8–3.5 × 10^2^)	7.1 × 10^2^ (4.2–15 × 10^2^)	5.2(2.7–11)	46(24–100)
Uninuc. juvenile	V	10	10	63(50.–80.)	7.1 × 10^2^ (6.2–7.8 × 10^2^)	6.1 × 10^2^ (5.4–9.3 × 10^2^)	5.4 × 10^3^(4.3–8.2 × 10^3^)
Uninuc. adult	VB	8	8	1.2 × 10^2^ (1.0–1.3 × 10^2^)	3.7 × 10^3^ (2.0–5.3 × 10^3^)	1.2 × 10^3^ (0.70–1.4 × 10^3^)	11 × 10^3^(6.1–12 × 10^3^)
	VL	3	3	2.8 × 10^2^ (2.1–3.2 × 10^2^)	6.1 × 10^3^(5.6–21 × 10^3^)	1.0 × 10^3^ (0.64–3.2 × 10^3^)	29 × 10^3^(7.0–48 × 10^3^)
	*Zerfall*	6	6	2.3 × 10^2^ (2.1–2.9 × 10^2^)	7.0 × 10^3^ (5.5–9.0 × 10^3^)	1.1 × 10^3^ (0.42–1.4 × 10^3^)	10.0 × 10^3^(3.7–12 × 10^3^)
*Zerfall* cell	NA	17	NA	1.8 × 10^2^(1.7–2.4 × 10^2^)	*NA*	1.0 × 10^3^ (0.54–1.6 × 10^3^)	9.0 × 10^3^(4.8–14 × 10^3^)

^*a*^ *Allogromia laticollaris* CSH nuclear types are ordered according to their inferred place in the life cycle. For each nucleus type, we show the number of cells and nuclei measured. Cell diameter, nuclear volume, nuclear fluorescence are shown in millions of fluorescence units (MF), and DNA content is shown as the sample median (25th percentile–75th percentile). We calculate the DNA content (C) for *A. laticollaris* CSH nuclei as a proportion of the median nuclear fluorescence for gamete nuclei. Multinuc., multinucleate; Uninuc., uninucleate; H, homogeneous; HDP, homogeneous with DNA poor center; V, vegetative; V1, vegetative pre-division; V2, vegetative post-division; MB, meiotic bouquet; VB, vegetative with chromatin structure balls; VL, vegetative with chromatin structure lattice.

^*b*^ Note that the first row are small spheres in *Zerfall* cells, and not nuclei, and that these 3 cells are also counted among the 17 *Zerfall* cells at the bottom of the table.

**Fig 5:**
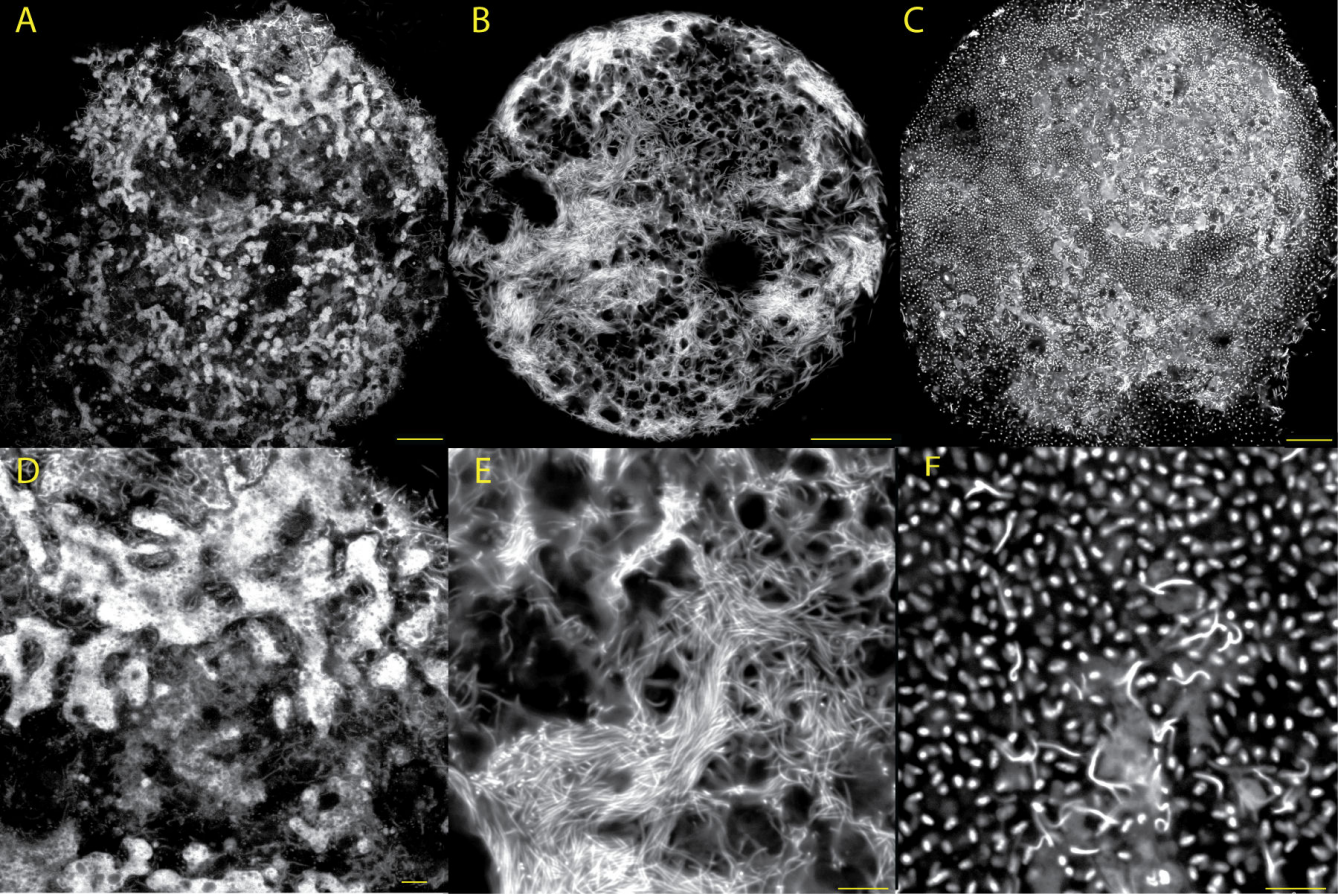
Details of the three types of Hoechst-positive structures observed in *Zerfall* cells. In order, panels A–C show three cells that represent our inferred progression of genome reorganization during *Zerfall*. Panels D–F show details of the three distinct Hoechst-positive structures within these three cells. DNA is shown in grayscale, and images have been enhanced for brightness and contrast (raw fluorescence measurements in File S3 and original images in File S4). Scale bars in panels A–C are 25 µm, and those in panels D–F are 5 µm. (A) Cell filled with globular chromatin structures. (B) Cell filled with threadlike DNA structures. (C) Cell filled with small spheres, inferred to be haploid genome complements. (D–F) Detailed regions of panels A–C, respectively. See Fig. S5 for additional images of *Zerfall* cells.

Generally, nuclei in adult *A. laticollaris* CSH are either round or oblong, with a brightly stained ring of chromatin around a DNA-poor center where condensed chromatin (possible chromosomes) are typically visible (Fig. 2). The *A. laticollaris* CSH karyotype is not known, but the structures we describe as chromosomes are consistent with previous reports from a different strain of this same species (20, 29). These observations are also consistent with those of Arnold (20), who described a denser, granular “nucleolar layer” restricted to the periphery of the nucleus, and a less dense “chromosome-bearing core.” The peripheral chromatin ring has two forms that we refer to as beaded or latticed: a beaded chromatin ring consists primarily of small, brightly stained spheroid structures (Fig. 2; Fig. S3A and B; Table 1), whereas a latticed chromatin ring appears more granular (Fig. 2; Fig. S3C and D; Table 1). Chromosomes are usually dispersed throughout the nucleus’s DNA-poor region (Fig. 2; Fig. S3). We refer to the combination of a chromatin structure ring and a DNA-poor region with identifiable chromosomes as “vegetative” nuclear architecture, as this is most common in adult stages (Table 1).

We find that Type 1 (Fig. 1A) emergers have a single nucleus. When these uninucleate cells are within the parent test prior to emergence, they have a large (volume of 710 µm^3^ on average) nucleus (Table 1) with vegetative nuclear architecture. Nuclear architecture remains vegetative during emergence and the uninucleate juvenile stage (Table 1; Fig. 2). Uninucleate adults have substantially larger nuclei (median 5,400 µm^3^), and at this stage, the beaded chromatin structure (VB) is more common than latticed (VL in Table 1). While most uninucleate adults have vegetative nuclei, we imaged six cells with large nuclei (median 7,000 µm^3^) in which the latticed chromatin structure is spread throughout the nucleus, which might represent early stages of *Zerfall* (*Zerfall* in Table 1; Fig. 2).

**Fig 3:**
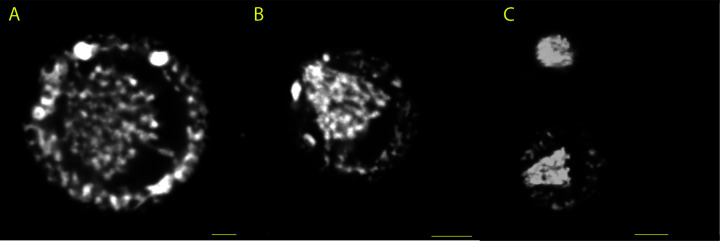
Inferred transitions in nuclear architecture during meiosis in *A. laticollaris* CSH. All nuclei derive from multinucleate adult cells with heterogeneous nuclear architecture. Nuclei in panels A and B derive from the same cell. All scale bars are 5 µm; see Fig. S4 for detailed breakdown of nuclear architecture in all multinucleate adult cells imaged in the study. (A) V2 nucleus with a condensed chromatin ring around a DNA-poor center with central chromosomes. We infer that V1 nuclei amitotically divide to form V2 nuclei. (B) MB nucleus with a condensed chromatin ring around a DNA-poor center with a meiotic chromosome bouquet. We infer that MB nuclei represent a meiotic prophase that begins in V2 nuclei. The V2 nucleus in panel A and the MB nucleus in panel B derive from the same cell (“adult with meiotic nuclei; Fig. 2). (C) H nucleus (top) with condensed chromatin and no DNA-poor region. We infer that H nuclei are the products of meiosis based on their genome content, which is reduced compared with that of other nuclear types in multinucleate adult cells and similar to that of developing uninucleate juveniles (Table 1). The bottom structure is an MB nucleus within the same cell.

Type 2 reproductive cells (Fig. 1B) produce offspring that possess multiple nuclei with diverse architectures at different life stages. While inside the parent test, the nuclei in multinucleate juveniles are ~51 µm^3^ with homogeneously staining chromatin and a small DNA poor region in the center (HDP in Table 1; Fig. 2). After emerging from the parent, multinucleate juveniles typically possess 4–23 smaller (median volume 86 µm^3^) nuclei with homogeneous, brightly staining chromatin (Table 1; Fig. 2). Nuclei in multinucleate adults can present in up to four types of nuclear architecture within a single cell (Table 1; Fig. 2; Video S1): (i) V1 nuclei—which we infer are vegetative nuclei prior to division—are larger (median 2,600 µm^3^) and oblong, (ii) smaller V2 nuclei (median 710 µm^3^), which we infer are the vegetative products of a nuclear division (likely a non-canonical amitotic division given the endoreplicated genome) of V1 nuclei, (iii) meiotic bouquets (MB in Table 1) where chromosomes are attached to a narrow span of the nuclear periphery and fanned out into the center (Table 1; Fig. 3 ), consistent with previous descriptions of meiosis in *Allogromia* (20) as well as in other foraminifera (34, 35), and (iv) H nuclei that are smaller (median 150 µm^3^) with homogeneously-staining condensed chromatin that are likely products of meiosis. Multinucleate adult cells typically contain either all V1 nuclei, all MB nuclei, or a combination of V2, MB, and H (Fig. 3; Fig. S4). Multinucleate adults occasionally contain nuclei with a beaded chromatin structure ring and a circular mass of condensed chromosomes in the center of the nucleus (“Other” in Table 1; Fig. S4); this architecture is uncommon and was only observed in combination with V2 and MB architectures (Table 1; Fig. S4).

### Transcription occurs in the DNA poor region of vegetative nuclei

**Fig 4:**
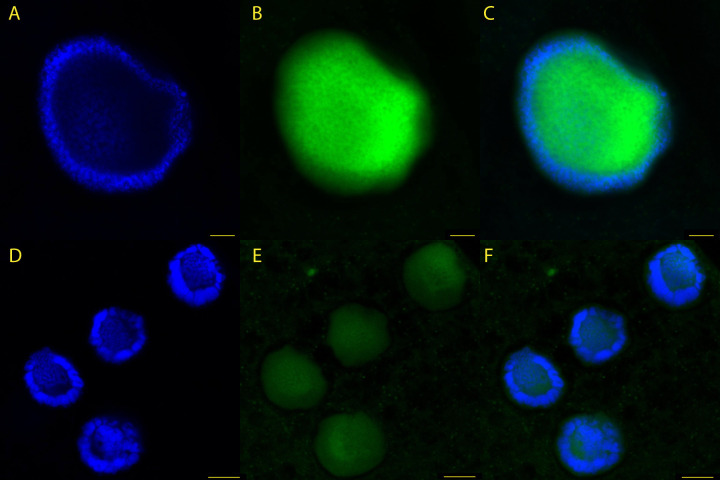
Inverse relationship between concentrations of DNA and newly transcribed RNA in *A. laticollaris* CSH nuclei. *A. laticollaris* CSH cells were stained with Hoechst 33342 (blue) to visualize DNA and an azide molecule bound to an AlexaFluor 488 probe (green) which binds to modified uracils to visualize all newly transcribed RNA. Scale bars in panels A–C are 5 µm, and scale bars in panels D–F are 7.5 µm. Images are enhanced to increase brightness and contrast; original images are in File S4. (**A**) Hoechst-stained nucleus in a uninucleate cell, which shows greater intensity at the nuclear periphery. (**B**) AlexaFluor-stained nucleus in a uninucleate adult cell, which shows greater intensity at the nuclear center. (**C**) Overlay image. (**D**) Hoechst-stained nuclei in a multinucleate adult cell, which show greater intensity at the nuclear periphery. (**E**) AlexaFluor-stained nuclei in multinucleate cells, which show greater intensity at the nuclear center; the bright spot of extranuclear signal in the top center of the image likely comes from autofluorescence of partially digested algal food, whereas dim extranuclear signal likely represents cytoplasmic RNAs. (**F**) Overlay image.

We use the Click-iT RNA AlexaFluor 488 Imaging Kit to assess the location of active transcription in adult *A. laticollaris* CSH cells. In both uninucleate and multinucleate cells, DNA stained with Hoechst and RNA stained with AlexaFluor 488 azide show inverse concentrations (Fig. 4). Hoechst staining reveals that *A. laticollaris* CSH nuclei concentrate DNA at their periphery (Fig. 4A and D), with nascent RNAs abundant in the DNA poor center (Fig. 4B and E).

### Exceptions in nuclear architecture of *Allogromia laticollaris*

There are several important exceptions to these common types of nuclear architecture, and we discuss these in two categories: those with large numbers (i.e. >100) of nuclei, which we interpret as gametes and zygotes (see section on fluorescence quantification below), and those that are DNA rich but lack an obvious nuclear envelope, which we infer are in *Zerfall* (see “*Allogromia laticollaris* CSH uses non-canonical mechanisms to reset ploidy levels” in Discussion). We document 17 adult cells (median 180 µm diameter) with the latter architecture, within which we identify combinations of three distinct Hoechst-positive structures (Table 1; Fig. 2 and 5): dense, globular fragments similar to the chromatin structures within uninucleate adult nuclei (Fig. 5A; Video S2), brightly staining threads that are 0.25–0.35 µm wide on average (Fig. 5B; Video S3), and small, punctate spheres (Fig. 5C; Video S4). All 17 cells with this putative *Zerfall* architecture originate from the same culture flask (containing 100s of cells) and were identified within a 2-week period, supporting our observations of life stage synchrony described above and further implicating *Zerfall* as a brief, albeit radical, genome reorganization process. Light microscopy shows that the majority of these cells had uneven pigmentation, unusual shape, and/or separation between the cytoplasm and test.

### DNA content dramatically increases throughout the life cycle of *A. laticollaris*

**Fig 6:**
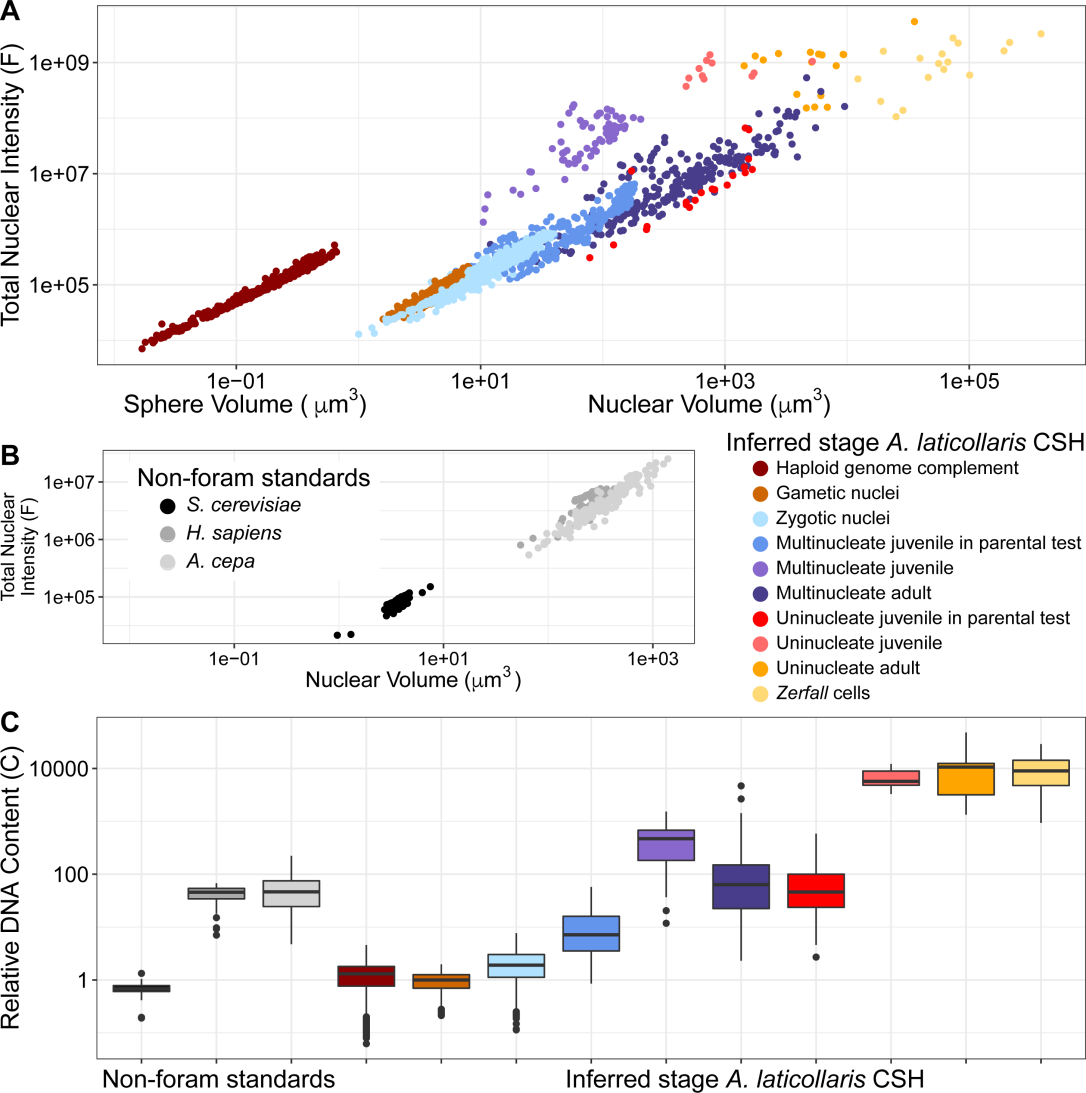
Total fluorescence and estimated DNA contents of *Allogromia laticollaris* CSH nuclei across life cycle stages (colored) show endoreplication and can be compared with non-foraminifera standards (gray scale). Warm-toned colors denote haploid stages (Type 1 cells), and cool-toned colors denote diploid stages (Type 2 cells). The maroon-colored data refer to fluorescent spheres in *Zerfall* cells. (**A and B**) Scatterplots comparing the total fluorescence from Hoechst per nucleus to the total nuclear volume in *A. laticollaris* CSH cells (**A**) and standards (yeast, human, and onion) (**B**). Each point represents a nucleus, and points are colored by inferred life stage. Both axes are shown on a log scale. (**C**) Boxplots comparing estimated DNA content across life stages in *A. laticollaris* CSH cells and standards. Each boxplot contains data from all the nuclei measured during a particular life stage. The *y*-axis (log scale) represents DNA content relative to the median estimate of the *A. laticollaris* CSH haploid genome size, i.e., 1C is the haploid genome size. See File S3 for fluorescence measurements.

To document how DNA content varies across *A. laticollaris* CSH life stages, we imaged and analyzed 2,860 nuclei from 110 individuals displaying dramatic variation in nuclear size and DNA content (Table 1; Fig. 2 and 6; File S3). The nuclei range in volume from 0.25 to 36,000 µm^3^ (a 144,000 fold range) and their total fluorescence varies by 475,000-fold. This reflects the dramatic scale of endoreplication during the *A. laticollaris* CSH life cycle (Table 1).

We note cells with hundreds of nuclei as containing gametes and zygotes, as these nuclei possess the lowest volume and fluorescence of any life cycle stage we observed (Table 1; Fig. 6A). Gametic nuclei are enclosed in membranes and surrounded by cytoplasm (Fig. 2), consistent with Arnold’s description of gametogenesis in *A. laticollaris* (20), and are 6.3 µm^3^ and 110 KF on average (Table 1; Fig. 6A). We set this fluorescence value as putatively haploid (i.e., 1C) (Fig. 6C) and use it as the point of comparison for all remaining life cycle stages. Zygotic nuclei lack cytoplasmic boundaries (i.e., they occur in shared cytoplasm within the parent test; Fig. 2) and on average are 13 µm^3^ with DNA content 1.9C (Table 1; Fig. 6).

Image analysis reveals that substantial nuclear growth and endoreplication take place following the zygotic stage. We infer that cells containing zygotes undergo cytokinesis to become Type 2 reproductive cells, which contain multinucleated offspring. The multinucleated juveniles developing within the parent test (Fig. 2) contain nuclei that are 51 µm^3^ and 7.2C on average (Table 1; Fig. 6). Nuclear growth continues once these cells emerge from the parent, increasing from 51 µm^3^ and 7.2C to 86 µm^3^ and 470C on average (Table 1; Fig. 6). Once mature, these multinucleated adult cells have the most diverse nuclear architecture and display the widest range in nuclear volume and DNA content (Table 1; Fig. 2; Fig. S4). V1 nuclei in these cells are the largest and most DNA rich (2,600 µm^3^ and 490C on average; Table 1; Fig. 6), with approximately nine times the DNA content of MB nuclei and 19 times the DNA content of H nuclei (Table 1; Fig. 6). V1 nuclei are typically in large (~240 µm diameter) cells with two to five nuclei of the same architecture, and H nuclei are found in cells with 5–18 nuclei of mixed architecture (Fig. S4; File S3).

We find that uninucleate Type 1 offspring develop the highest overall DNA content per nucleus (up to ~12,000C). Nuclei in uninucleate juveniles contained within Type 1 reproductive cells are 710 µm^3^ and 46C on average (Table 1; Fig. 6). Much like multinucleated cells, once these offspring emerge from their parent, they experience a substantial increase in DNA content, but not nuclear volume, relative to cell volume: increasing from 46C to 5,400C on average (Table 1; Fig. 6). Prior to *Zerfall* and gametogenesis, uninucleate adults have the greatest nuclear DNA content across all cell types, with approximately 11,000C on average (Table 1; Fig. 6).

We also measure total fluorescence intensity from Hoechst in the 17 “exceptional” adult cells that lack a nuclear envelope, a stage consistent with Arnold’s description of *Zerfall* (20) (see “*Allogromia laticollaris* CSH uses non-canonical mechanisms to reset ploidy levels” in Discussion). Strikingly, on average, the total DNA content throughout an entire *Zerfall* cell is similar to the DNA content in uninucleate adult nuclei (Table 1; Fig. 6C). These cells contain 9,000C on average with DNA dispersed throughout the cytoplasm (Table 1; Fig. 6C). We also measure the per-object fluorescence of 707 small, brightly staining spheres in *Zerfall* cells (Fig. 2 and 5C), treating them as individual nuclei. Interestingly, we find that these structures contain approximately the same total fluorescence as gametic nuclei, despite being >20 times smaller in volume on average (Table 1; Fig. 6A).

### Estimating genome size in *Allogromia*

We use our image analysis pipeline on three lineages (human, onion [*Allium cepa*], and yeast [*Saccharomyces cerevisiae*]) with known genome size to generate a preliminary estimate of the *A. laticollaris* CSH genome size; however, the high AT bias of the *A. laticollaris* CSH genome coupled with vast scales of genome amplification creates uncertainty in our estimates. We analyze 81 *Saccharomyces cerevisiae* nuclei (38% GC), 64 *Homo sapiens* nuclei (41% GC), and 179 *Allium cepa* nuclei (34% GC) to capture a range of eukaryotic genome sizes (Fig. 6B). As described in “Image analysis” in Materials and Methods, we use two different methods to estimate the haploid genome size in *A. laticollaris* CSH (Table S4): method 1 uses the ratio of base pairs per fluorescent unit in *S. cerevisiae* to estimate the haploid genome size, and method 2 uses the average ratio of base pairs per fluorescent unit across the three standards. Given the similarity in size and fluorescence of our gametic nuclei to *S. cerevisiae*, we use method 1 and estimate the haploid genome size in *A. laticollaris* CSH as 34 Mb (24–43 interquartile range [IQR]) and the diploid genome as 64 Mb (38–100 IQR) (Table S4).

Using these estimates, we calculate that the nuclear genome size varies almost 12,000-fold throughout the life cycle (Table 1; Table S4). We hypothesize that substantial endoreplication occurs following the diploid zygote stage, since vegetative nuclei in multinucleated adults reach 17 Gb on average (7–40 IQR; Table S4). We estimate higher per-nucleus DNA content during uninucleate (Type 1) stages, at 361 Gb on average (210–410 IQR) in vegetative adult cells (Table S4). However, we emphasize the relative differences in DNA content among life cycle stages rather than absolute genome size measurements given the well-recognized limitations and biases in fluorescence-based estimates of genome size. For instance, Hoechst has a demonstrable AT bias (36), and the *Allogromia* genome likely has GC content ~27% (based on unpublished transcriptomic data) which is lower than that of the three lineages used as standards.

## DISCUSSION

### Haploidy and diploidy are but brief stages in the *Allogromia laticollaris* CSH life cycle

Combining fluorescence microscopy and image analysis, we show that haploidy and diploidy are brief transient stages in the life cycle of *A. laticollaris* CSH; instead, the majority of life cycle stages are marked by nuclei with substantially endoreplicated genetic material (Table 1; Fig. 2 and 6). Our inferences on euploidy (i.e., haploid vs diploid) are based on our knowledge of life cycle progression from longitudinal observations (Fig. 1), relative nuclear size and fluorescence (Table 1; Fig. 6), and previous literature on *A. laticollaris* (17, 20, 29). In our model, true haploid nuclei emerge following *Zerfall* (see section below), appearing within the parental cell as amoeboid uninucleate gametes (Fig. 2). These have the lowest total fluorescence of all nuclei measured (Table 1; Fig. 6A), and their morphology is consistent with Arnold’s description of haploid gametes (20). The median haploid genome size estimate based on the fluorescence in these nuclei is 34 Mb (Table S4), roughly 5.5 times the haploid genome size of the yeast *Saccharomyces cerevisiae* (6 MB), though we acknowledge caveats behind these calculations including high AT bias in *A. laticollaris* genes. Across the 1,000s of cells observed and 110 cells imaged, we see only two cells with nuclear architecture consistent with gametes (Table 1), leading us to hypothesize that haploidy is a brief moment within the *A. laticollaris* life cycle; this observation concurs with those of Lee and McEnery (31).

True diploidy is a similarly brief stage within the *A. laticollaris* life cycle (Table 1; Fig. 2). We infer that uninucleate, haploid gametes undergo plasmogamy and karyogamy to produce cells filled with hundreds of diploid zygotic nuclei. Similar to haploid nuclei, diploid nuclei are spherical with condensed, homogeneously staining chromatin (Table 1; Fig. 2). On average, these nuclei have 1.9 times the total fluorescence per nucleus as haploids, consistent with our hypothesis that they represent the true diploid state (Table 1; Fig. 6C). This stage, like haploidy, is short lived as we see few cells with this architecture (10 of 110 cells analyzed) across all experiments over the course of a year. Our inferences of haploid uninucleate and diploid multinucleate stages concur with those of Arnold (20) and with foraminiferal life cycles in general (reviewed in reference 10). McEnery and Lee (29) estimated that both uninucleate and multinucleate stages are diploid, but this study relied only on microspectrophotometric measurements of ploidy, which would be misleading given the endoreplication described here.

In all other life cycle stages, the genome content in *A. laticollaris* CSH is substantially greater than expected for canonical haploid-diploid cycles. *Allogromia laticollaris* CSH experiences rapid genome endoreplication as it grows (30), as evidenced by elevated DNA content that corresponds to increases in nuclear volume in both uninucleate and multinucleate stages (Table 1; Fig. 6). Combining our observations with prior knowledge of foraminifera (10, 20, 21), we infer that uninucleate cells contain nuclei with endoreplicated haploid genomes (N) while multinucleate cells contain nuclei with endoreplicated diploid genomes (2N; Table 1; Fig. 2 and 6 ). In their adult stages, vegetative nuclei within multinucleate and uninucleate cells contain ~490 and ~11,000 times the haploid genome content, respectively (Table 1; Fig. 6C). These measurements demonstrate that endoreplication, either by whole-genome duplication or by partial genome amplification (23), occurs consistently in *A. laticollaris* CSH in both haploid and diploid stages.

The elevated DNA content beyond diploidy is not uncommon in eukaryotes, particularly among microbial lineages. The genome content increases in the life cycle of *Amoeba proteus* (8, 37), up to 40-fold in *Entamoeba histolytica* (38) and up to 2,000-fold in *Aulacantha scolymantha* (Rhizaria, formerly Radiolaria [39]). Endoreplication also occurs in somatic cells of plants and animals (5, 40, 41) and in the somatic nuclei of ciliates where amplification can be 1,000-fold (7, 42). Thus, the endoreplication in *A. laticollaris* CSH nuclei is consistent with other eukaryotic life cycles; more intriguing is the fact that endoreplicated stages comprise the majority of the life cycle while true haploidy and diploidy are brief.

In some multinucleated cells, we find evidence of centralized chromosomes undergoing meiosis (i.e., forming a meiotic bouquet) while being surrounded by a brightly stained chromatin ring (Fig. 2 and 3; Table 1; Fig. S4; Video S1). The observations of meiotic bouquets (Fig. 2 and 3) are consistent with those reported for *Allogromia* (17, 18, 20); more broadly, chromosome bouquets are argued to associate with meiosis in other monothalamid foraminifera (35, 43). In a subset of cells with nuclei containing meiotic bouquets, we also observe small nuclei with condensed, homogeneously staining chromatin that we infer are the products of meiosis; these H nuclei contain approximately half the DNA content of meiotic bouquets and approximately the same DNA content of some nuclei within uninucleate offspring that result from “schizogony” (i.e., cytoplasmic division in context of same euploidy) in foraminifera (Table 1; Fig. S4). Yet, the DNA content within these “haploid” nuclei is ~26C (Table 1), indicating that endoreplication persists throughout meiosis as chromosomes in the center of the nucleus reduce their ploidy while the chromatin structure at the nuclear periphery is maintained.

### *Allogromia laticollaris* CSH uses non-canonical mechanisms to reset ploidy levels

We infer that *Allogromia laticollaris* CSH resets its ploidy from an endoreplicated haploid state to true haploidy through *Zerfall*, a process by which the nuclear envelope breaks down and chromatin is extruded into the cytoplasm. Originally described by Føyn (14), few observations of *Zerfall* have been made. Føyn’s original description was based on four uninucleate *Myxotheca arenilga* cells that lacked chromatin, two of which had enlarged nuclei with irregular distribution of nucleolar material, and the other two of which were filled with a fine-meshed framework that might be analogous to the threads we document in *Allogromia laticollaris* CSH (14). The differences in approaches between these two studies, which are nearly 90 years apart, make comparisons challenging.

Our observations on life cycle duration and synchrony (Fig. 1; Tables S2 and S3; File S2) enable us to predict when a population will undergo *Zerfall* and identify characteristic morphological features, allowing us to infer the steps of *Zerfall* through analyses of 17 cells (Table 1; Fig. 2 and 5; Fig. S5). *Zerfall* begins within a uninucleate cell with a large (39–74 µm) nucleus that has vegetative architecture and has endoreplicated beyond 10,000 times the haploid genome content (Table 1; Fig. 2). First, the nuclear architecture changes such that the chromatin at the nuclear periphery disperses throughout the normally DNA-poor center (“*Zerfall* nucleus” in Fig. S5A). Karyolysis then takes place, in which the nuclear envelope disappears, extruding Hoechst-positive material into the cytoplasm. (Fig. 2; Fig. S5B through F). In the cells that we infer are at the early stages of karyolysis, we observe dense, globular structures—similar to the chromatin structure within the early *Zerfall* nucleus—distributed throughout the cell (Fig. 2 and 5A; Fig. S5E through O; Video S2). We hypothesize that these structures are transformed into Hoechst-positive threads (Fig. 5B), since z-stacks reveal cells with either and both types of structures (Fig. S5E and F; Videos S3; S4). Dahlgren (15) reported nuclear degradation in the monothalamid *Ovammina opaca*, followed by the presence of “basiphil granules and strands” in the cytoplasm, which may be similar to the threads that we detect during *Zerfall* in *Allogromia*. In a subset of cells with threads, the threads are interspersed with clusters of punctate, brightly staining spheres that we hypothesize are formed by the division of the threadlike chromatin (Fig. 2; Fig. 5C; Fig. S5L through P; Video S4). We infer that these structures are compacted haploid genomes—which we refer to from here on as haploid genome complements, as each contains approximately the same DNA content as a haploid gamete despite being an order of magnitude smaller in volume (Table 1; Fig. 6C). We hypothesize that haploid genome complements later develop into gametes via growth and division of the parental cytoplasm (Fig. 2; Fig. S5Q).

Our data are inconsistent with aspects of *Zerfall* described by several authors including work on the genus *Allogromia* (20) and other species (*Ovammina opaca* [15], *Iridia lucida* [18], and *Saccamina alba* [26]). Arnold (20) proposed that *Zerfall* occurs in *A. laticollaris* when a uninucleate haploid cell extrudes endoreplicated chromatin into the cytoplasm to be eliminated, while the chromosomes undergo rapid mitotic divisions to produce gametes. Arnold (20) saw “nucleolar” material in cytoplasm but concluded it might be pathological or necrotic. Goldstein (21) documented *Zerfall*-like processes in *Triloculina oblonga* and *Ammonia tepida* in which a large nucleus degrades, and its remnants co-occur in the cytoplasm with “pre-gametic” nuclei, some in stages of mitosis, that she argued rapidly divide and differentiate into gametes. We see neither canonical chromosomes nor mitotic figures within any *Zerfall* cell. Furthermore, the overall DNA content of the material within a *Zerfall* cell is similar to that of the nuclear DNA content prior to *Zerfall* (Table 1; Fig. 6C). Taken together, this suggests that rather than simultaneously eliminating and replicating chromatin, *A. laticollaris* CSH reorganizes its endoreplicated chromatin to generate gametic nuclei as the chromatin structure transforms into threads of DNA that resolve into haploid genome complements (Table 1; Fig. 2; Fig. S5). It may be that gametic nuclei undergo mitosis prior to karyogamy, perhaps to ensure replication of centromere-containing chromosomes, but we did not observe this in our study. Using TEM, other authors have documented gametogenic mitosis in foraminifera: *Allogromia laticollaris* (17), *Myxotheca arenilega* (44), *Iridia lucida* (45), and *Hastigerina pelagica* (46); mitosis has also been illustrated in agamonts (comparable to Type 2 reproductive cells of this report) of *Allogromia laticollaris* (47).

We suggest that the primary purpose of *Zerfall* is to reset ploidy levels from a highly endoreplicated genome to true haploidy and that this process extends the known diversity of eukaryotic genome organizations. Diverse lineages incorporate ploidy reduction into their life cycles, including Apicomplexa (Alveolata [48], Phaeodaria (formerly Radiolaria, Rhizaria [39]), *Amoeba proteus* (Amoebozoa [8, 37]), and the somatic genomes of ciliates (Alveolata [7, 33, 42]). Intriguingly, these mechanisms parallel events in some cancer cells in which increases in ploidy levels are followed by reduction of genome through fragmentation into micronuclei or through chromothripsis (41, 49, 50).

### Transitions between life cycle stages in *Allogromia laticollaris* CSH appear synchronous while nuclear divisions are asynchronous

Combining light and fluorescence microscopy, we expand the understanding of life stage synchrony in *A. laticollaris* CSH, which both Arnold (20) and McEnery and Lee (29) observed. Life stage durations are similar across cells in isolated and communal environments (i.e., single wells and flasks; Tables S2 and S3; Fig. 1). We infer that Type 1 emergers are haploid and uninucleate and observe that they have life cycles with greater and more variable lengths than diploid, multinucleate Type 2 emergers (Table S2; Fig. 1), consistent with observations from Schwab (47). Variance in life cycle duration may relate to variance in offspring ploidy; it is more common for haploid Type 1 emergers to produce haploid offspring than for diploid Type 2 emergers to produce diploid offspring (File S2; Fig. 1). Interestingly, light microscopy and fluorescence microscopy also reveal that life stage transitions, including reproduction, occur relatively synchronously among cells in communal environments (i.e., flasks; Table S3).

Life cycle synchrony, particularly surrounding gametogenesis and gamete release, is not uncommon among microbial eukaryotes. Environmental factors including nutrient levels, light intensity, water depth, and food source can drive life cycle synchronization and increase reproductive success (51–53). For example, the planktonic foraminifera *Hastigerina pelagica* and *Globigerinoides sacculifer* synchronize their life cycles with the lunar cycle, *Rotaliella elatiana* completes gamontogamy at night (54), and *Trochammina hadai* synchronizes its life cycle with the seasons (reviewed in reference 10). The apicomplexan *Plasmodium falciparum* has been shown to synchronize its life cycle with the host’s melatonin levels (53), and the red alga *Galdieria sulphuraria* synchronizes its life cycle based on light-dark cycles (52). The basis of synchronization of *A. laticollaris* CSH is unclear; however, given that cultures are stored in the same controlled environment, it is possible that light-dark cycles and/or nutrient availability drive patterns in this species.

Although we find that cell cultures largely undergo life cycle transitions synchronously, nuclear divisions within cells appear to be asynchronous. We document a plurality of nuclear architectures in multinucleated cells, which we hypothesize represent stages of meiotic division given their relative differences in DNA content and the presence of meiotic bouquets (Table 1; Fig. 2; Fig. S4). Asynchronous nuclear divisions in multinucleated cells are relatively uncommon in eukaryotes. Examples of asynchronous mitoses in the filamentous fungus *Ashbya gossypii* (55) and the malarial parasite *Plasmodium falciparum* (48) are argued to more efficiently connect divisions to resource availability. To the best of our knowledge, asynchronous meiotic cycles have not been well documented, adding to knowledge of genome flexibility within *A. laticollaris* CSH and among eukaryotes more broadly.

### Synthesis

Combining observations of nuclear architecture and measurements of DNA content across the life cycle, we describe the variation in DNA content and nuclear architecture in *Allogromia laticollaris* strain CSH. We find that “true” haploidy and diploidy are brief stages and that the genome of this species is endoreplicated throughout the bulk of the life cycle (Table 1; Fig. 2); this likely enables *Allogromia laticollaris* CSH to grow to large sizes (diameters of >300 µm; Table 1) and perhaps also to respond to environmental changes. Within vegetative nuclei, we observe the separation between distinct endoreplicated chromosomes in the center where transcription occurs and quiescent, condensed chromatin at the periphery (Fig. 2; Fig. S3). We also observe canonical chromosomes (“soma” Fig. 2; Fig. S3) participating in meiosis (Fig. 3) while surrounded by a highly replicated and quiescent chromatin structure (“germline;” Fig. 2; Fig. S3). Only in *Zerfall* (Fig. 2 and 5; Fig. S5) are these structures rearranged through the dispersal of genetic material throughout the cell’s cytoplasm in the absence of a nuclear membrane (Fig. 2 and 5; Fig. S5). We hypothesize that the cell recycles or eliminates its somatic chromosomes during *Zerfall* and that the germline chromatin structure gives rise to haploid genome complements and later, gametes. More broadly, we speculate that *Allogromia laticollaris* CSH relies on spatio-temporal mechanisms to distinguish germline from somatic DNA, a phenomenon that may be widespread in eukaryotes (3, 4).

As additional foraminifera are studied using fluorescence microscopy, we will be able to assess the extent to which the nuclear and genome dynamics within *A. laticollaris* are typical for this ancient group. Studies using light and transmission electron microscopy suggest that there are more “exceptions” to be documented. For example, Arnold (56) reports that daughter nuclei arise from “subnuclei” that exist within parental cells of the monothalamid *Psammophaga simplora* (56). Other taxa such as the globothalamids *Rotaliella heterocaryotica* (34) and *Rotaliella elatiana* (57) and the tubothalamids *Sorites orbiculus* and *Amphisorus hemprichii* (58) are reported to have distinct germline and somatic nuclei within every single-celled individual, yet these systems have yet to be studied with modern methods. Virtually, nothing is known about life cycles of other foraminifera, like the large multinucleated freshwater *Reticulomyxa* or the deep sea Xenophyophores. The ability to combine fluorescence microscopy and single-cell omics to these poorly studied lineages will be critical for elucidating their life cycles and placing them into the broader context of eukaryotic evolution.

## SUPPLEMENTAL MATERIAL

File S1 mbio.03379-23-s0001.xlsx Raw data from light microscopy.

File S2 mbio.03379-23-s0002.xlsx Raw data from light microscopy.

File S3 mbio.03379-23-s0003.xlsx Raw data from confocal scanning laser microscopy.

File S4 mbio.03379-23-s0004.pdf Images from confocal scanning laser microscopy.

Supplemental material mbio.03379-23-s0005.pdf Supplemental tables, figures, and file notes.

Video S1 mbio.03379-23-s0006.mp4 Confocal Z-stack.

Video S2 mbio.03379-23-s0007.mp4 Confocal Z-stack.

Video S3 mbio.03379-23-s0008.mp4 Confocal Z-stack.

Video S4 mbio.03379-23-s0009.mp4 Confocal Z-stack.

ASM does not own the copyrights to Supplemental Material that may be linked to, or accessed through, an article. The authors have granted ASM a non-exclusive, world-wide license to publish the Supplemental Material files. Please contact the corresponding author directly for reuse.
