# Supplementary material for: Foraminifera as a model of eukaryotic genome dynamism
Source: mBio. 2024 Feb 8;15(3):e03379-23. doi: 10.1128/mbio.03379-23 (PMC10936158; doi:10.1128/mbio.03379-23)
Supplement: File S4 — Images from confocal scanning laser microscopy. [file mbio.03379-23-s0004.pdf]

Supplementary File 4: Raw fluorescent images shown in Figs. 2, 3, 4, S2, S4. Images are labeled with the figure(s) in which they appear.

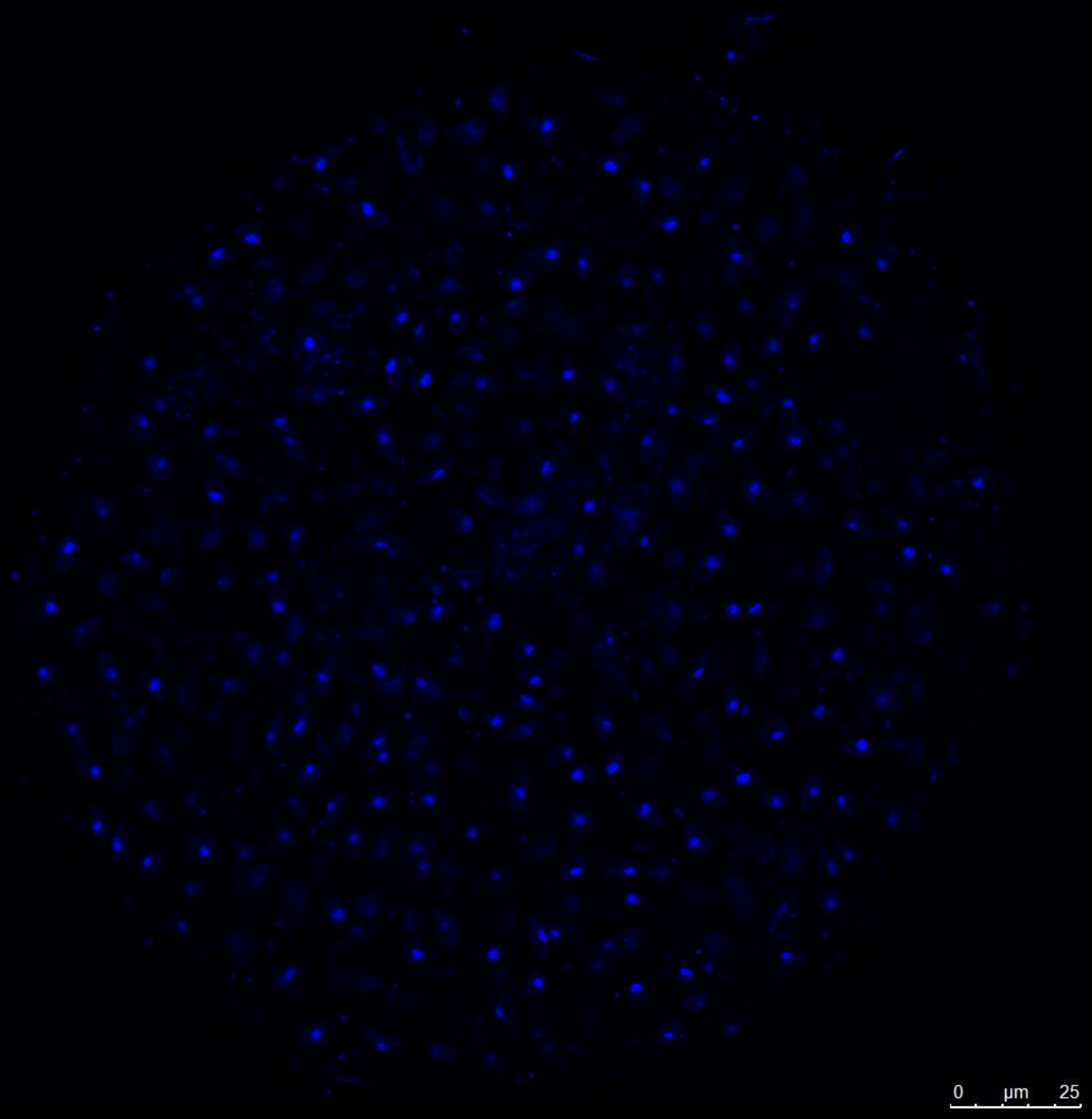

"Gamete" in Fig. 2; Fig. S4Q

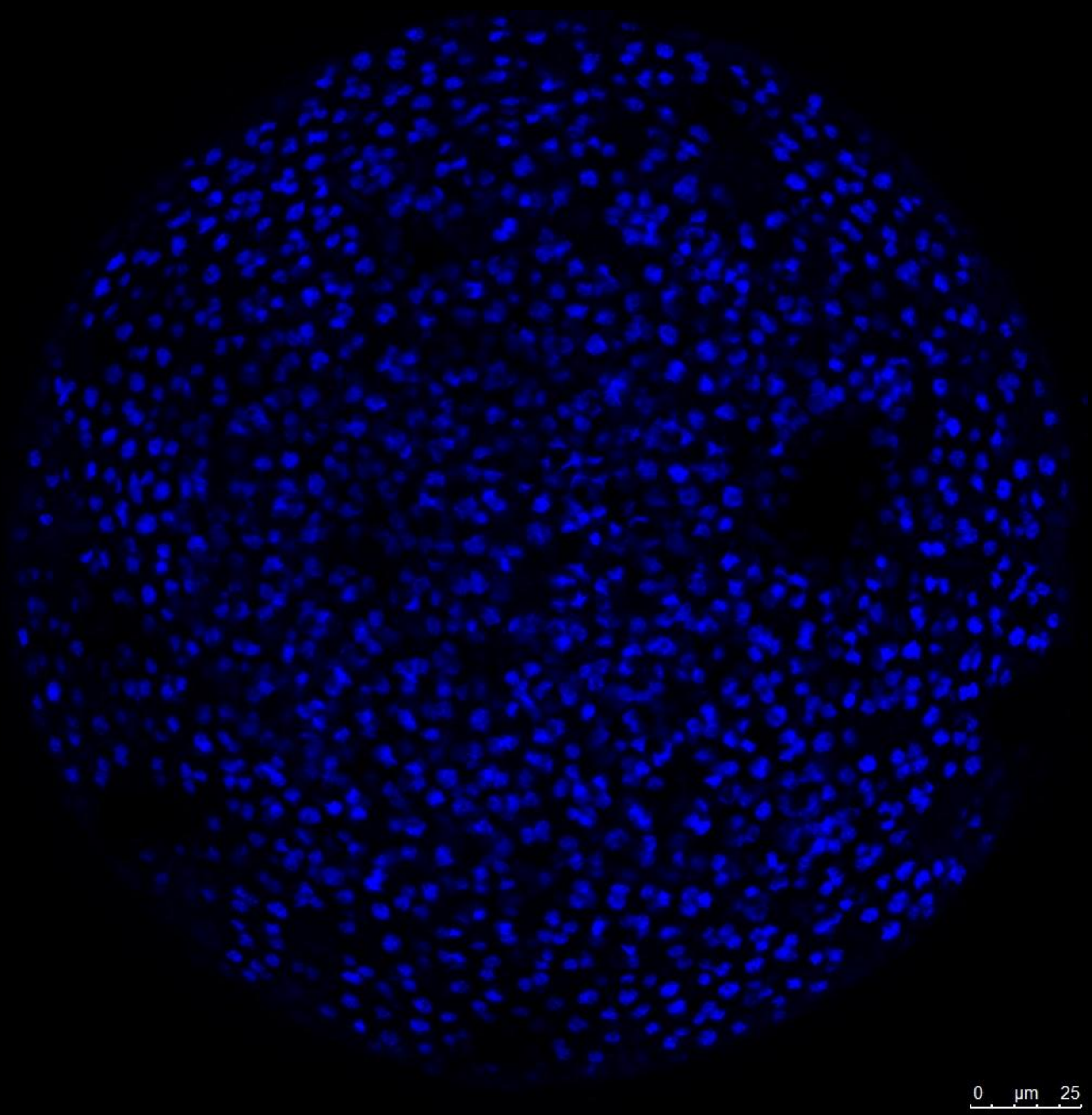

“Zygote” in Fig. 2; Fig. S4R

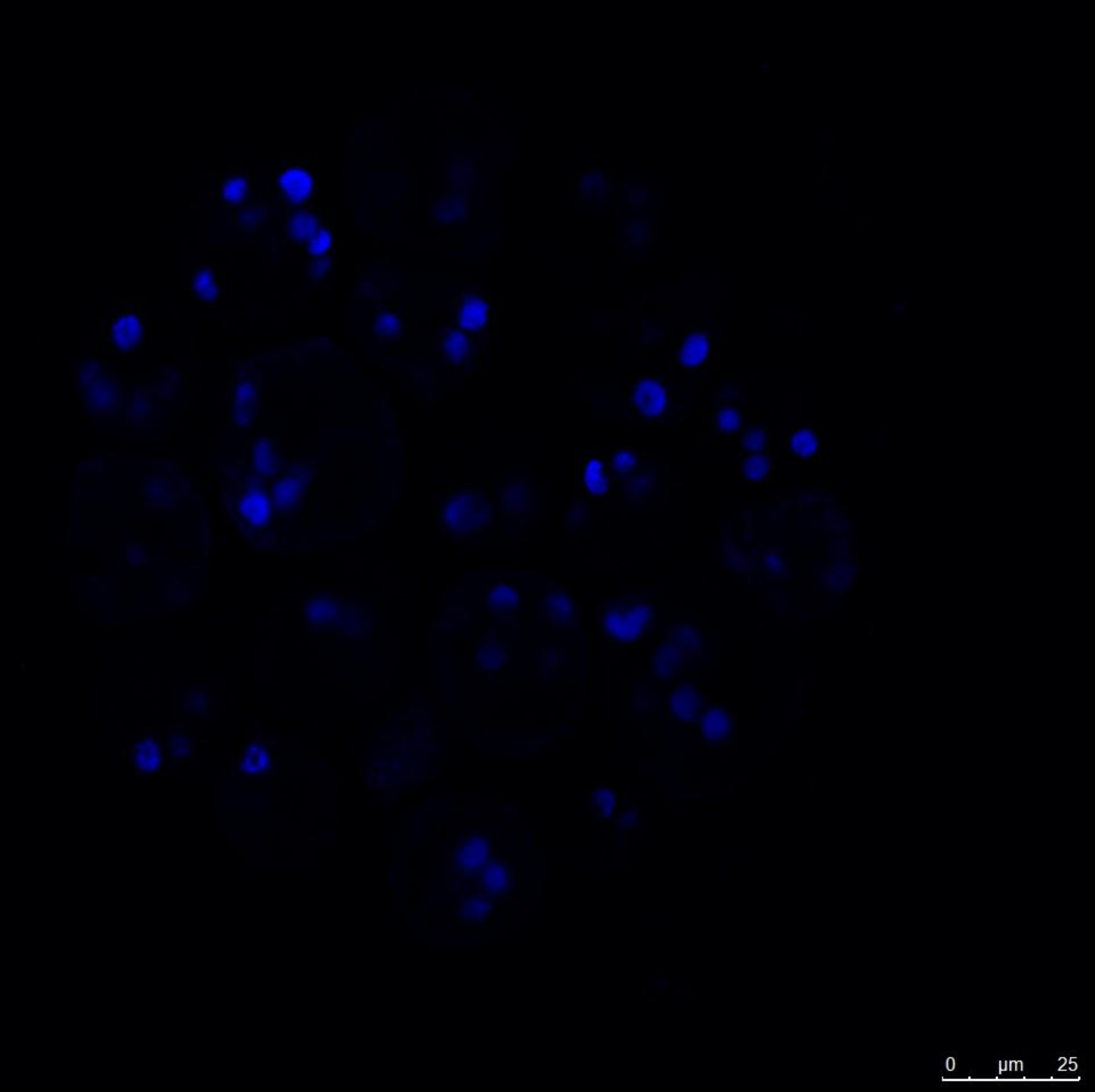

“Multinucleate juvenile (in parent test)” in Fig. 2

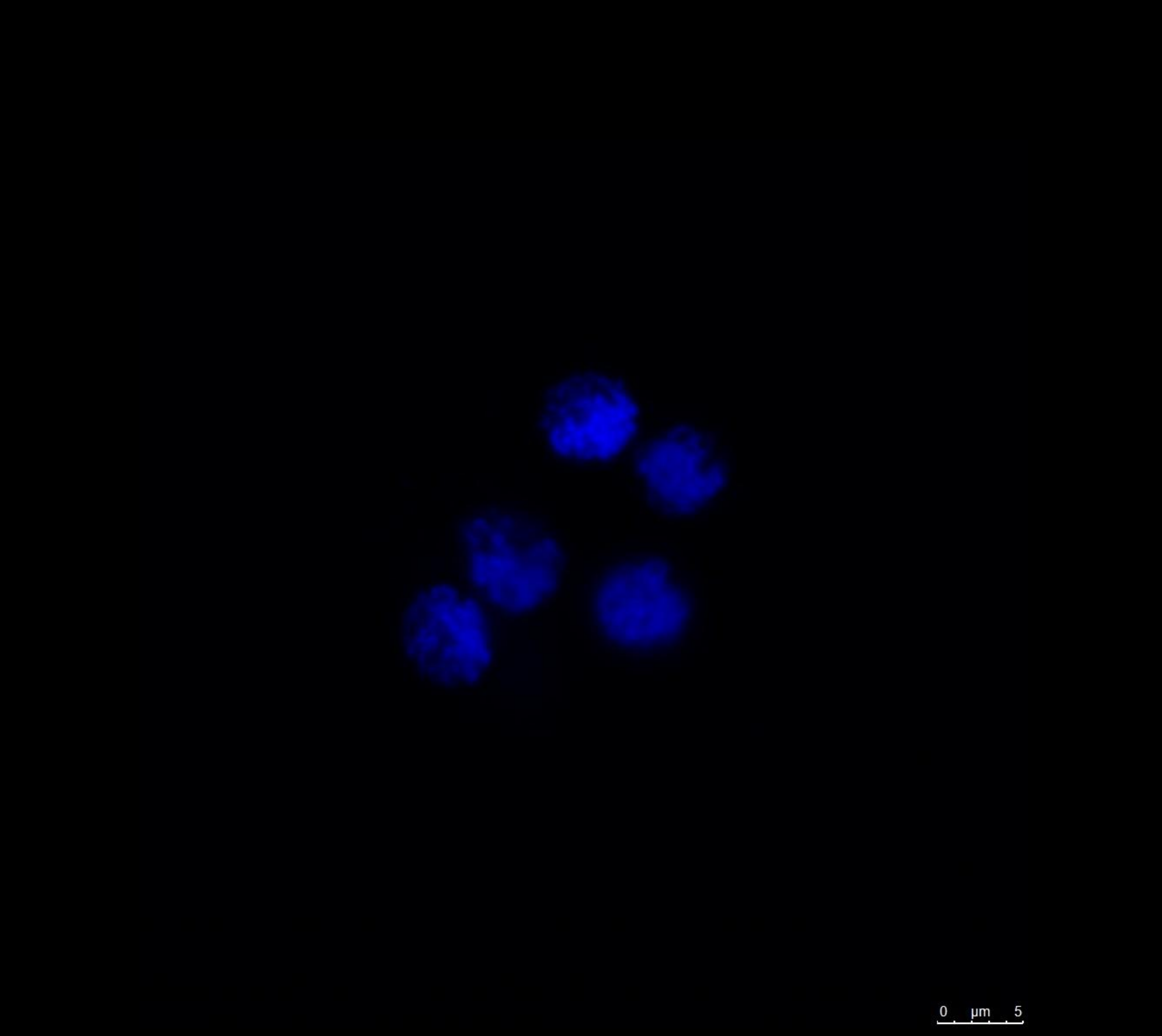

“Multinucleate juvenile” in Fig. 2

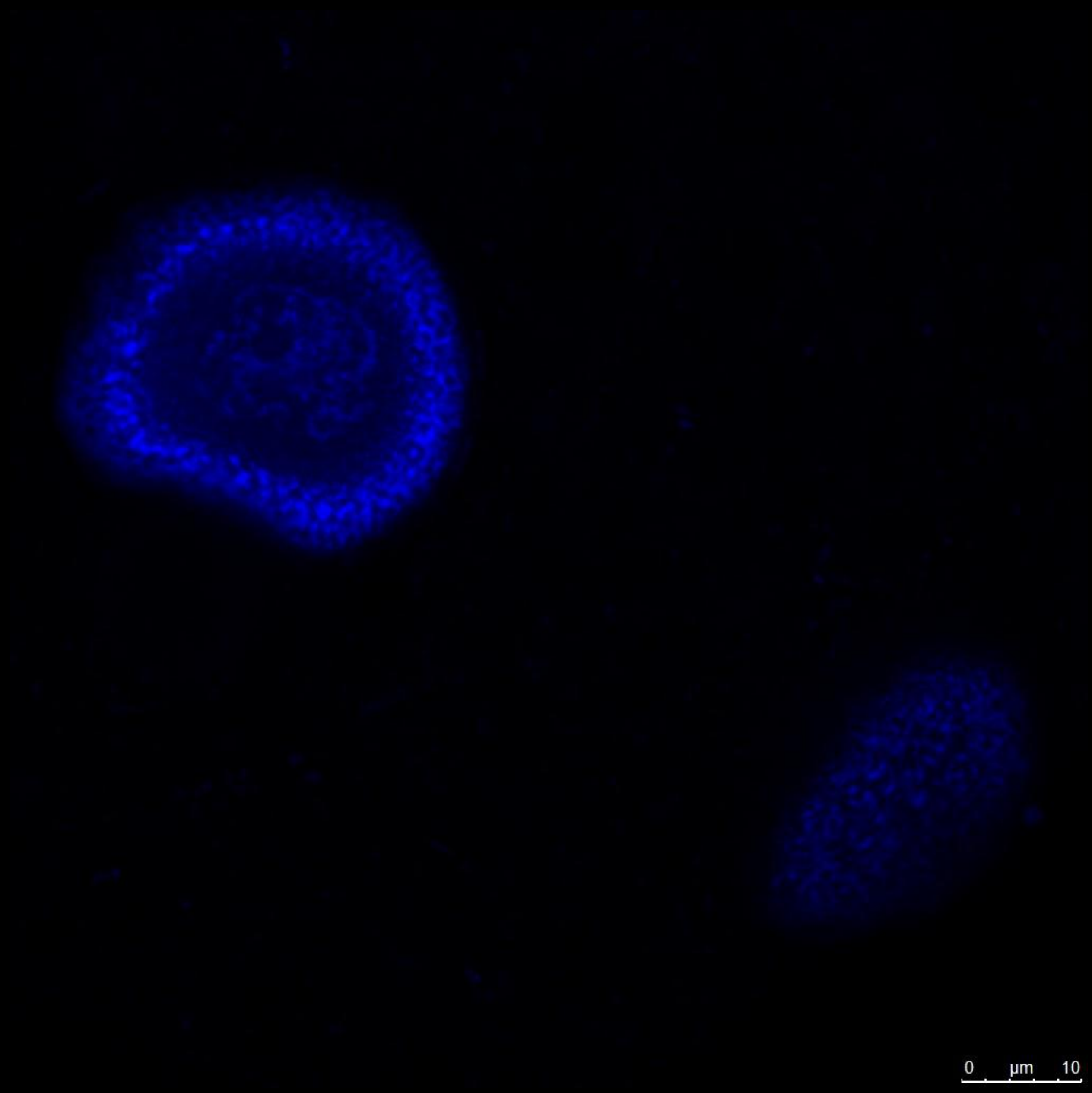

“Multinucleate adult” in Fig. 2

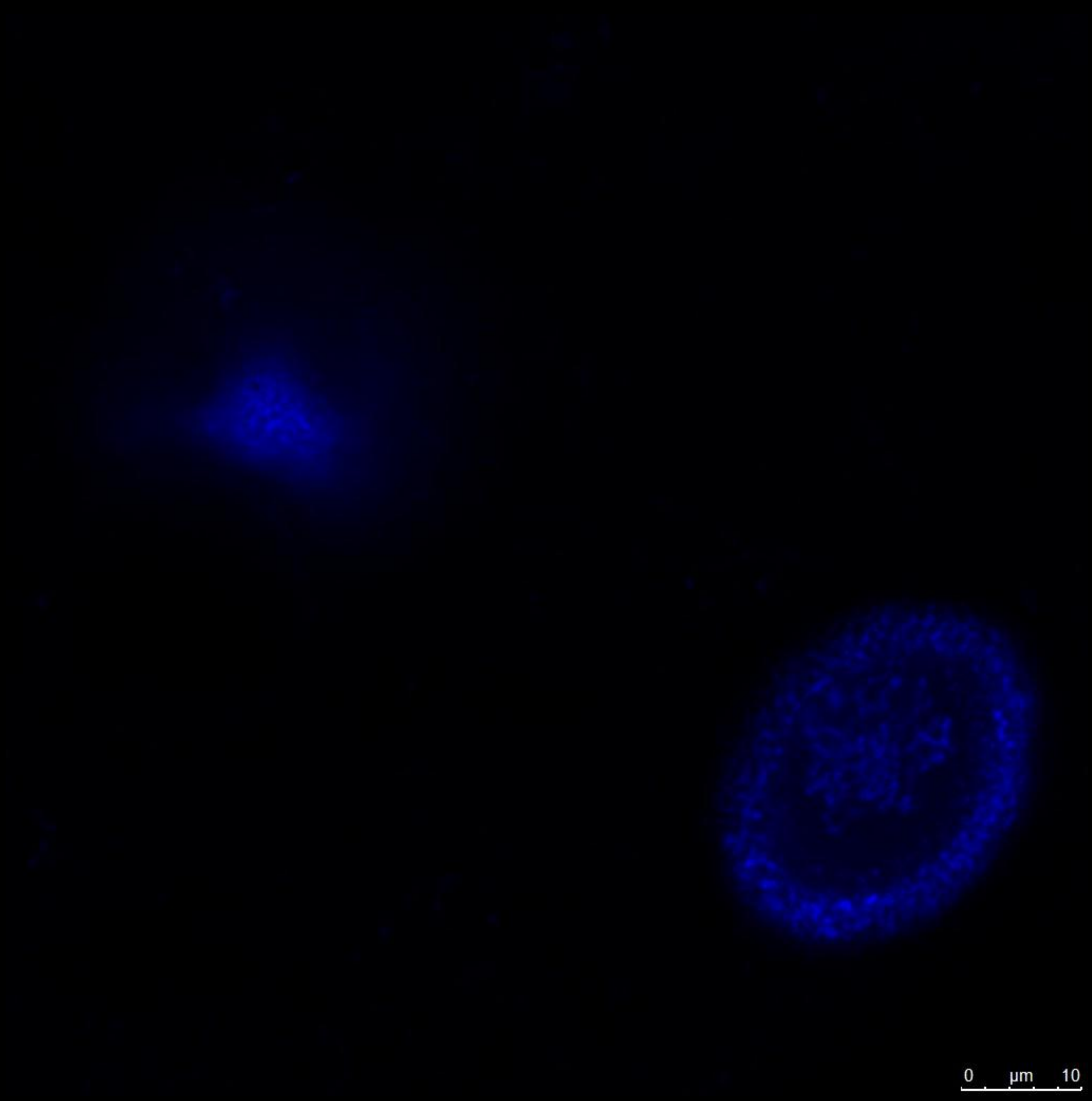

"Multinucleate adult" in Fig. 2

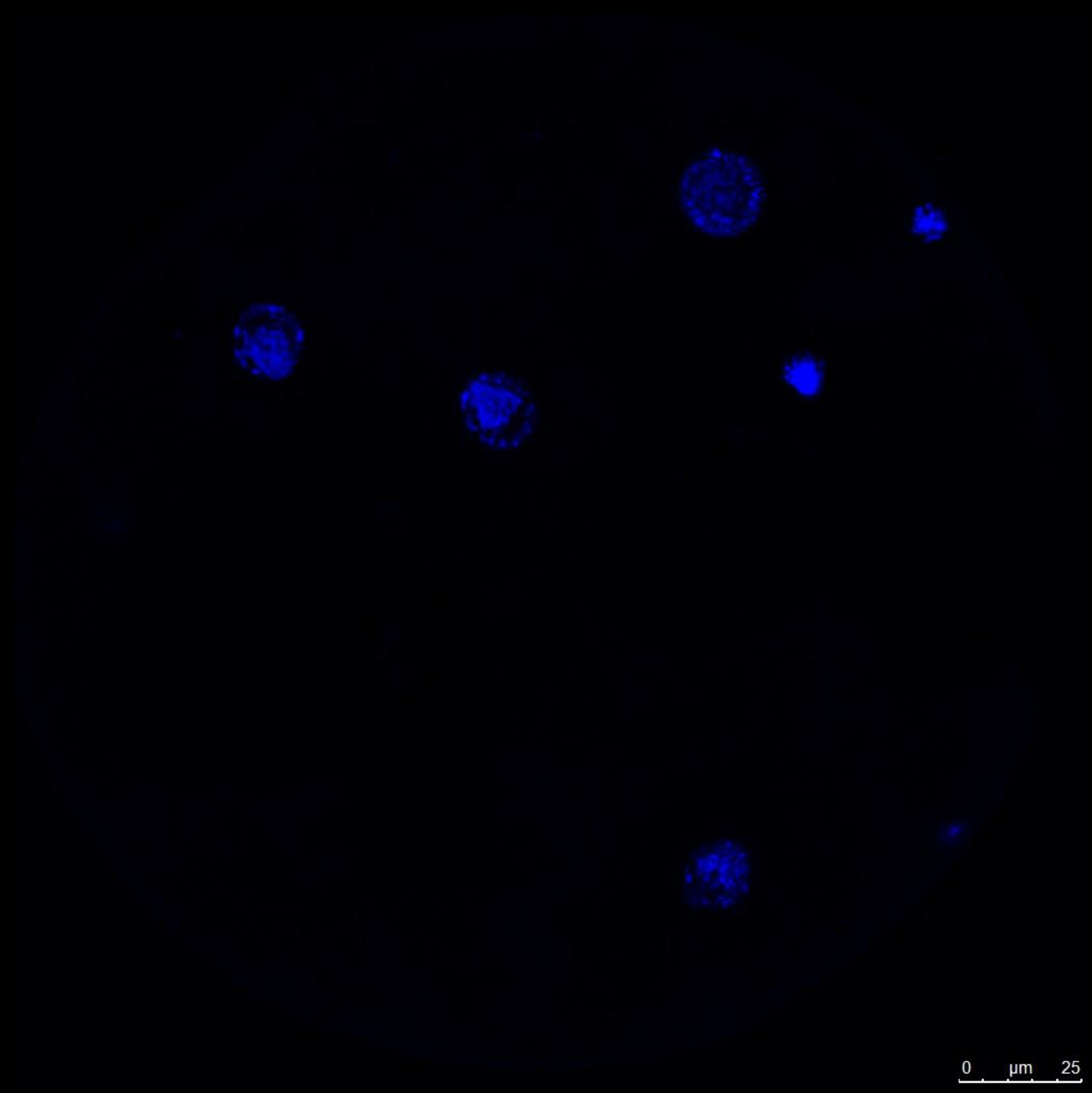

Cell in meiosis in Fig. 2

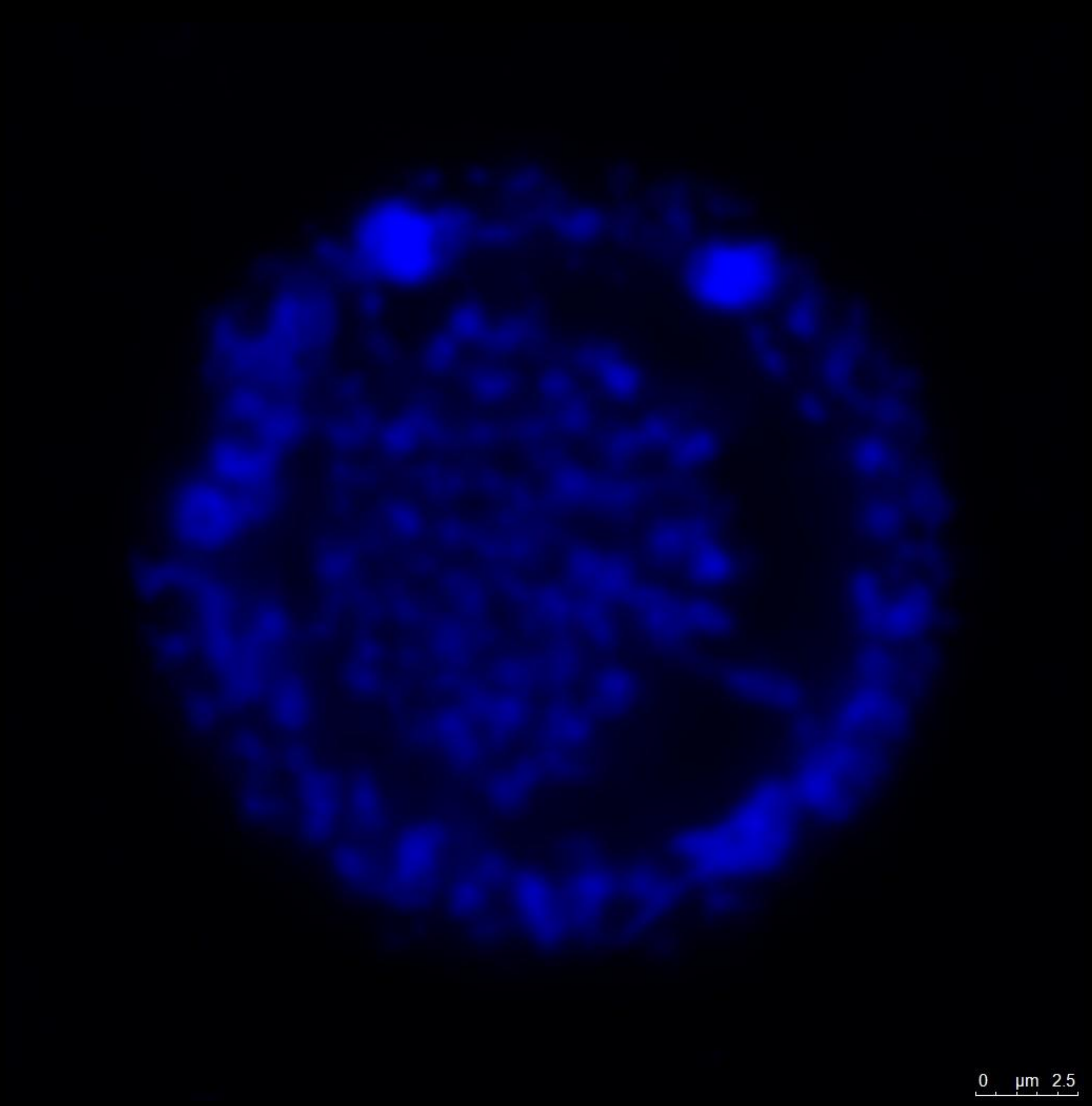

“Nuclei in meiosis” in Fig. 2

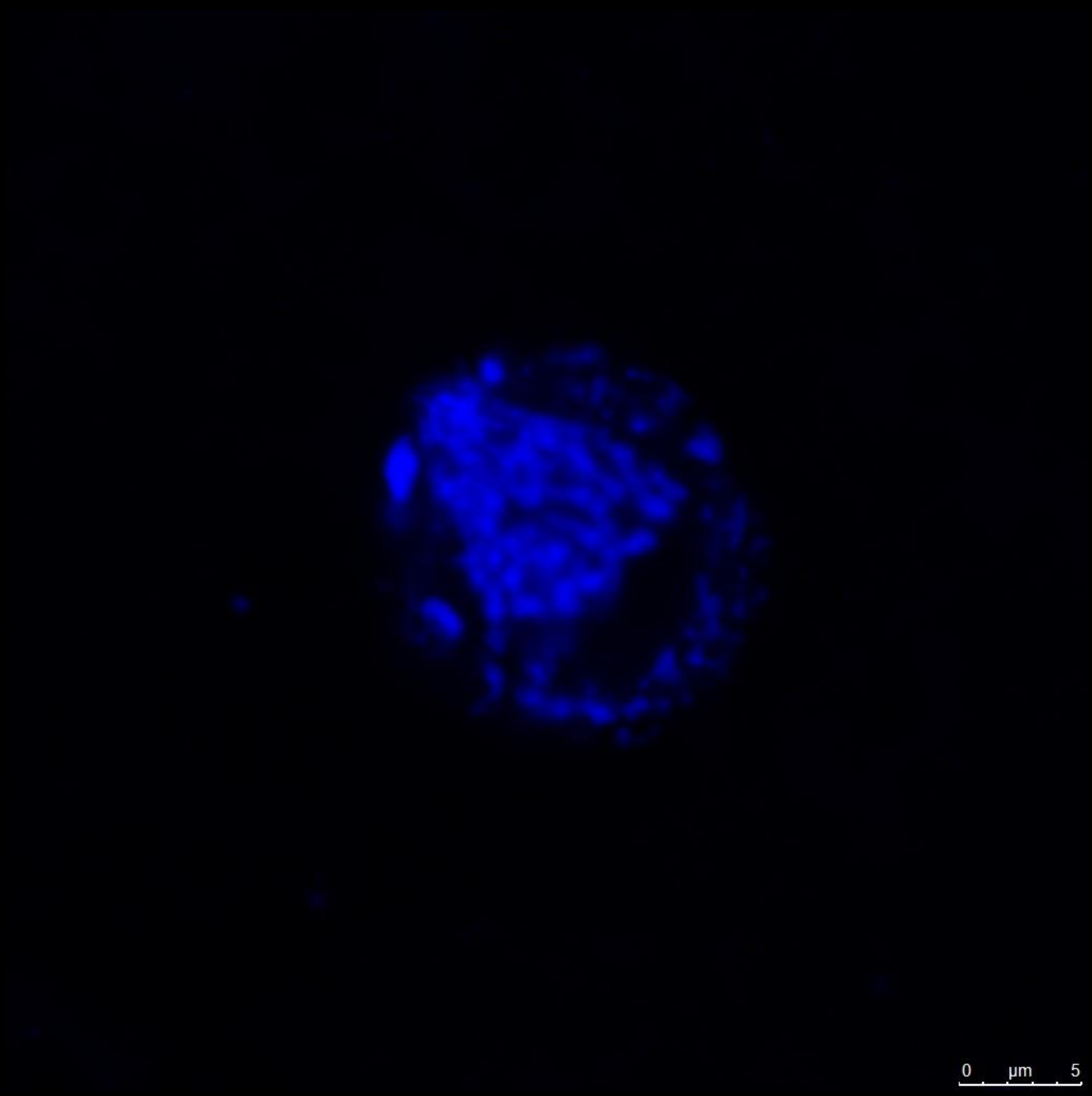

“Nuclei in meiosis” in Fig. 2

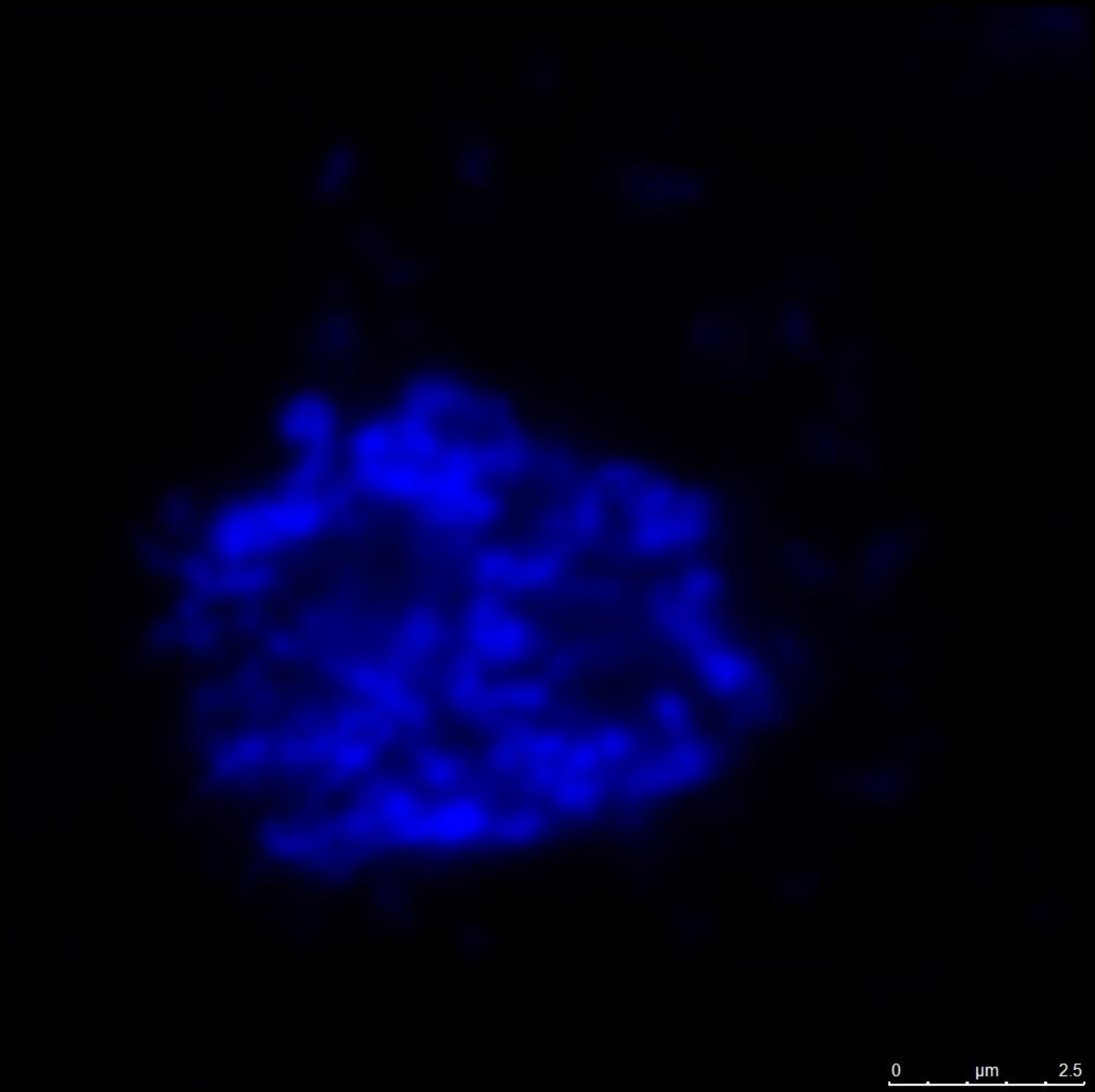

“Nuclei in meiosis” in Fig. 2

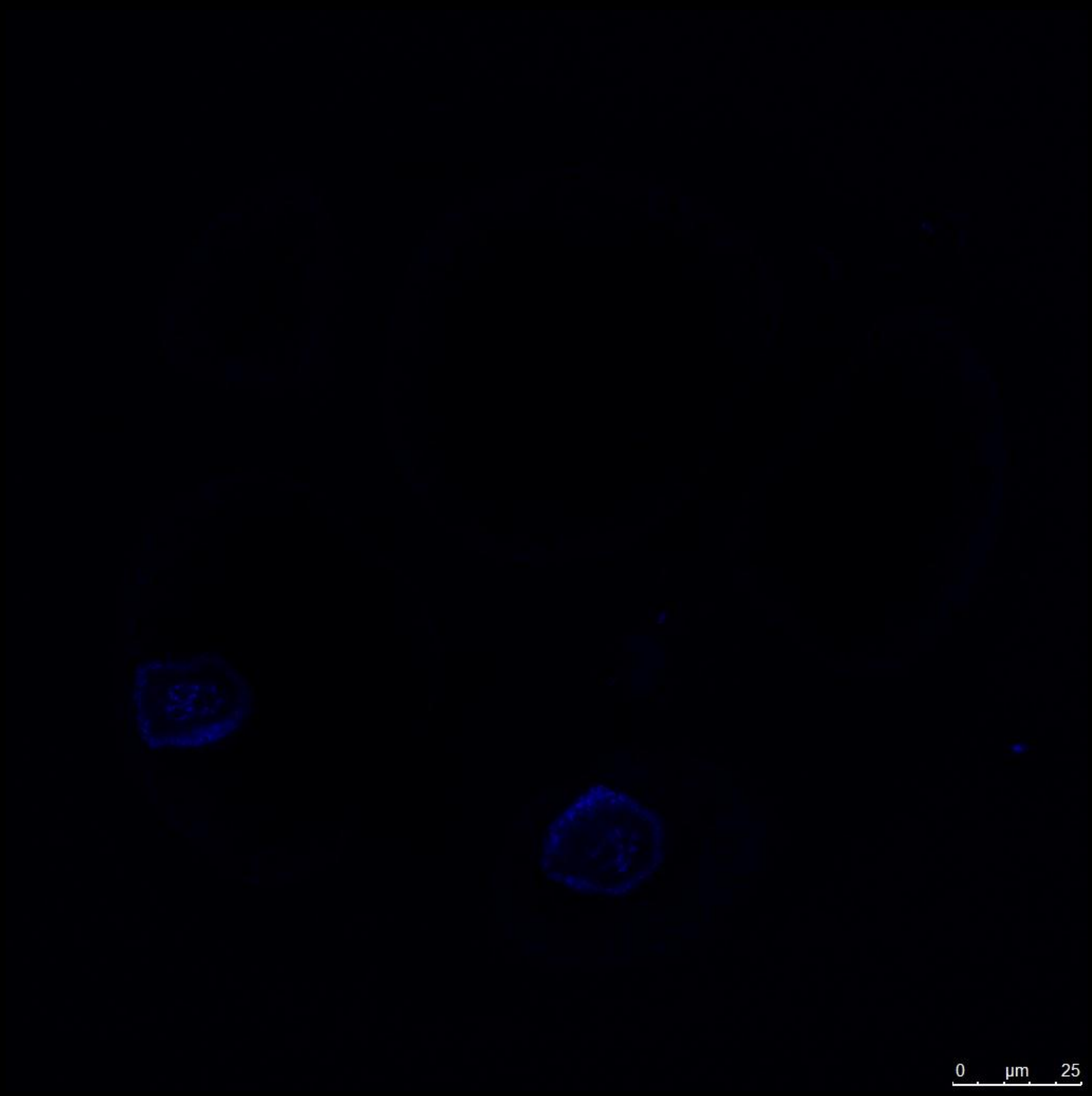

“Uninucleate juvenile (in parent test)” in Fig. 2

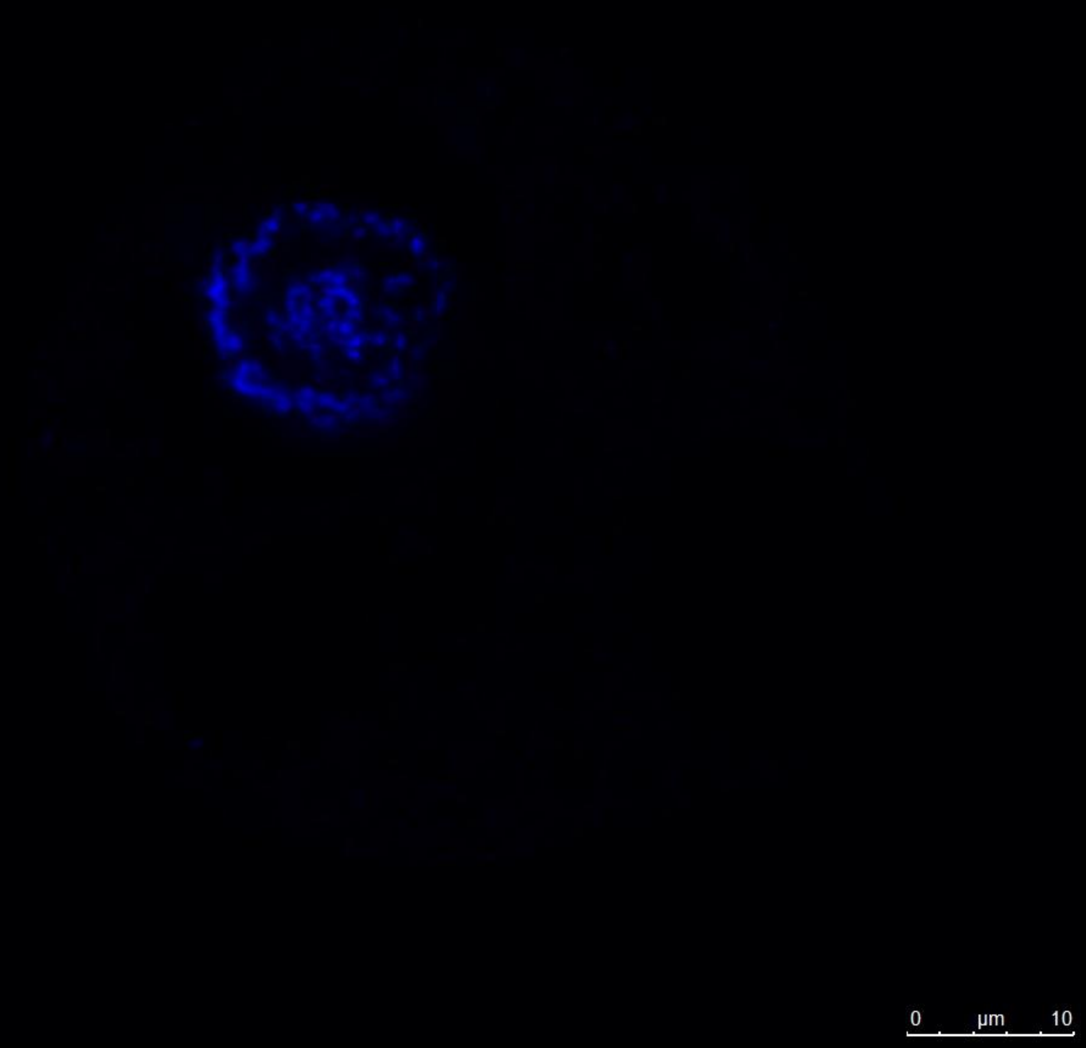

“Uninucleate juvenile” in Fig. 2

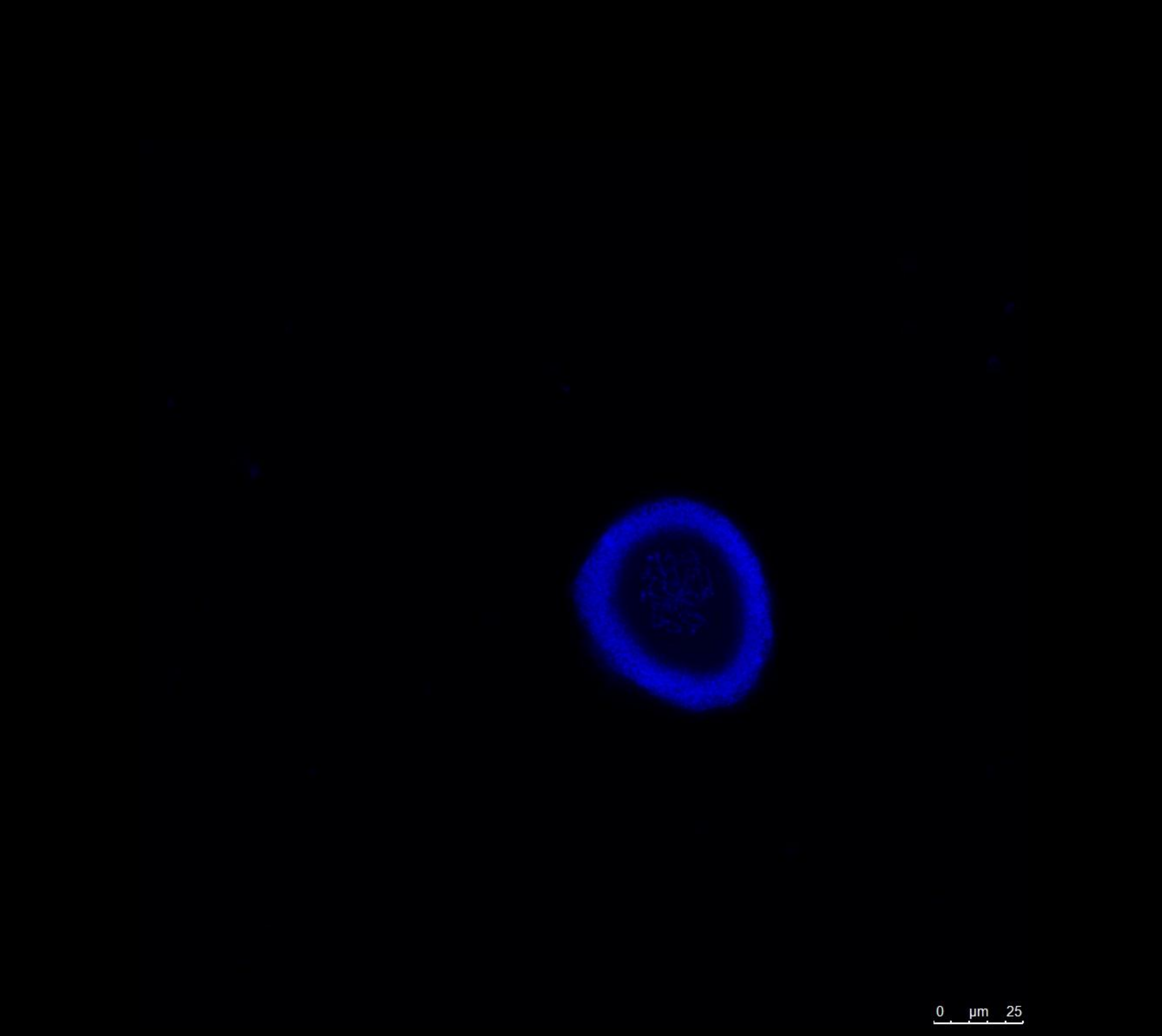

“Uninucleate adult” in Fig. 2

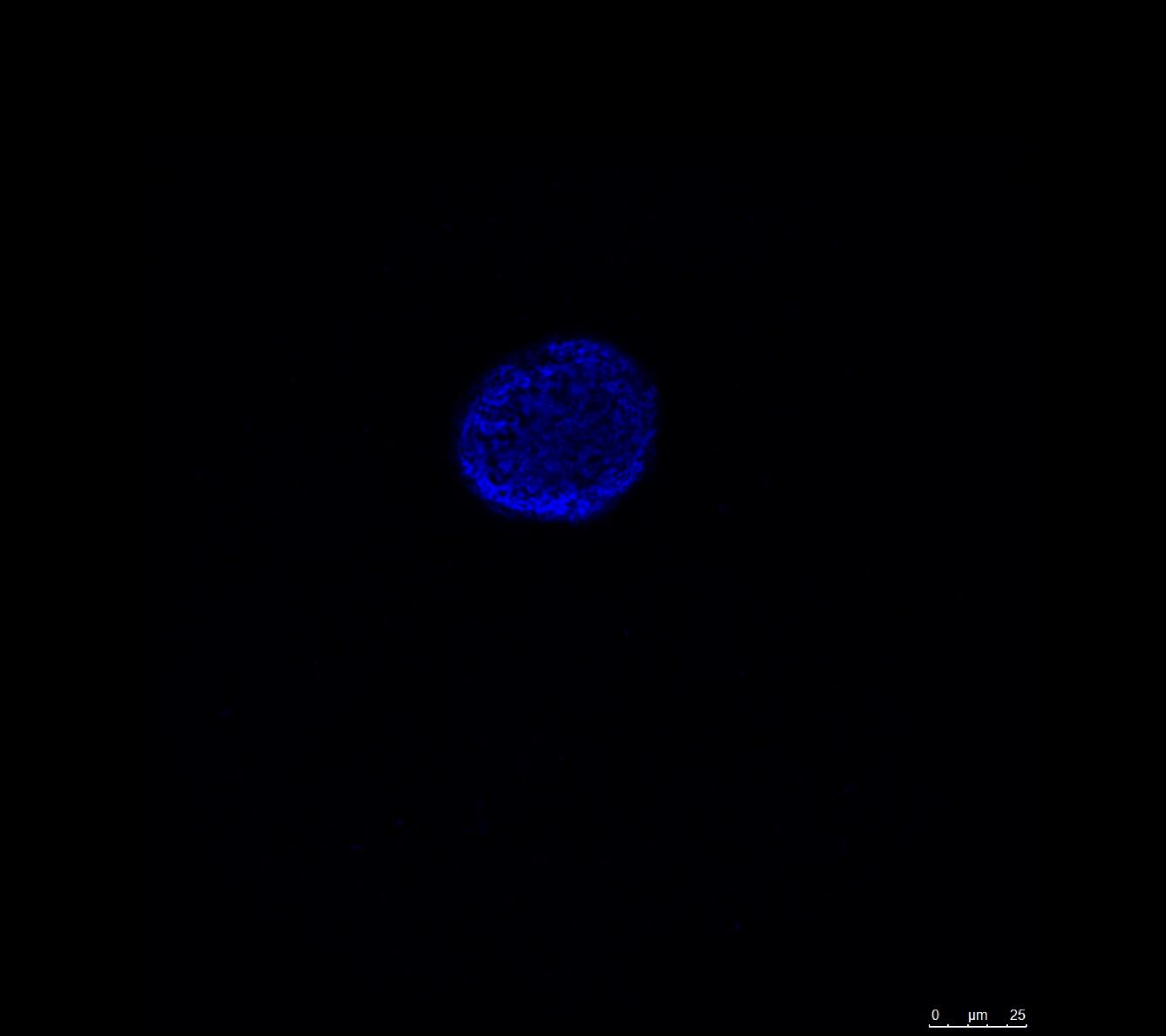

First cell in *Zerfall* panel; Fig. 2 and Fig. S4A

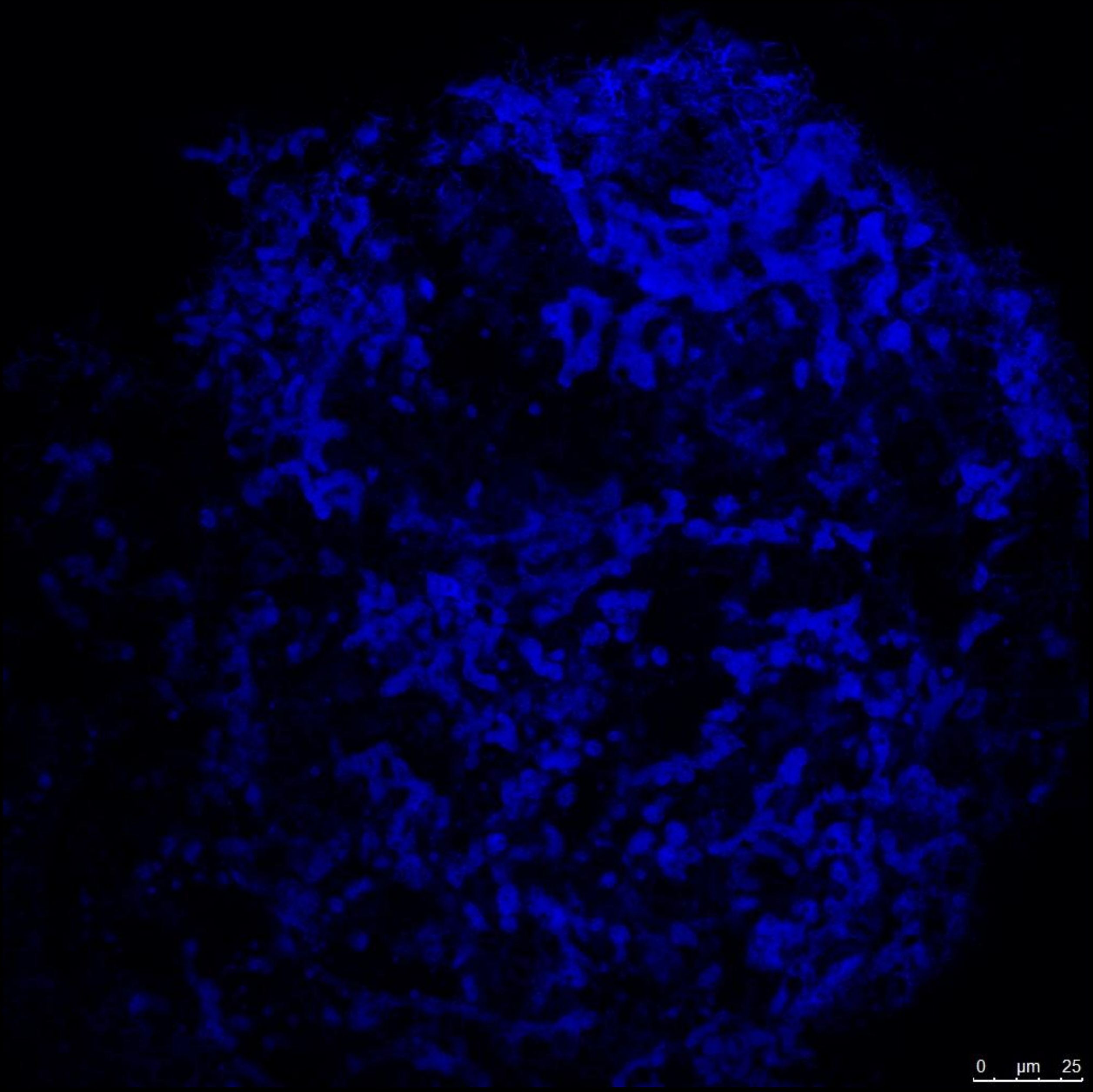

Second cell in *Zerfall* panel; Fig. S4D

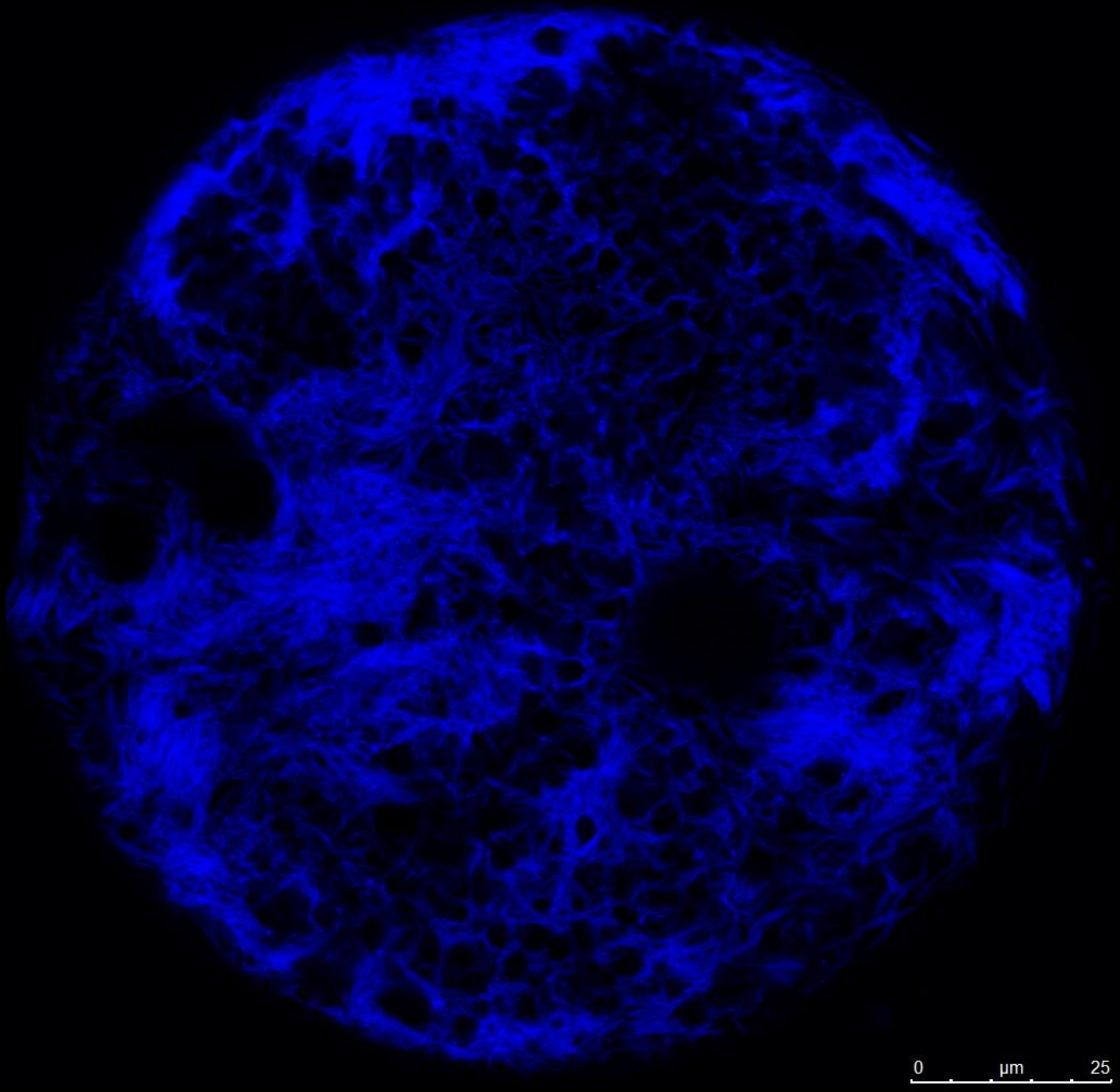

Third cell in Zerfall panel in Fig. 2; Fig. S4H

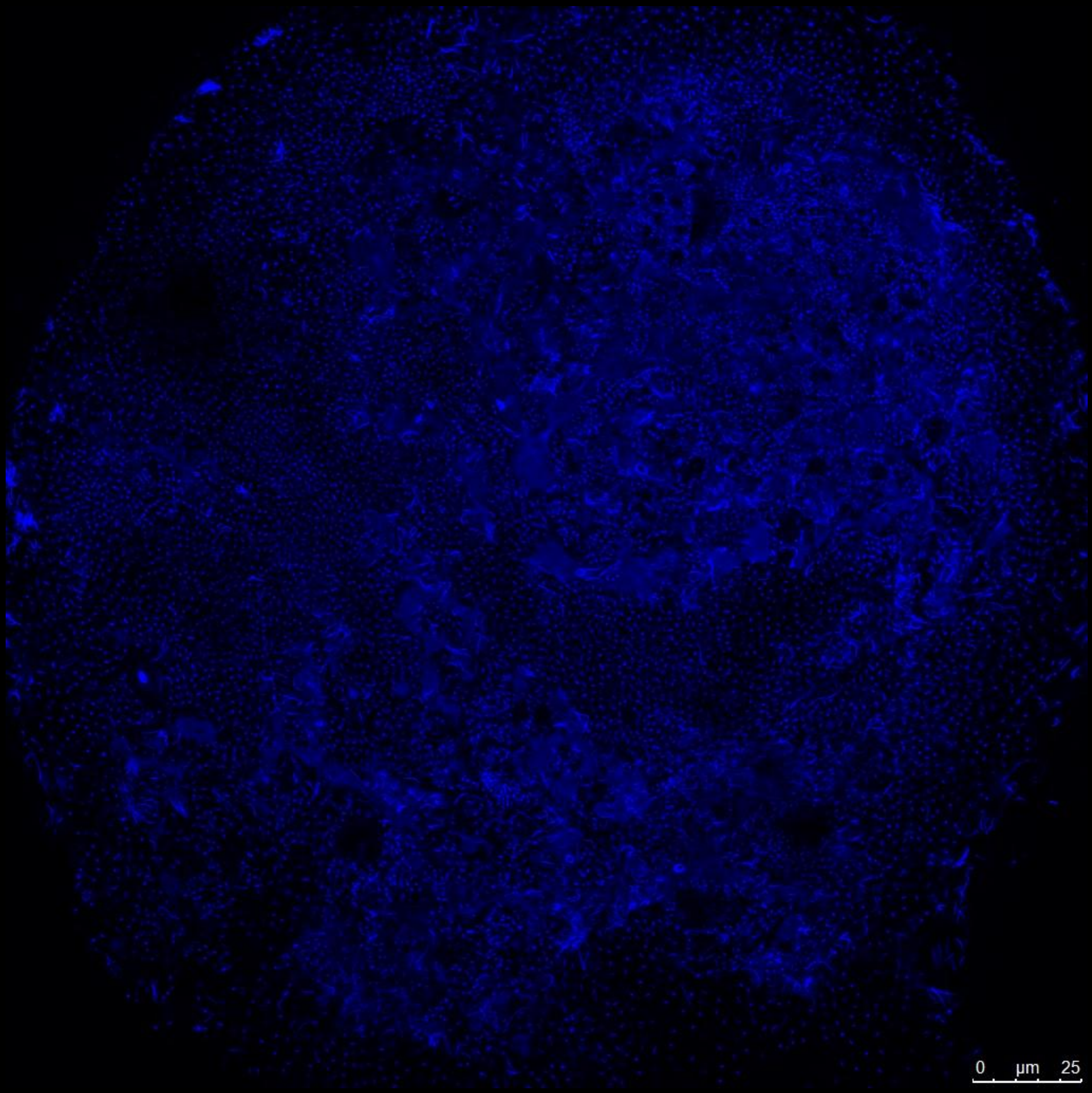

Fourth cell in *Zerfall* panel in Fig. 2; Fig. S4O

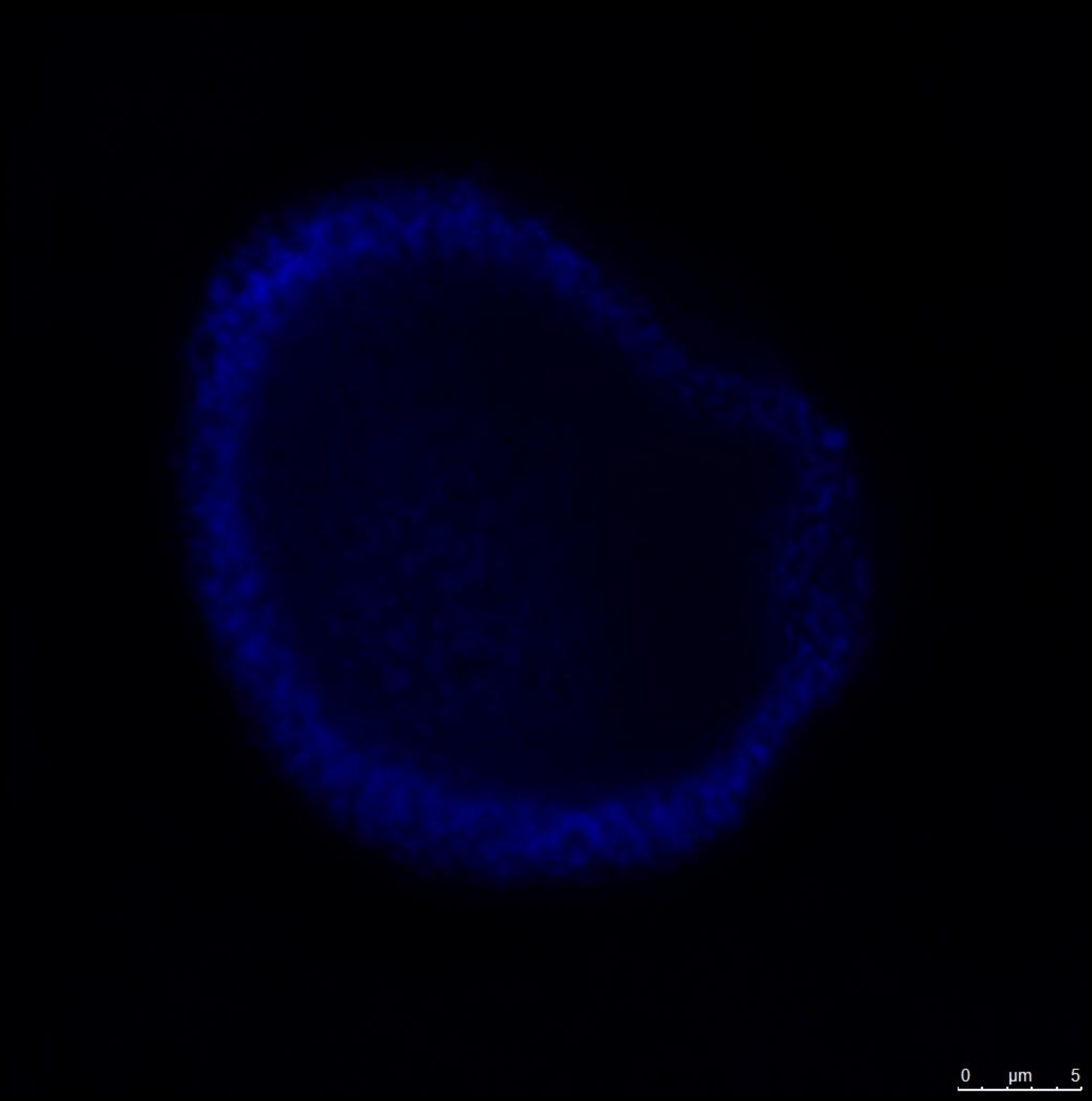

Fig. 3A

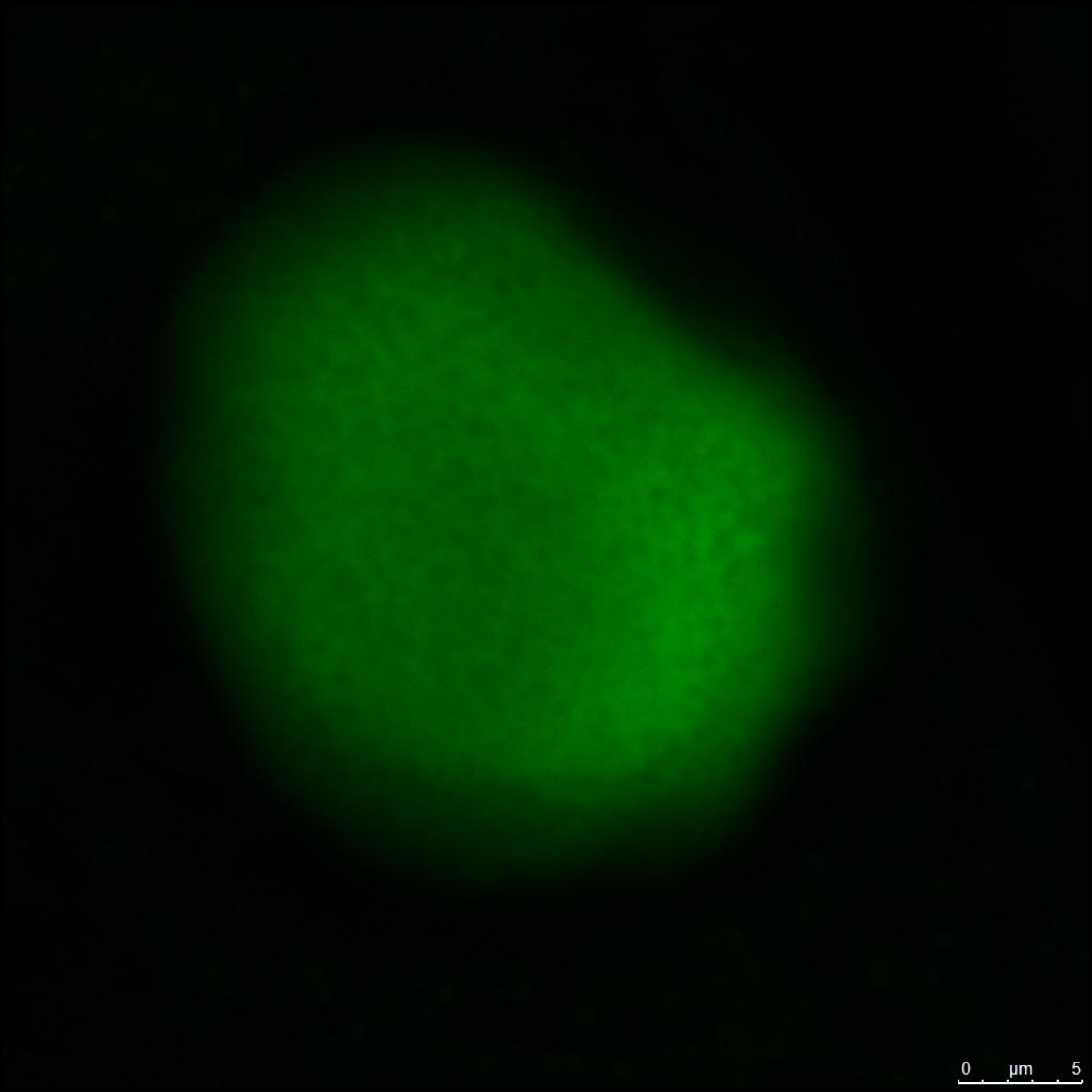

Fig. 3B

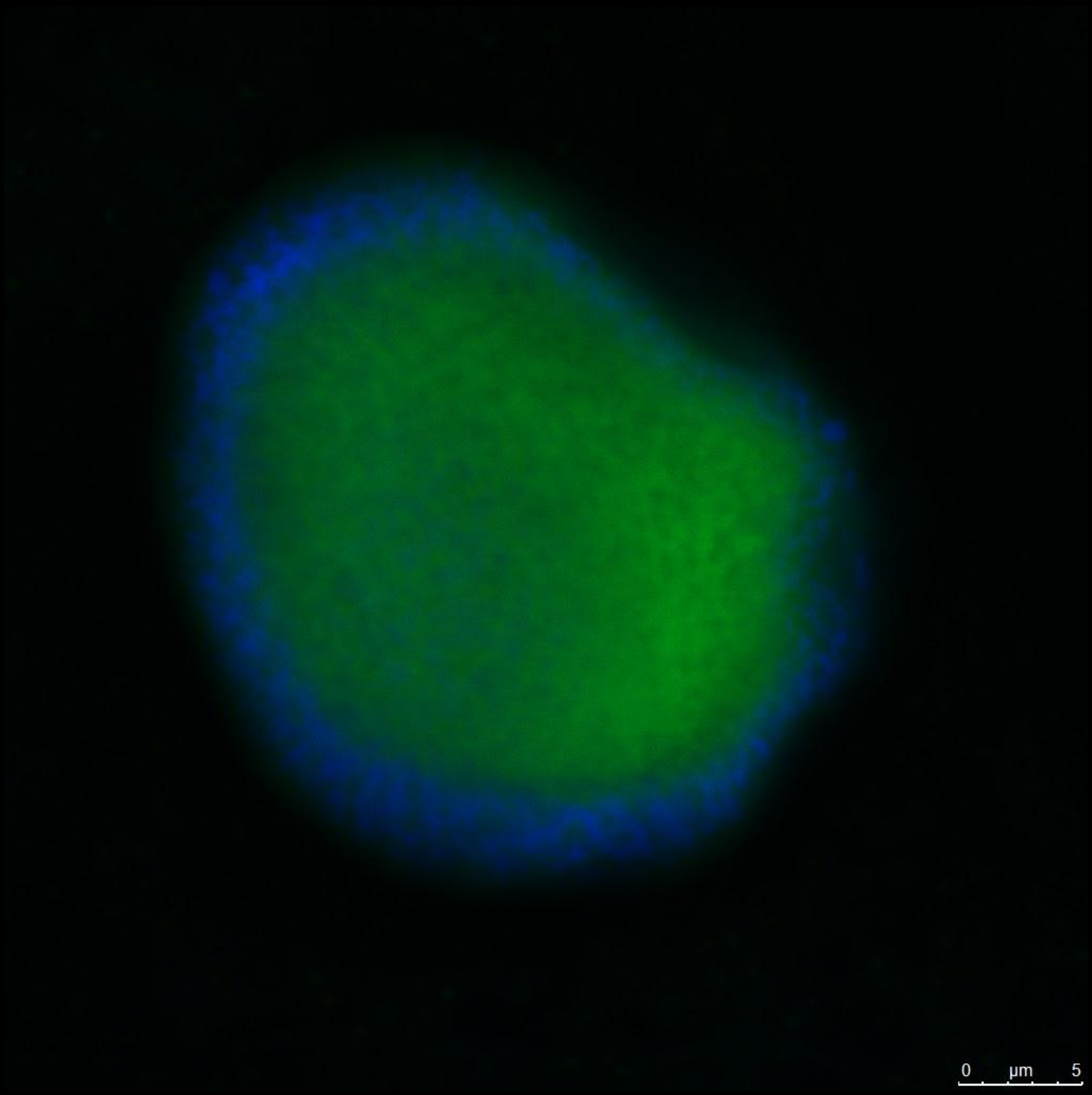

Fig. 3C

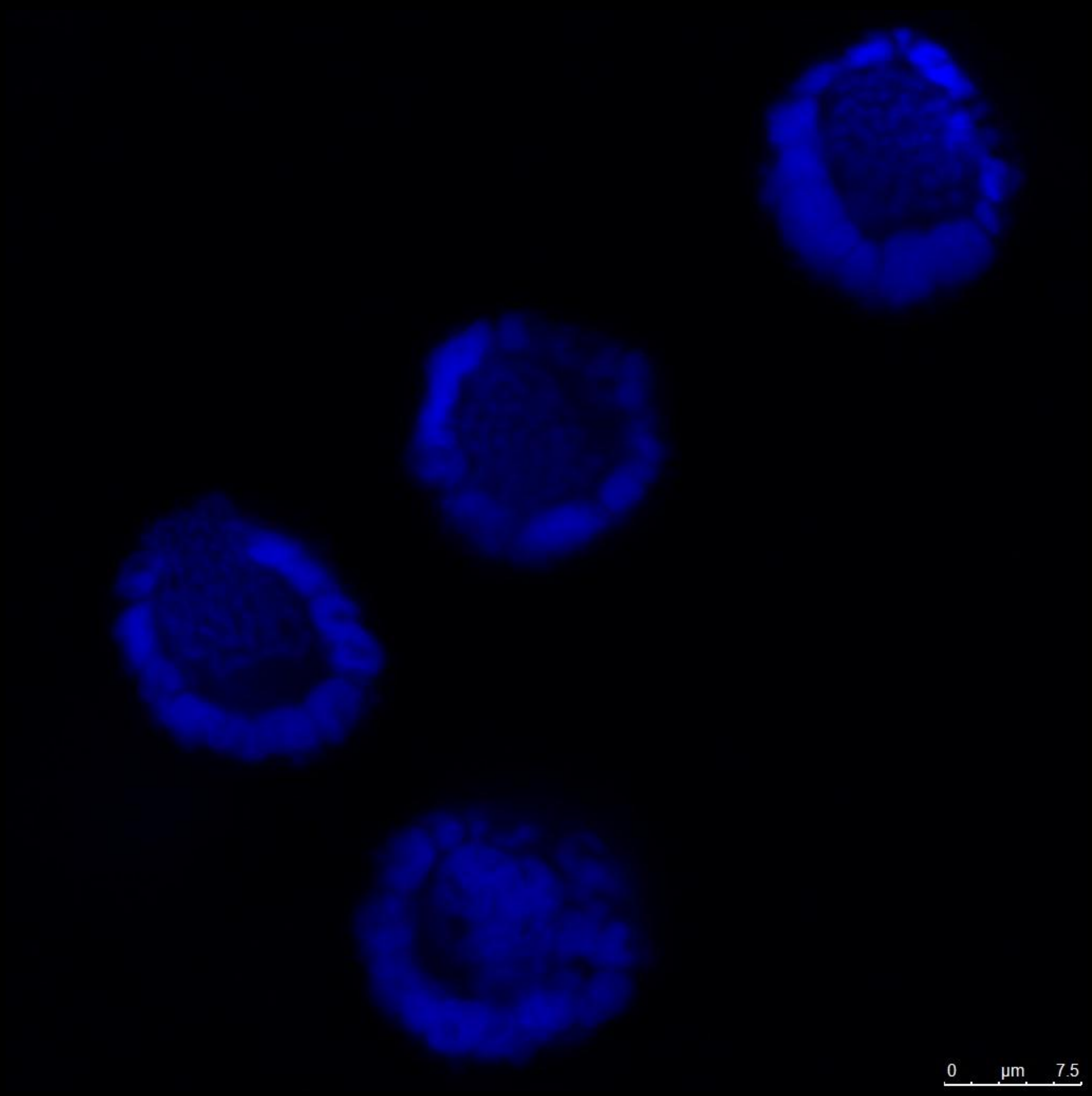

Fig. 3D

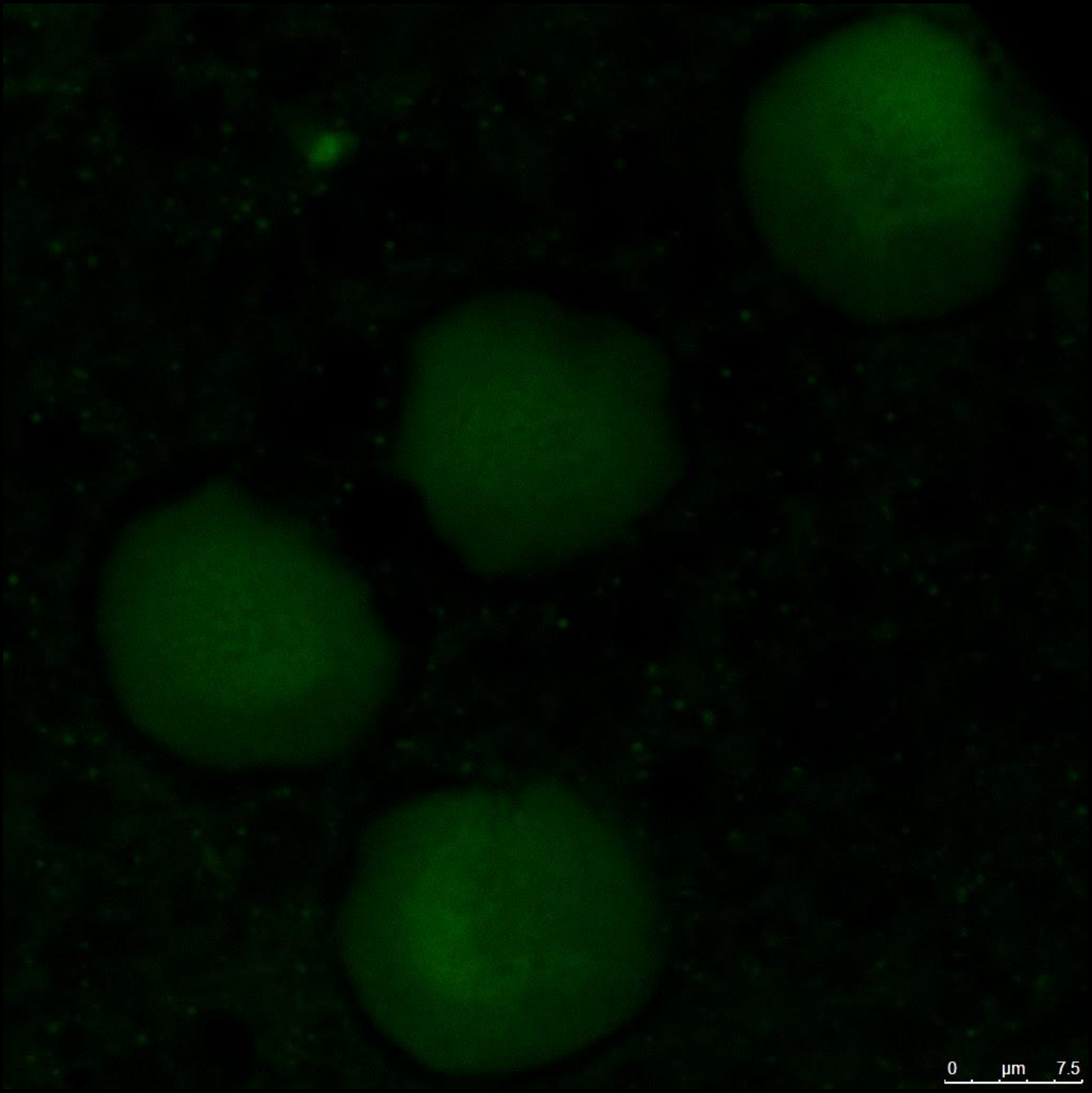

Fig. 3E

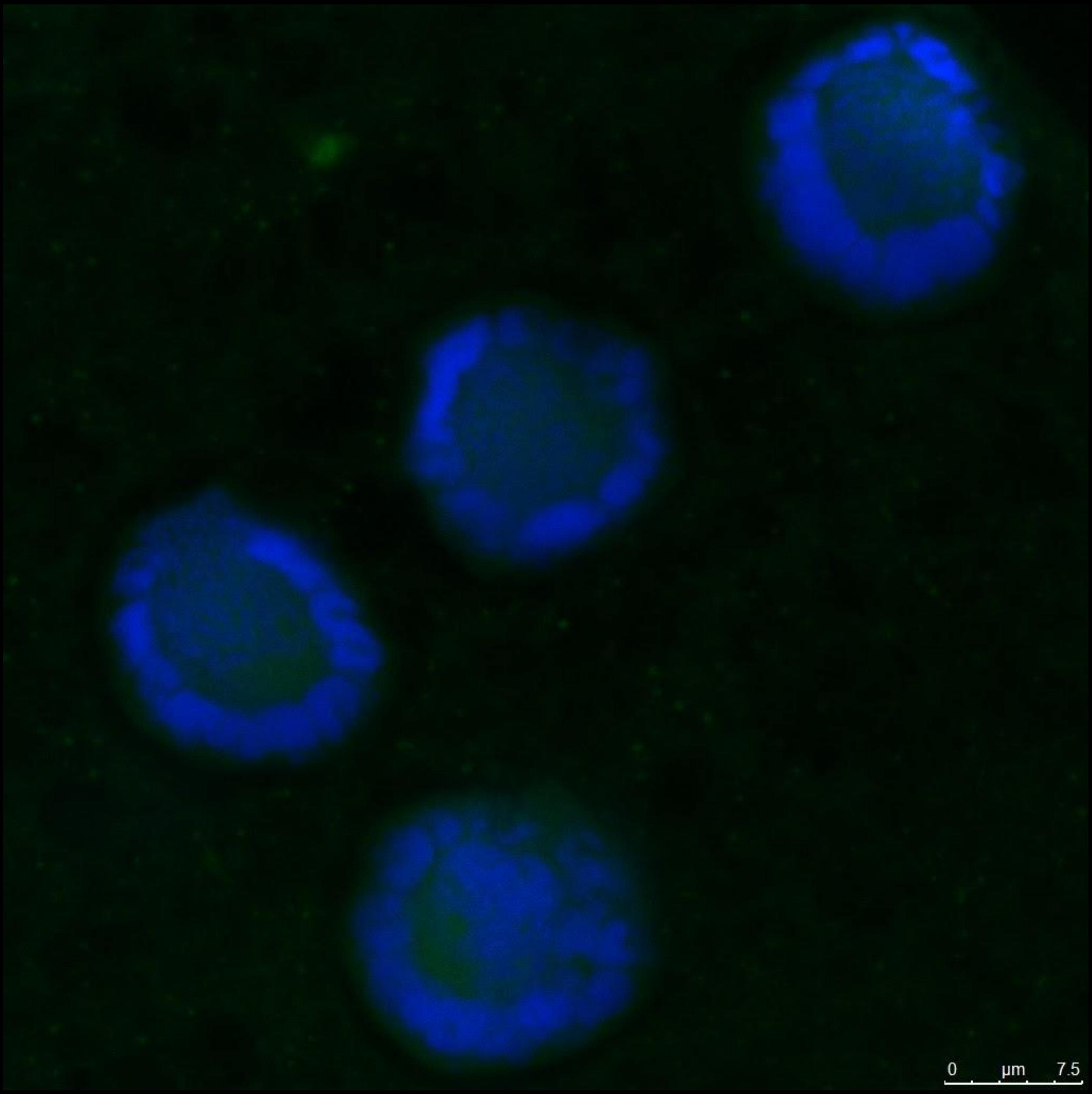

Fig. 3F

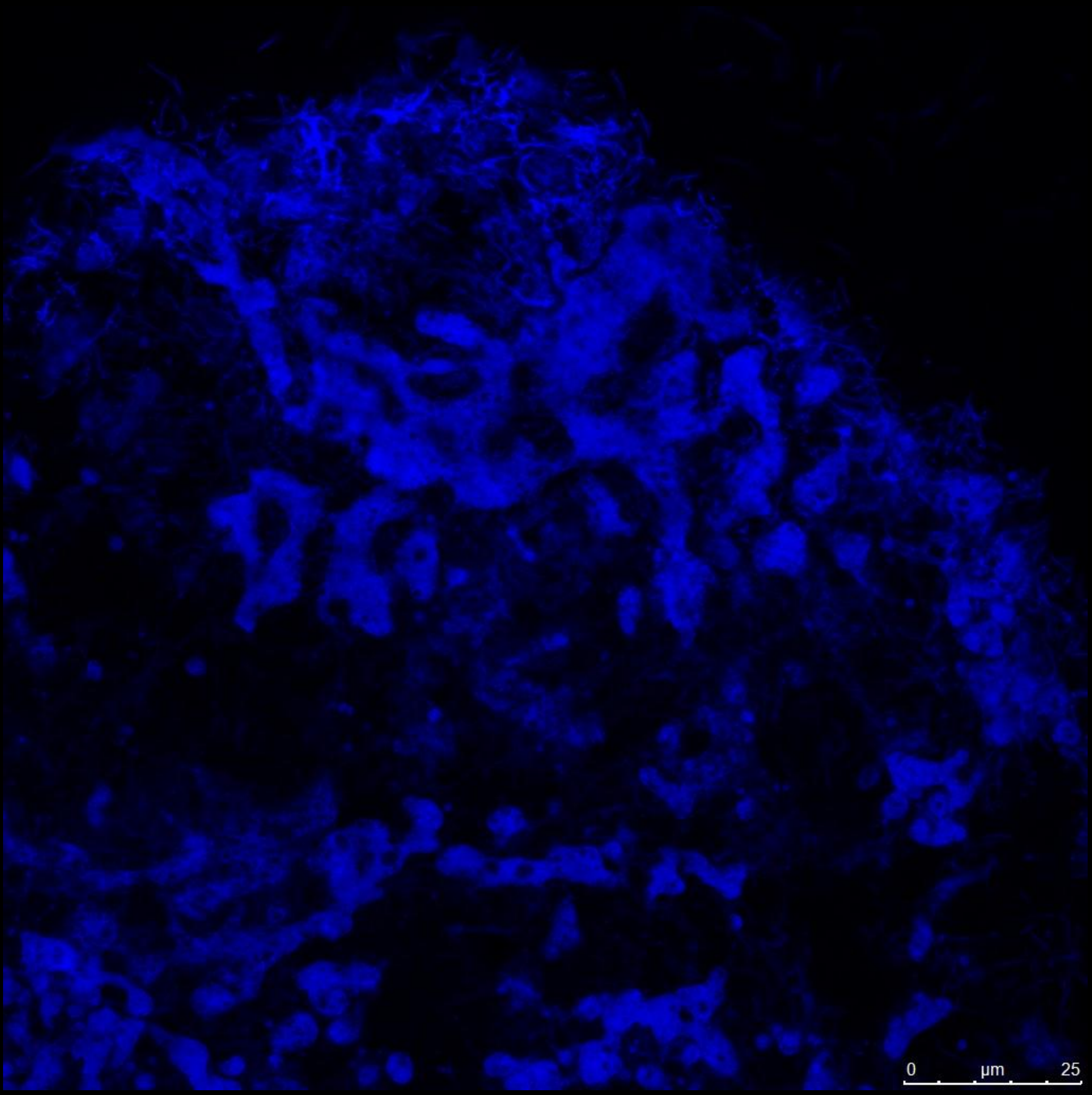

Fig. 4A

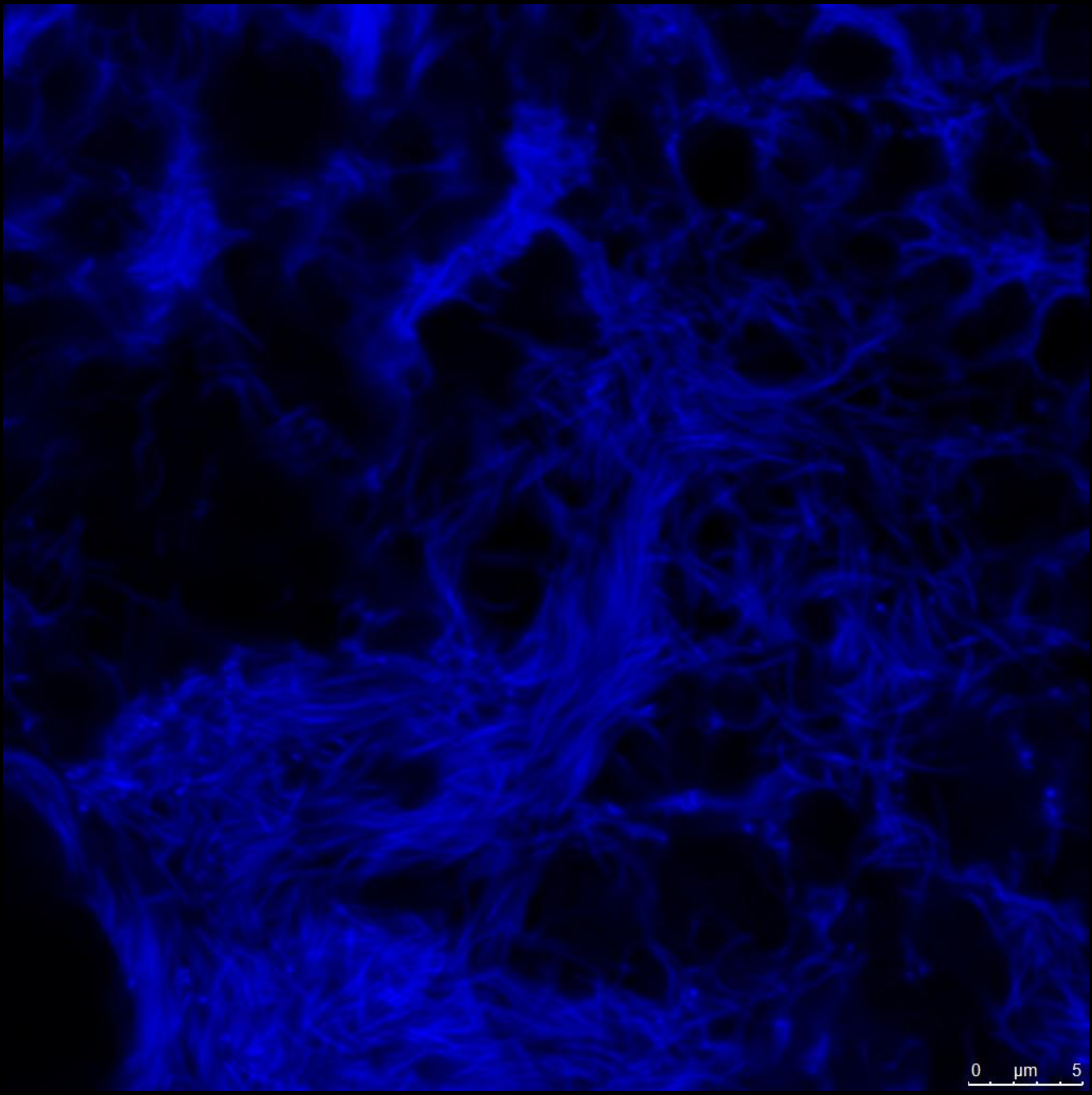

Fig. 4B

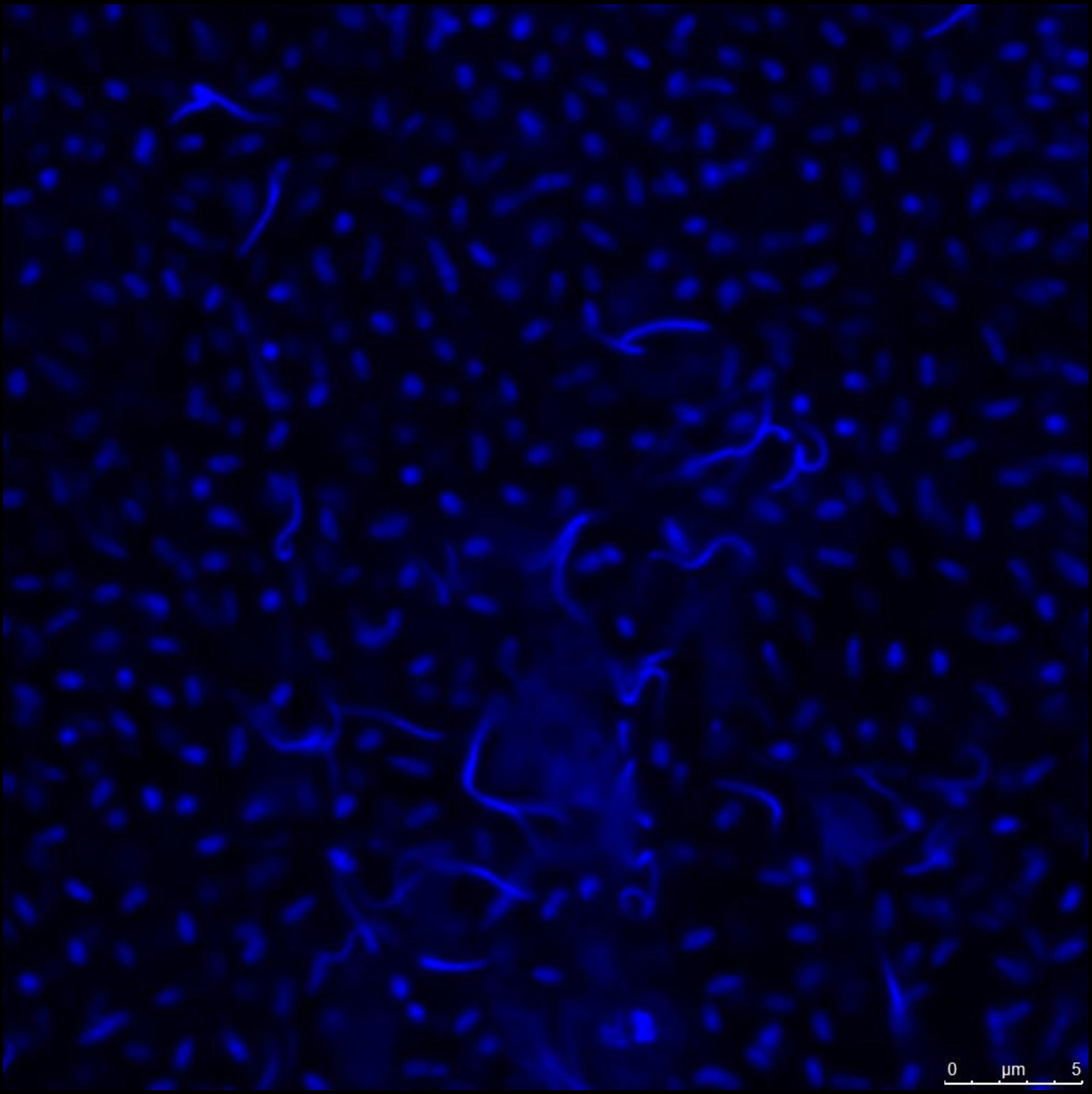

Fig. 4C

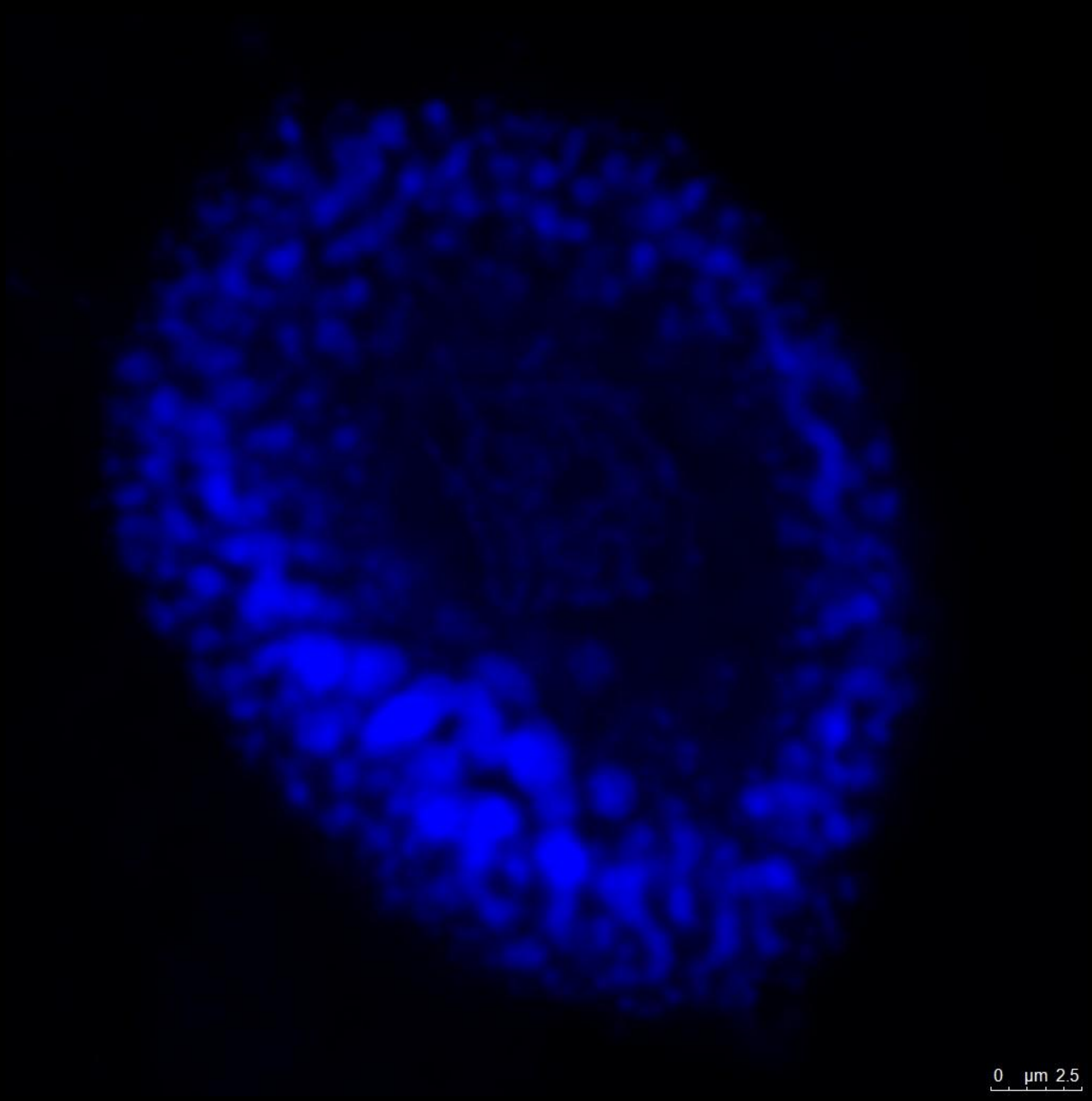

Fig. S2A

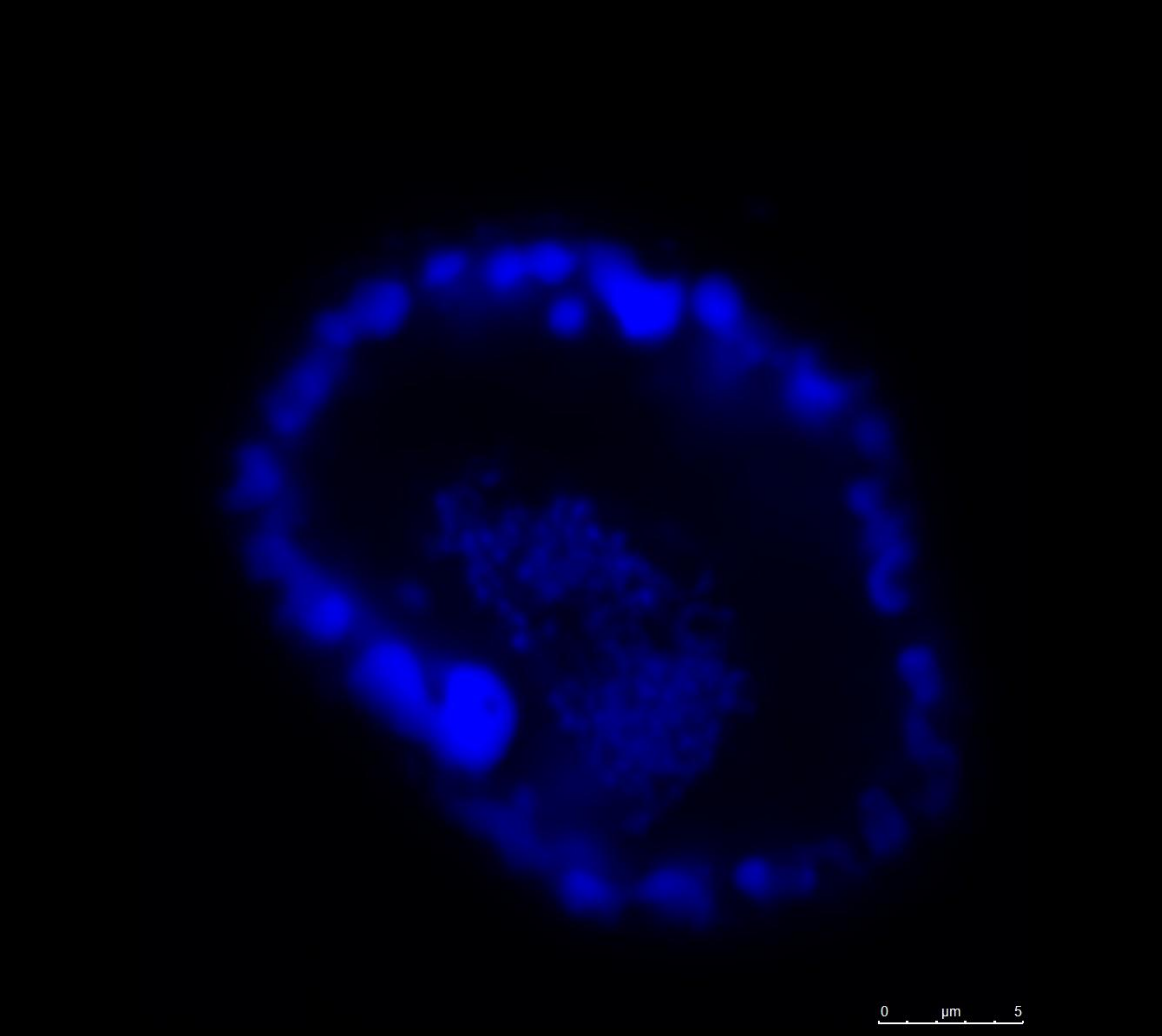

Fig. S2B

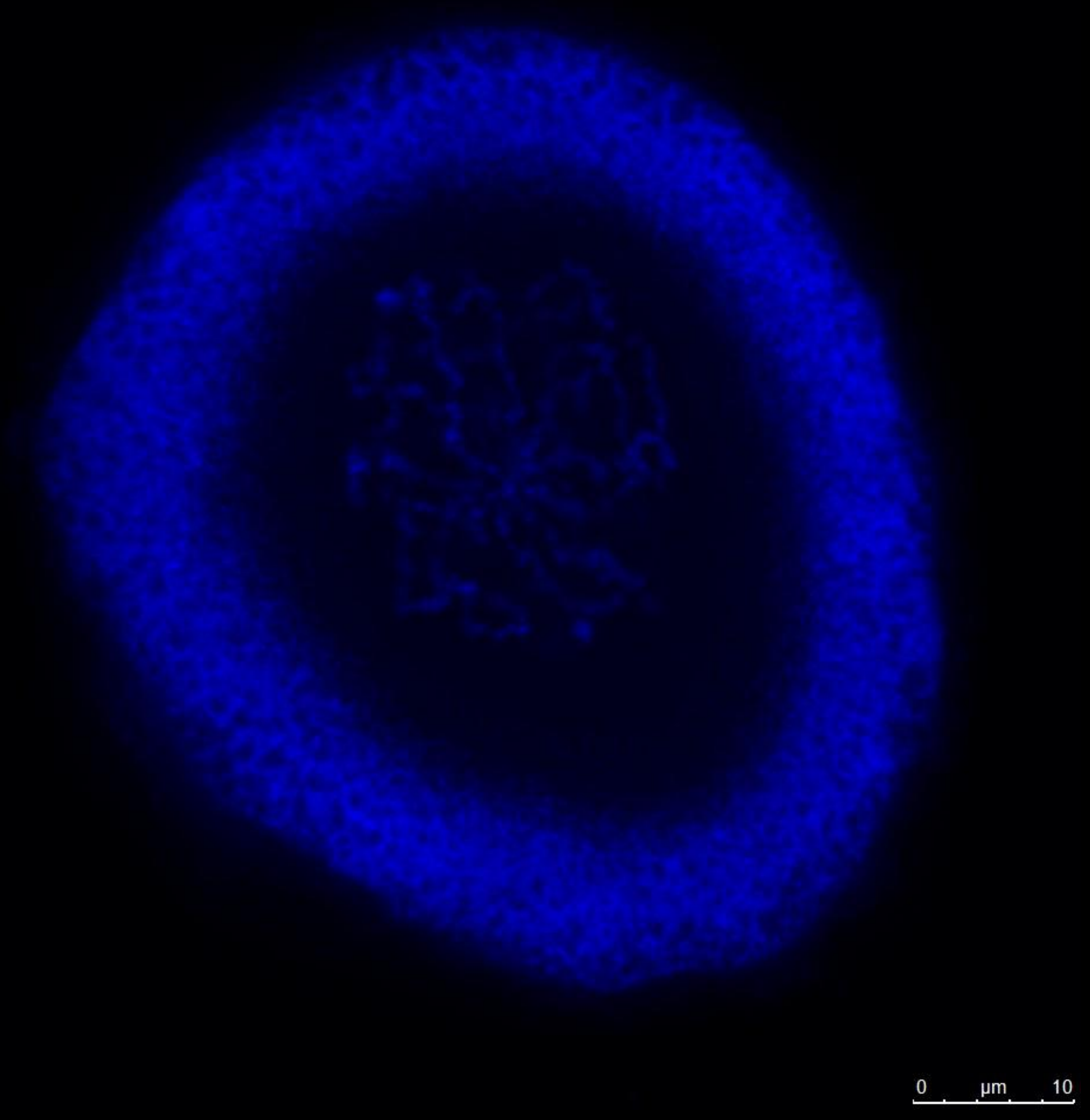

Fig. S2C

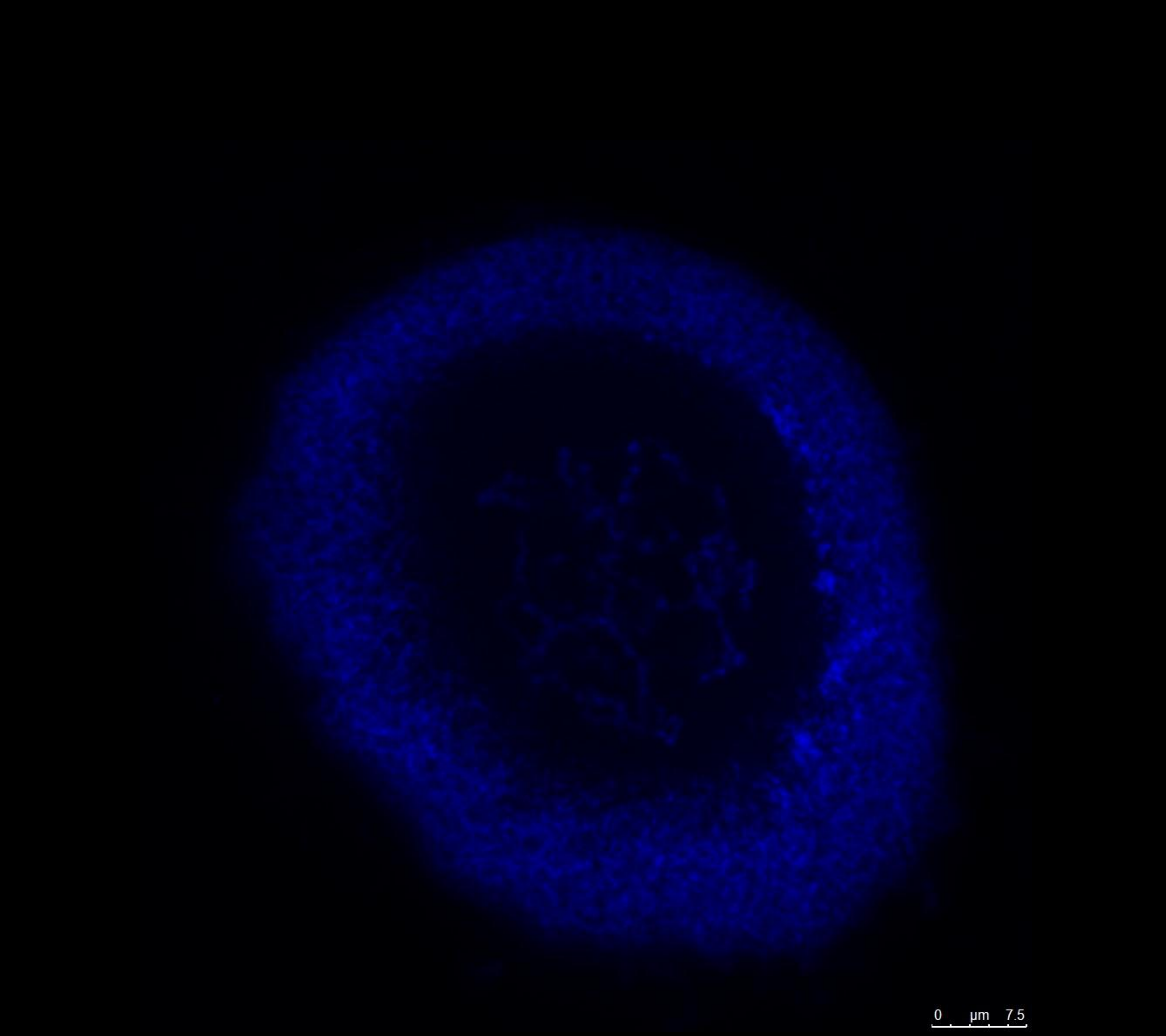

Fig. S2D

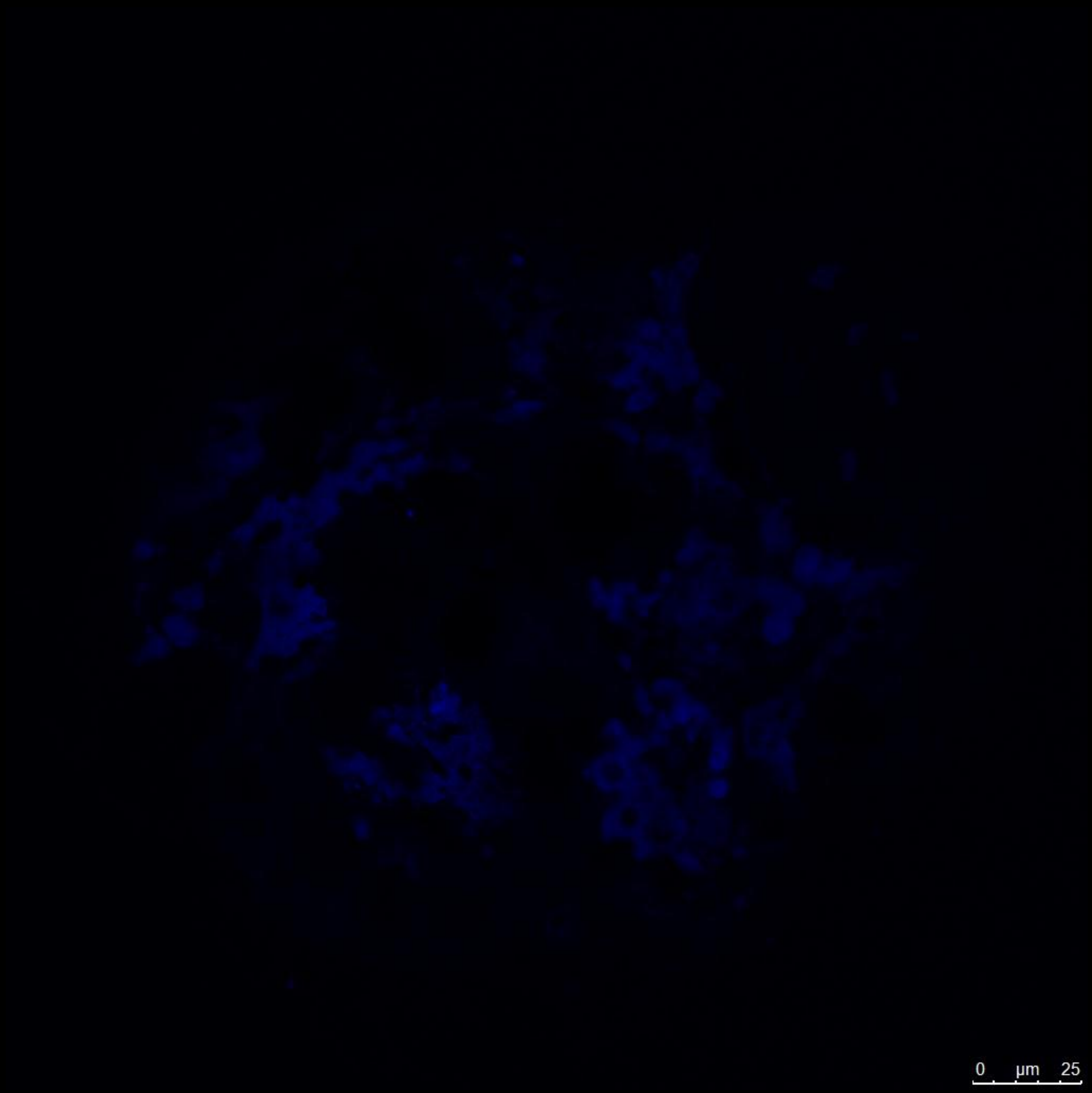

Fig. S4B

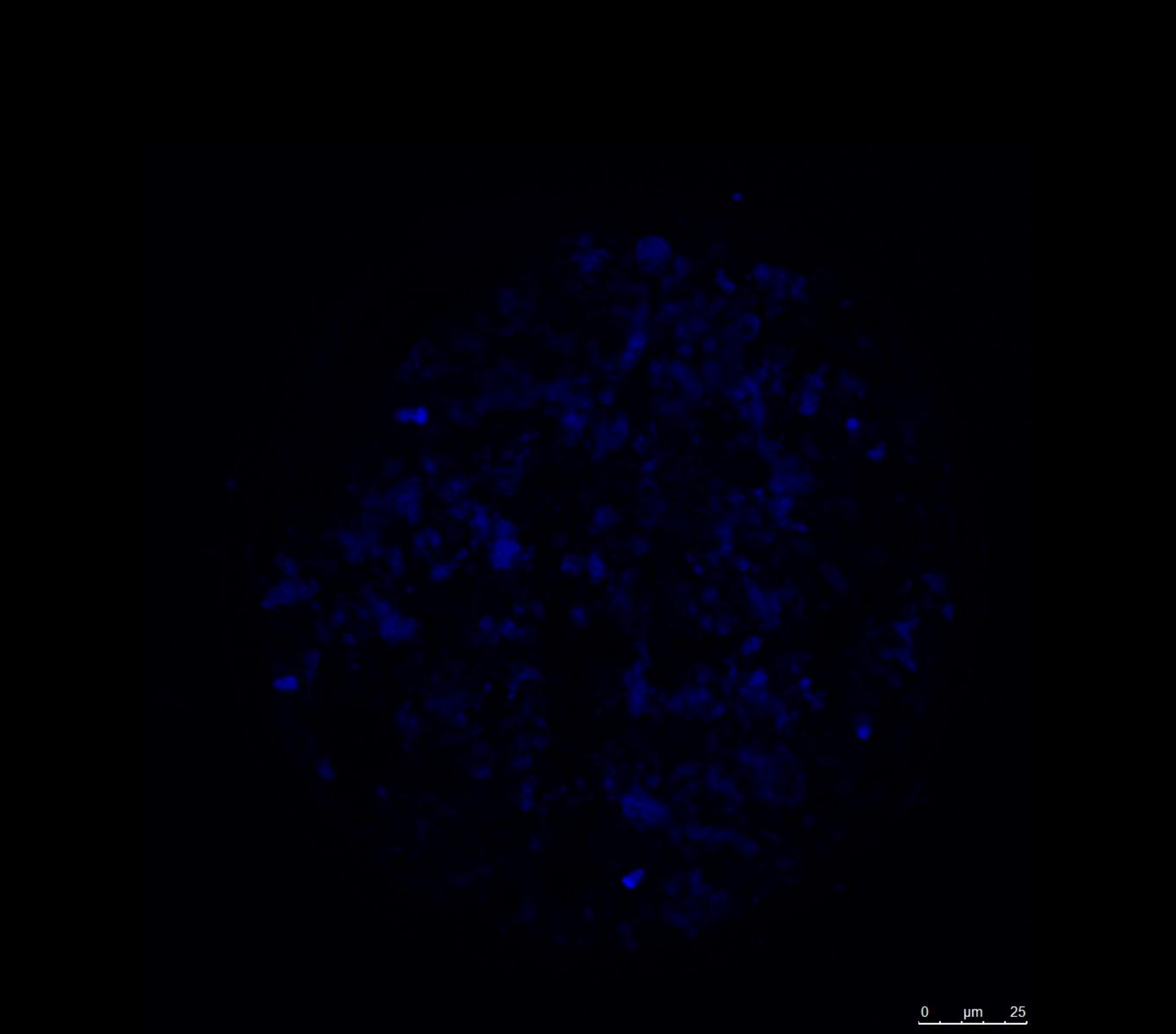

Fig. S4C

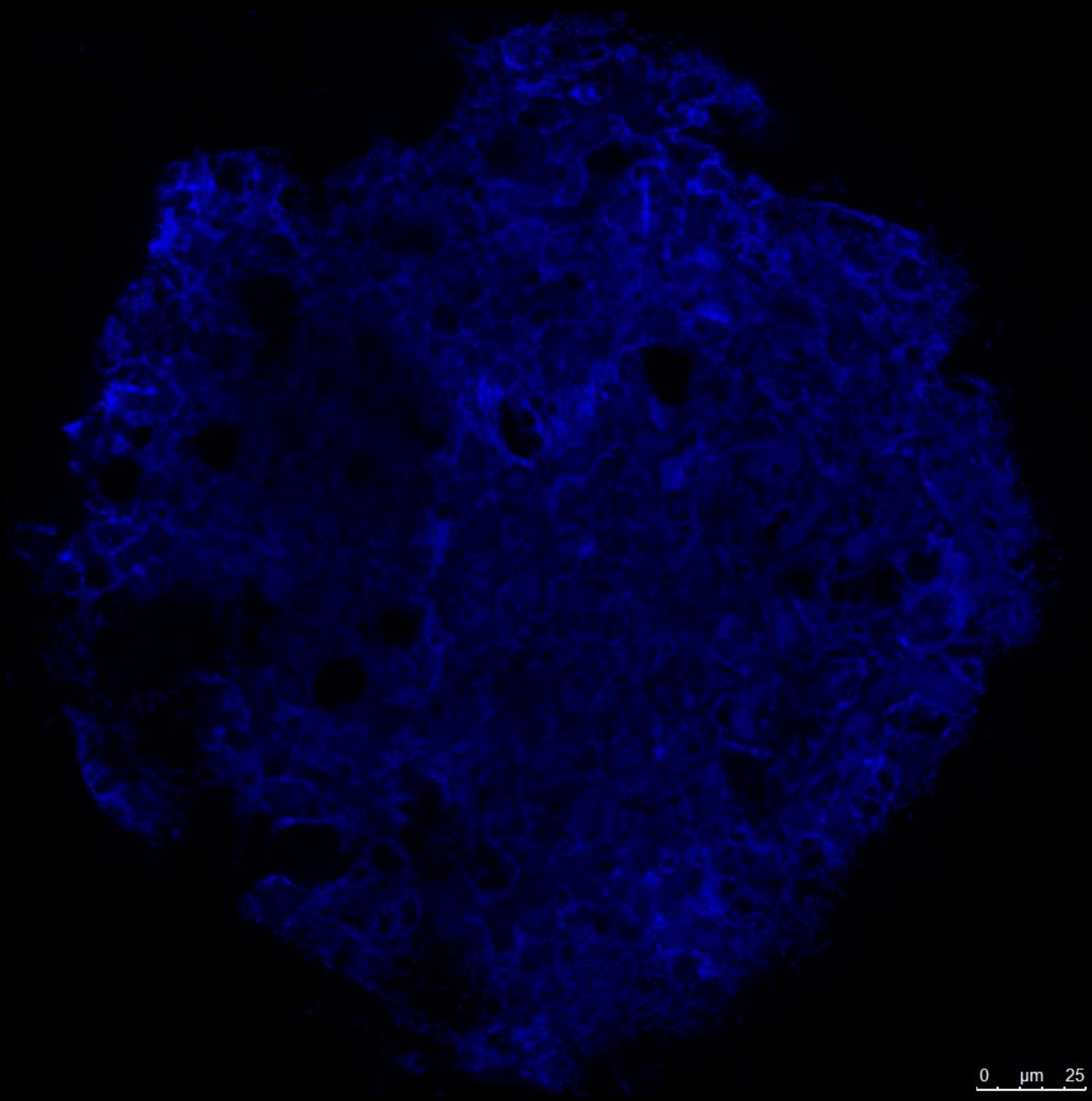

Fig. S4E

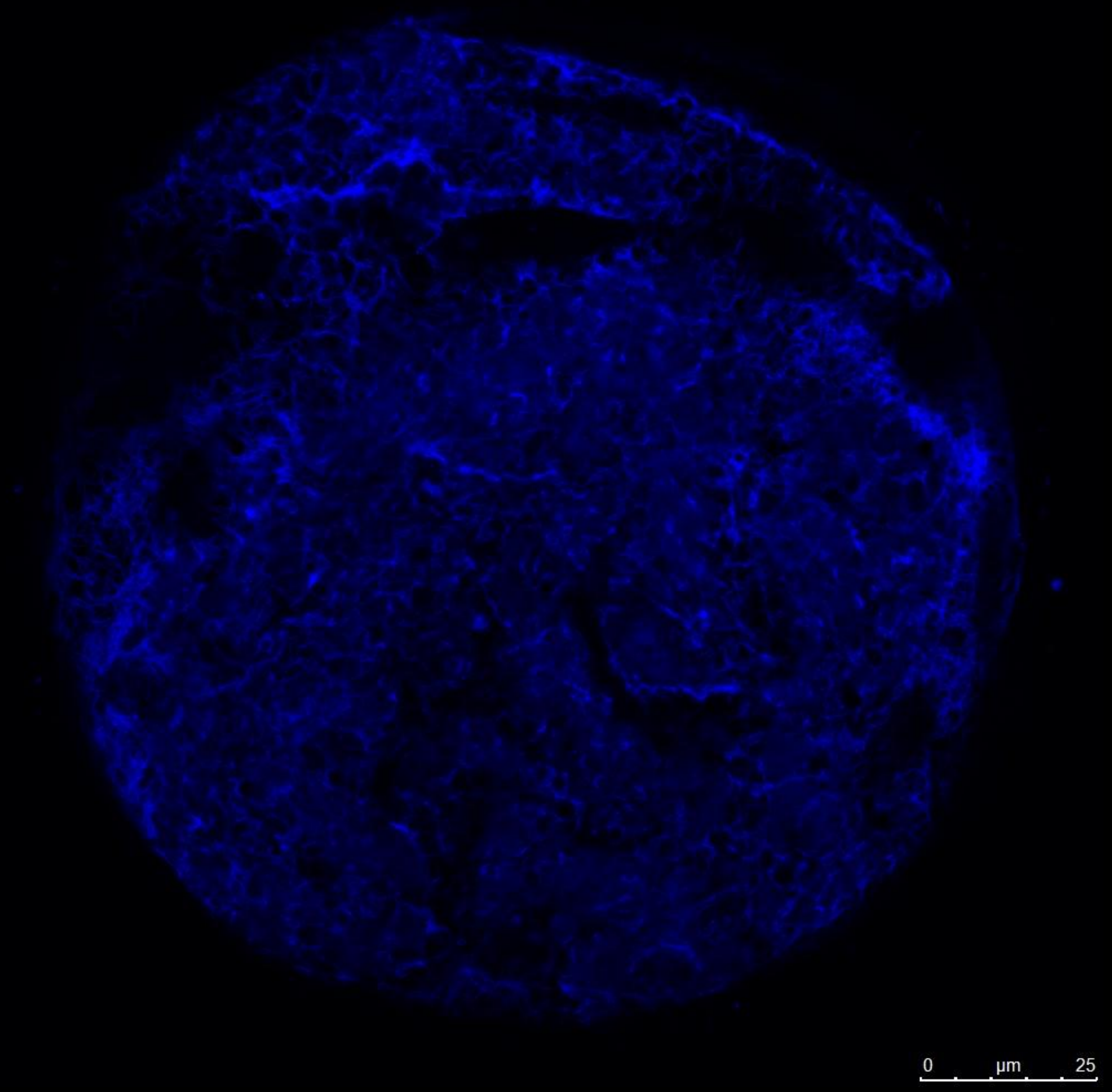

Fig. S4F

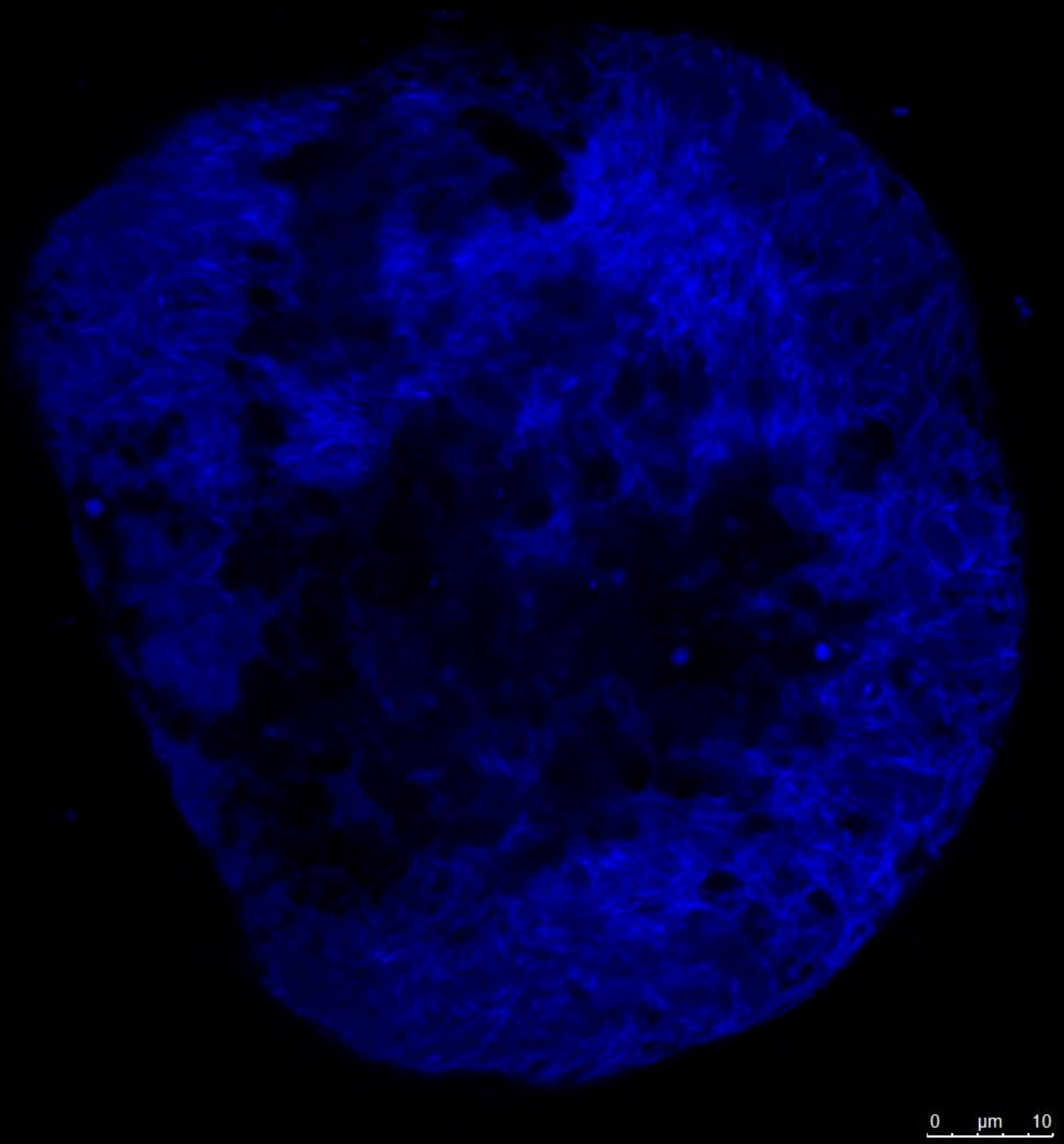

Fig. S4G

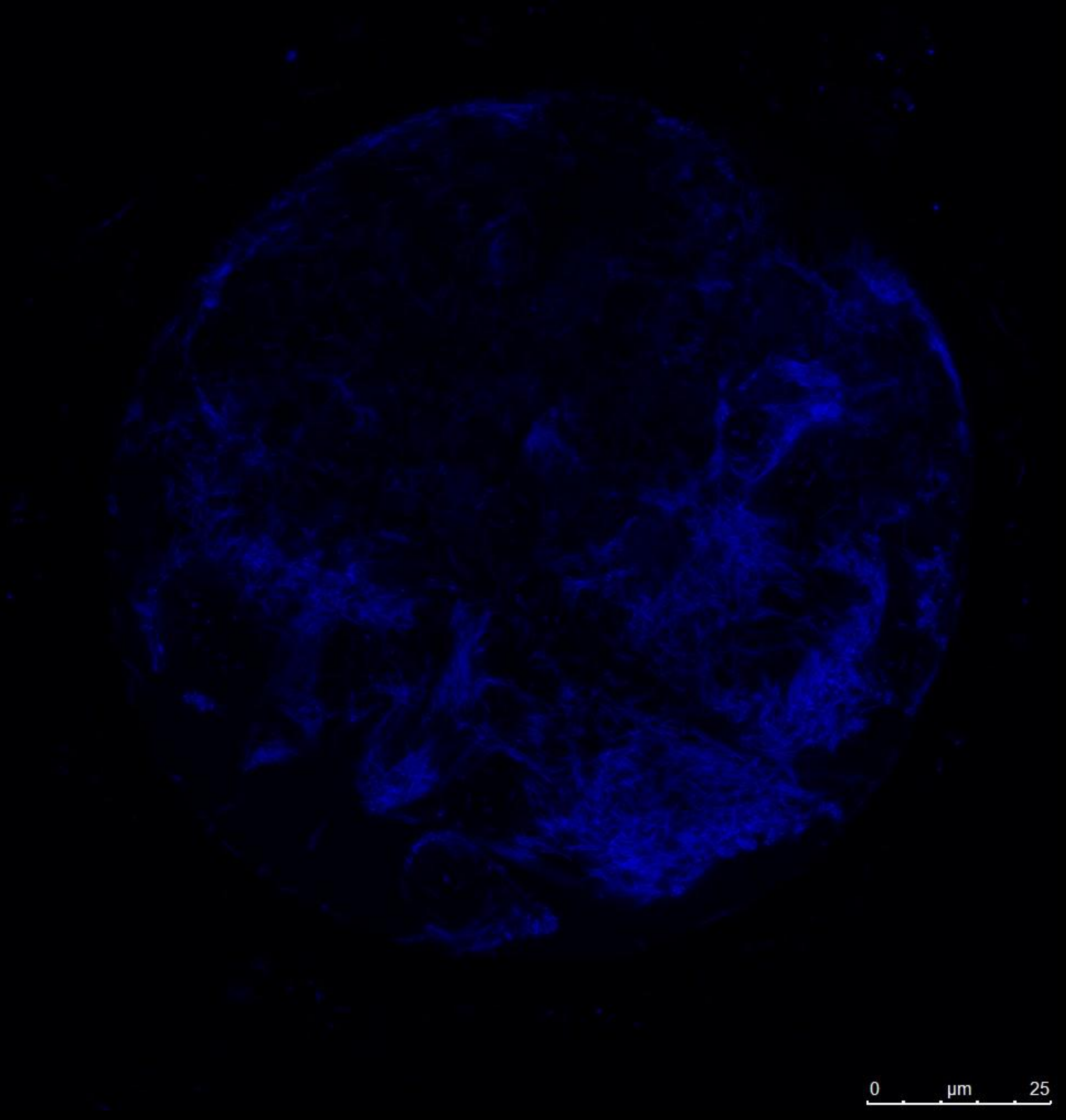

Fig. S4I

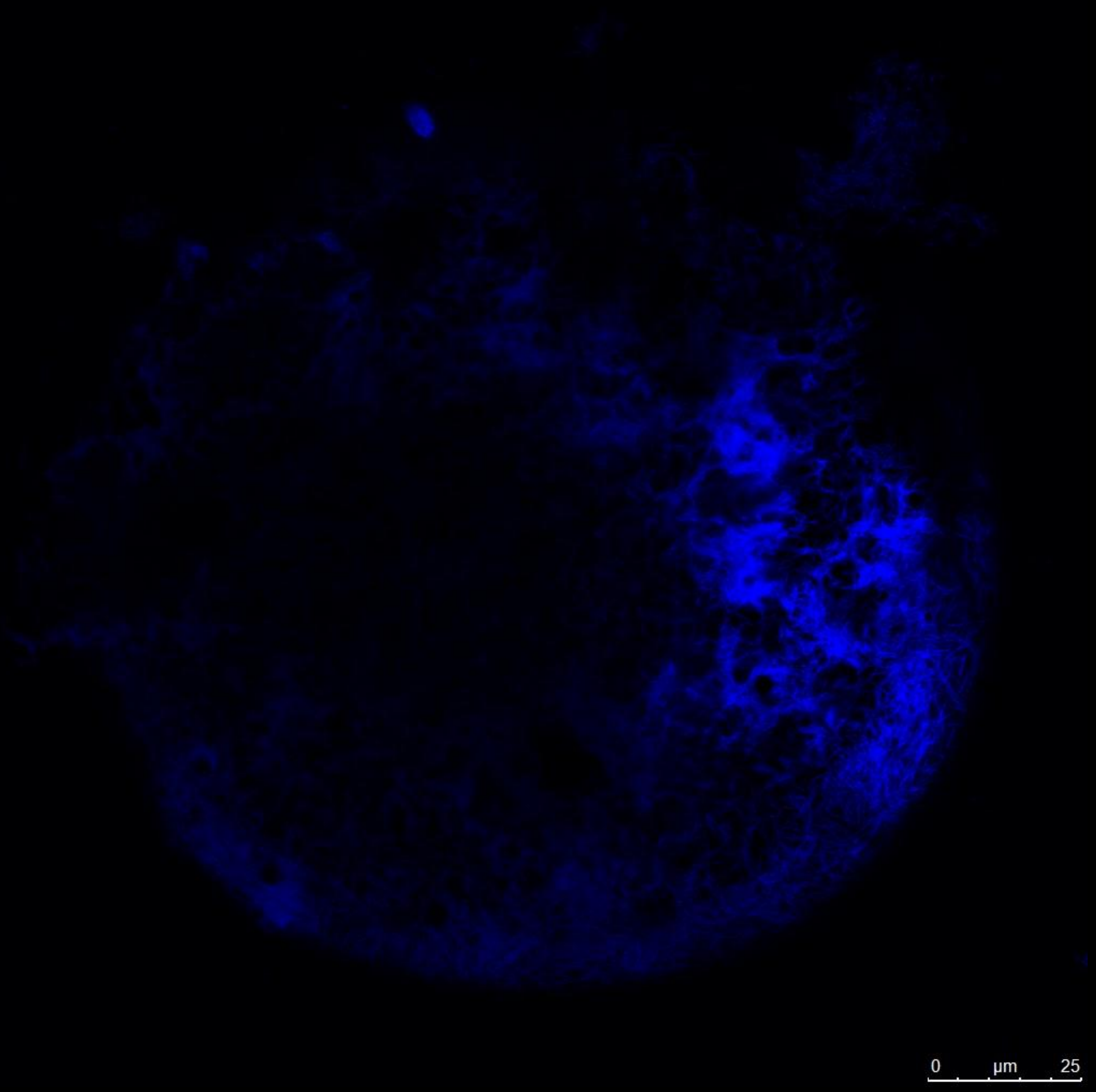

Fig. S4J

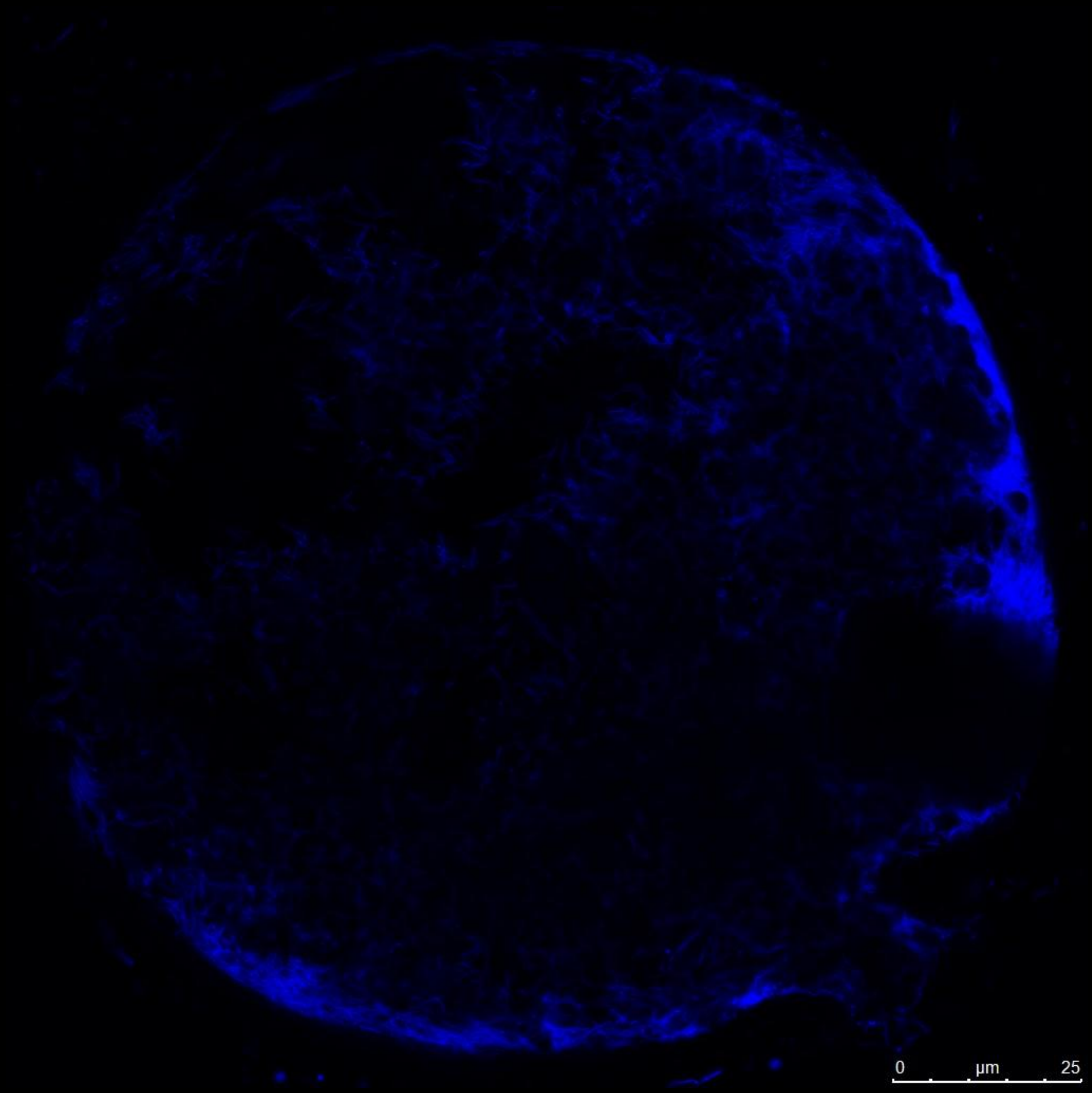

Fig. S4K

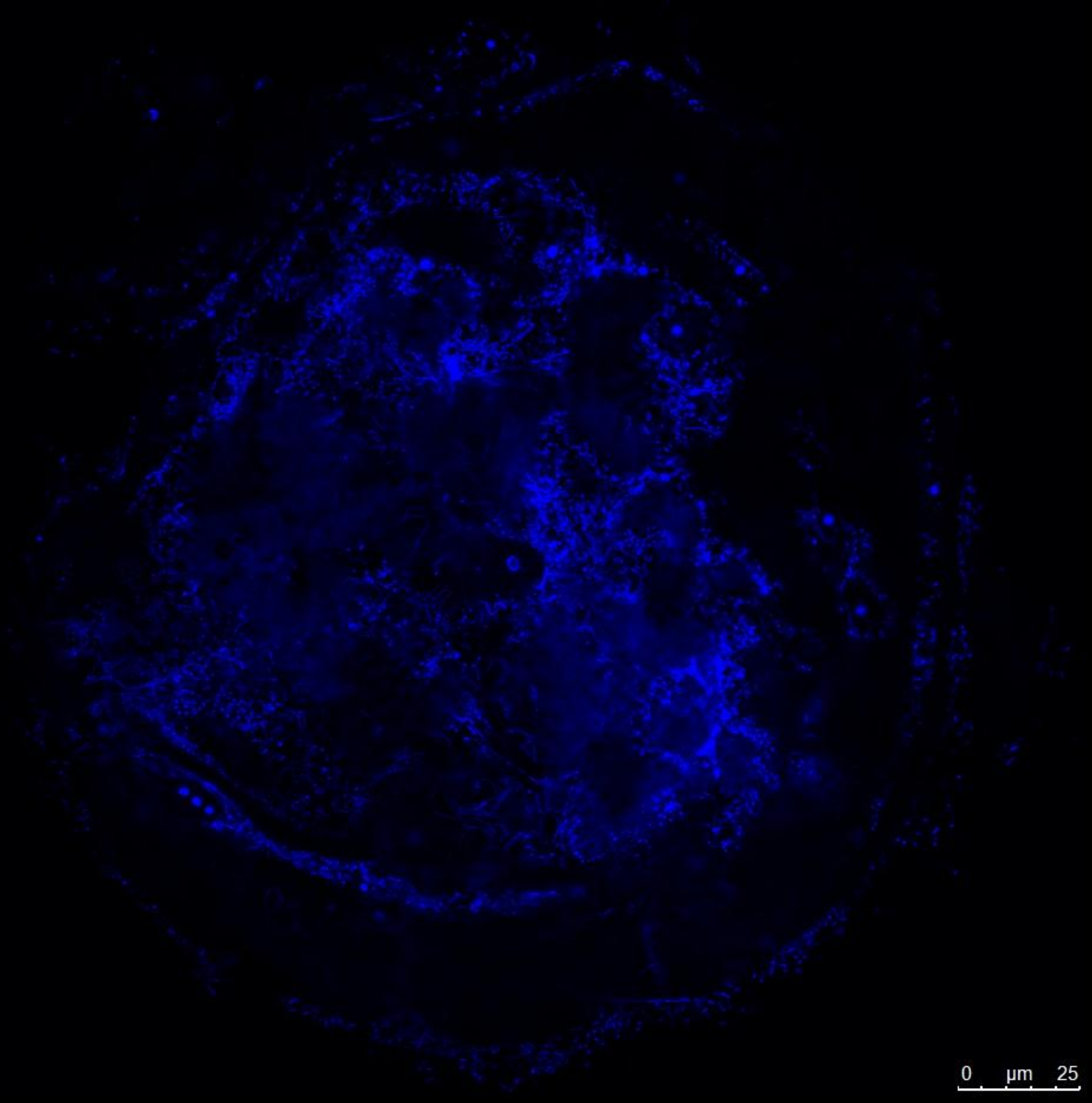

Fig. S4L

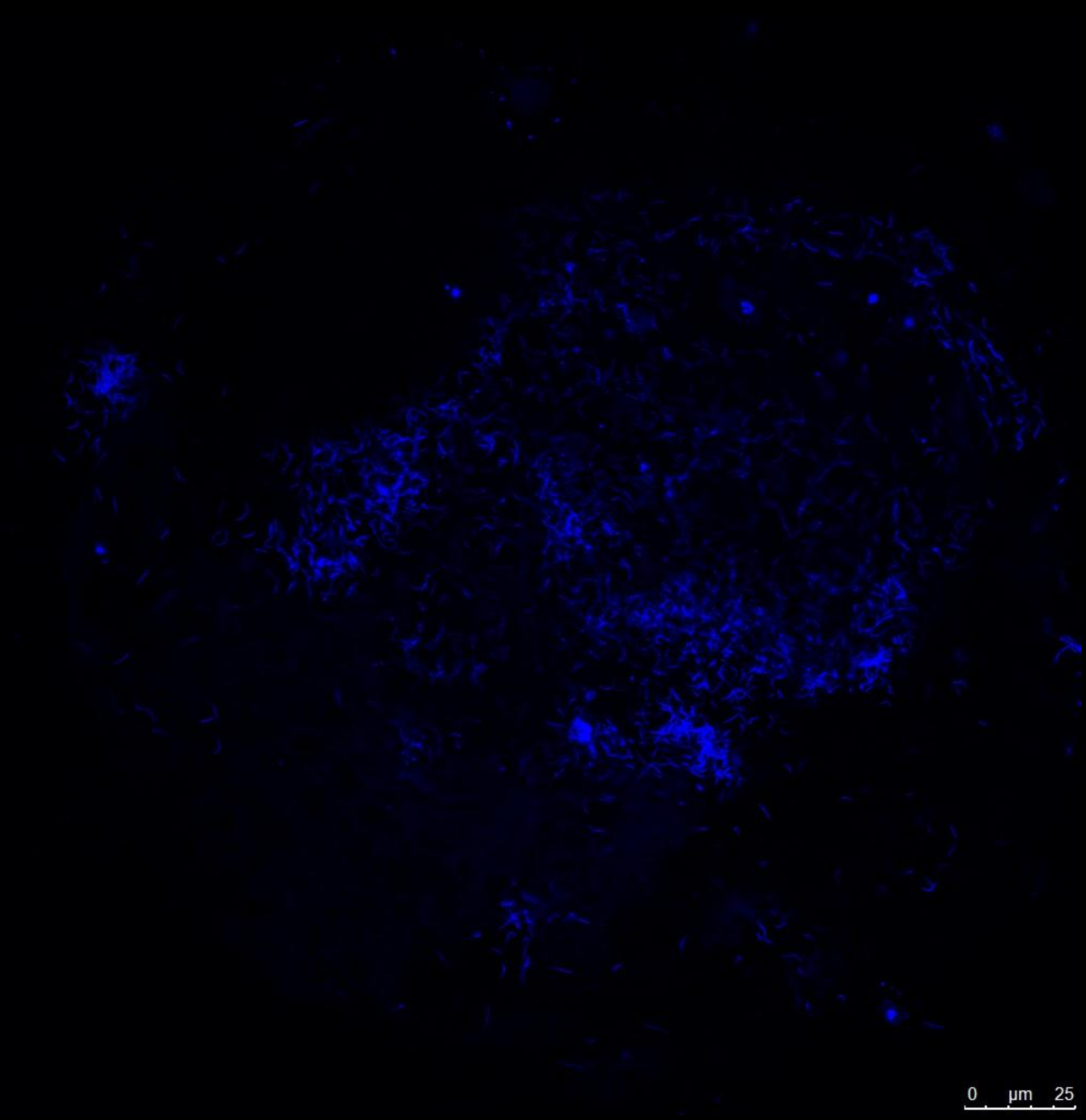

Fig. S4M

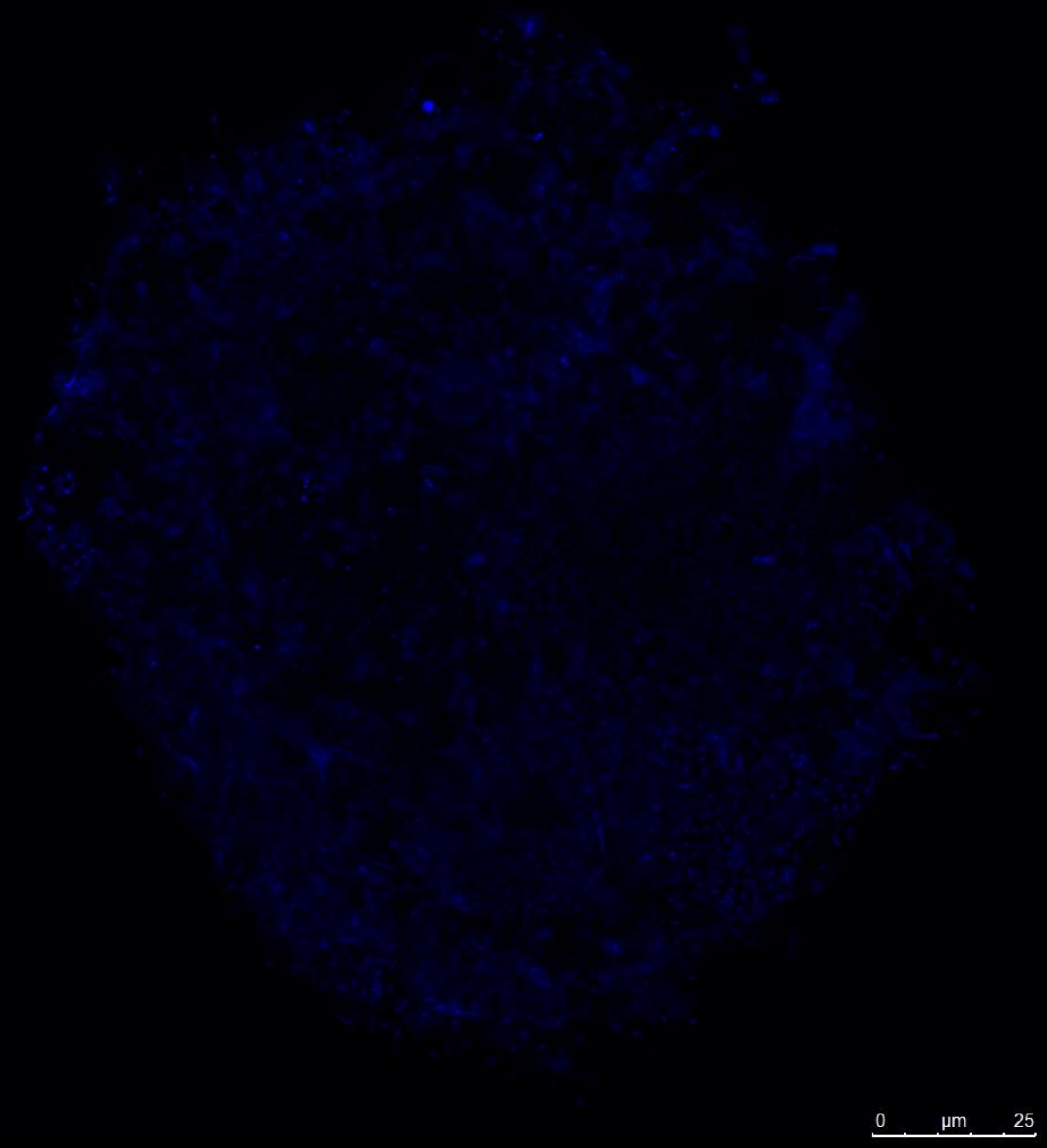

Fig. S4N

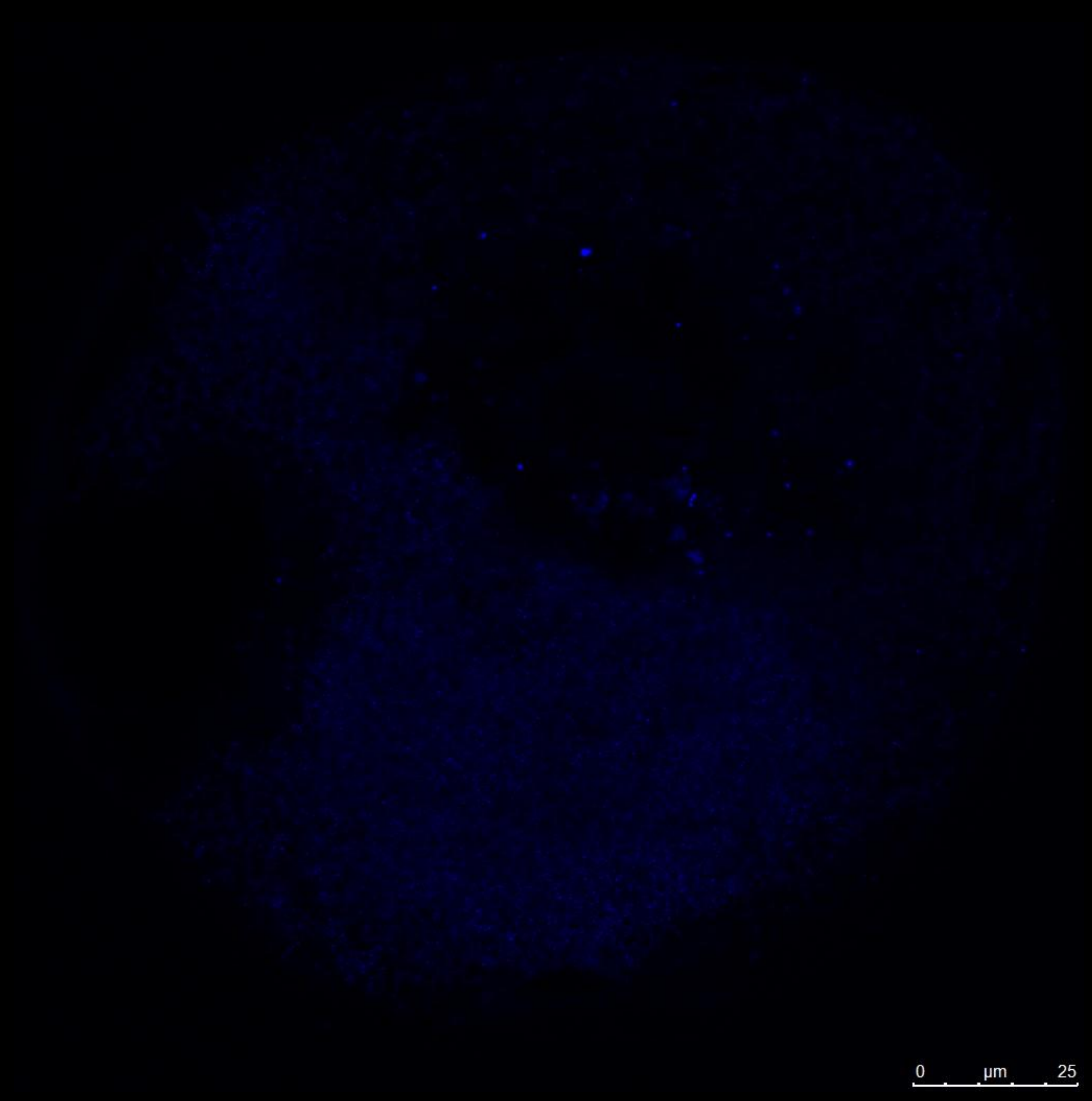

Fig. S4P
