# Supplementary material for: Foraminifera as a model of eukaryotic genome dynamism
Source: mBio. 2024 Feb 8;15(3):e03379-23. doi: 10.1128/mbio.03379-23 (PMC10936158; doi:10.1128/mbio.03379-23)
Supplement: Supplemental material — Supplemental tables, figures, and file notes. [file mbio.03379-23-s0005.pdf]

SUPPLEMENTARY FILES

File S1 Raw cell size measurements of isolated cells in 24-well plates.

Cell size measurements were taken using ImageJ software. Table is formatted such that there is one observation per well per day over the course of the experiment. Cells are marked with ‘ND’ if the cell had not yet emerged from its parent on that day, or if its offspring had already emerged by that day. The last measurement for each cell is a measurement of one of its offspring, if applicable.

File S2 Raw data from light microscopy observations of isolated cells in 24-well plates.

Note that wells 1.1 and 1.23 are excluded from Fig. 1 because of missing data.

File S3: Raw measurements of cell size, nuclear size, and fluorescence from Hoechst.

Each observation in the table represents a nucleus. Nuclei are annotated with one of the life stage categories, and nuclear architecture categories if applicable, in Table 5. Cell diameter was measured from brightfield images in ImageJ. Total nuclear fluorescence intensity, nuclear volume, and mean nuclear fluorescence intensity were measured in NIS-Elements.

File S4 Unedited fluorescent images of cells/nuclei shown in Figs. 2, 3, 4, S2, and S4.

Video S1: Z-stack of an *A. laticollaris* CSH multinucleated adult in meiosis with three types of nuclear architecture: V2, MB, and H nuclei (Table 1). Hoechst-stained material is shown in blue.

Video S2: Z-stack of an *A. laticollaris* CSH adult cell in *Zerfall* filled with Hoechst-stained chromatin structure fragments (Fig. 2; Fig. 4A, Fig. S4D). Hoechst-stained material is shown in blue.

Video S3: Z-stack of an *A. laticollaris* CSH adult cell in *Zerfall* filled with Hoechst-stained chromatin structure threads (Fig. 2; Fig. 4B, Fig. S4H). Hoechst-stained material is shown in blue.

Video S4: Z-stack of an *A. laticollaris* CSH adult cell in *Zerfall* filled with Hoechst-stained fragments, threads, and haploid genome complements (Fig. 2; Fig. 4A, Fig. S4D). Hoechst-stained material is shown in blue.

Table S1: Definitions of terms relating to the *A. laticollaris* CSH life cycle.

**Term**	**Definition**
Adult	Large (100-300 μm) darkly pigmented individuals; can be uninucleate (haploid) or multinucleate (diploid).
Autogamy	Fusion of gametes from the same parent cell; i.e. self-fertilization.
Emerging individuals	Juveniles as they leave the parental test; this process is referred to as schizogony in the foraminiferal literature
Endoreplication	Elevation of nuclear DNA content above the diploid level *via* whole-genome duplication or partial amplification.
Gametes	Amoeboid cell with single haploid nuclei that develop within the parental cell and eventually fuse with one another to form diploid zygotic nuclei. We infer that gametes are the end product of genome reorganization during *Zerfall.*
Juvenile	Small (< 100 μm) lightly-pigmented individuals that emerge from parental cell; can be uninucleate (haploid) or multinucleate (diploid).
Zerfall	Genome reorganization process in which we infer that an endoreplicated haploid nucleus degrades and releases genetic material into the cytoplasm where it is packaged into haploid gametes.
Zygotic nucleus	Diploid nucleus formed by fusion of gametes within one parent cell.

Table S2: Summary of *A. laticollaris* CSH morphological life stages. Minimal refers to a growth rate of less than 1 μm/day. Diameter, growth rate, and life stage duration are given as the mean ± one standard deviation.

Reproductive cell type emerged from	Life Stage	Cell Diameter Range (μm)	Average Growth Rate (μm/day)	Life Stage Duration (days)	Other characteristics
1	Juvenile	72 ± 14	5 ± 3	7 ± 2	Light pigmentation, simple pseudopodia
1	Adult	176 ± 49	9 ± 4	12± 3	Dark pigmentation, complex pseudopodia
1	Pre-reproductive	221 ± 33	minimal	2 ± 1	No pseudopodia, dark pigmentation
N/A	Type 1 reproductive	221 ± 33	minimal	1 ± 0.5	Gives rise to 13 ± 4 juveniles
2	Juvenile	58 ± 18	6 ± 2	10 ± 3	Light pigmentation, simple pseudopodia
2	Adult	165 ± 44	8 ± 5	17 ± 9	Dark pigmentation, complex pseudopodia
2	Pre-reproductive	205 ± 28	minimal	2 ± 1	No pseudopodia, dark pigmentation
N/A	Type 2 reproductive	Same as pre-reproductive	minimal	2 ± 1	Gives rise to 30 ± 23 juveniles

Table S3: Longitudinal observations of *A. laticollaris* CSH cells in culture flasks suggest synchrony**.**

Days are defined after the first day of flask observations. The initial life stage column describes the life stage of the majority of cells in a flask on the first day that observations were made of all flasks. N/A indicates that observations were not conducted for a given flask on a given day.

Flask	Initial Life Stage	Day 5	Day 6	Day 13	Day 21	Day 33
1	Large adults	Juveniles, adults, pre-reproductive	N/A	Small juveniles, medium adults	N/A	Large adults, pre-reproductive, juveniles within parental tests
2	Large adults	N/A	Juveniles, adults, juveniles within parent tests	Small juveniles, small to medium adults	N/A	Large adults, pre-reproductive, juveniles within parental tests
3	Large adults	Juveniles, adults, pre-reproductive	N/A	Medium adults, juveniles	Small-large adults, some larger juveniles	Large adults, pre-reproductive

Table S4: DNA content estimates for *A. laticollaris* CSH nuclei using different ratios of fluorescent units to base pairs based on analyses of standards (*S. cerevisiae*, *H. sapiens*, *A. cepa*). We report the results of each method as median (25th percentile–75th percentile). Method 1 estimates *Allogromia* DNA content using the median ratio of fluorescence per base pair (F/bp) in *S. cerevisiae* nuclei, which are closest in size and fluorescence to haploid gametes. Method 2 estimates *Allogromia* DNA content using the average of the median ratios of F/bp in *S. cerevisiae, H. sapiens,* and *A. cepa*. Abbreviations for nuclear architecture and ranges for estimates of DNA content (C) are in Table 1. Superscripts denote: 1) estimated DNA content in each haploid genome complement of late-*Zerfall* cells (Fig 4C); 2) estimated DNA content of gametic nuclei, set to 1.0 to allow comparisons; 3) total DNA in *Zerfall* cells, which lack nuclei.

**Inferred Life Stage**	**Nuclear Architecture**	**Estimated DNA content (C)**	**Method 1 Estimate (Gbp)**	**Method 2 Estimate (Gbp)**
Haploid complement[1]	H	1.3	0.044 (0.026–0.061)	0.33 (0.19–0.46)
Gamete[2]	H	1.0	0.034 (0.024–0.042)	0.25 (0.18–0.32)
Zygote	H	1.9	0.065 (0.038–0.10)	0.49 (0.29–0.77)
Multinuc juv. in parent test	HDP	7.2	0.24 (0.12–0.54)	1.8 (0.90–4.0)
Multinuc. Juvenile	H	4.7×10^2	16 (6.2–23)	1.2×10^2 ($0.46–1.7 \times 10^2$)
Multinuc. Adult	V1	4.9×10^2	17 (6.5–40.)	1.2×10^2 ($0.49–3.0 \times 10^2$)
	V2	85	2.9 (1.5–6.0)	21 (11–45)
	MB	55	1.9 (0.66–4.1)	14 (4.9–31)
	H	26	0.90 (0.47–2.7)	6.7 (3.5–20.)
	Other	69	2.3 (1.5–5.9)	17 (11–34)
Uninuc. juv in parent test	V	46	1.6 (0.80–3.4)	12 (6.0–25)
Uninuc. juvenile	V	5.4×10^3	1.8×10^2 ($1.6–2.8 \times 10^2$)	1.3×10^3 ($1.2–2.1 \times 10^3$)
Uninuc. Adult	VHB	11×10^3	3.6×10^2 ($2.1–4.1 \times 10^2$)	2.7×10^3 ($1.5–3.1 \times 10^3$)
	VHL	29×10^3	3.1×10^2 ($1.9–9.7 \times 10^2$)	2.3×10^3 ($1.4–7.2 \times 10^3$)
	Zerfall	10.0×10^3	3.4 ($1.3–4.2 \times 10^2$)	2.5×10^3 ($0.94–3.1 \times 10^3$)
Zerfall cell[3]	*NA*	9.0×10^3	3.1×10^2 ($1.6–4.8 \times 10^2$)	2.3×10^3 ($1.2–3.6 \times 10^3$)

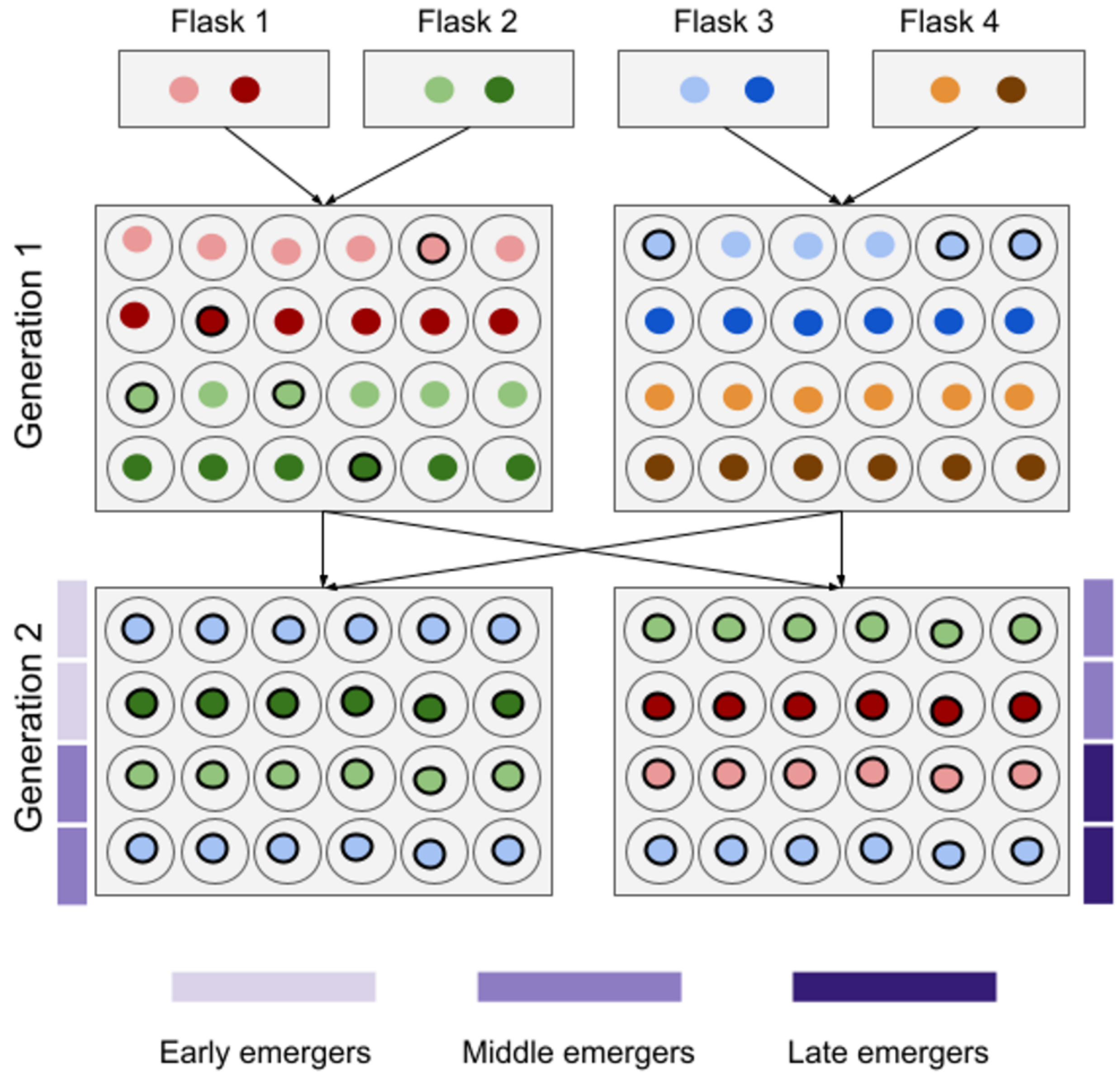

Figure S1 . Diagram of longitudinal cell observations by light microscopy.
Colored circles represent cells; each color represents juveniles from a single parental test. Arrows represent transfer of offspring. Top: eight reproductive cells from four culture flasks produced the 48 offspring that were observed throughout their life cycles (Generation 1). Eight cells from Generation 1 (two early reproducers, four middle reproducers, and two late reproducers) produced the 48 offspring observed in Generation 2. Generation 2 cells and their Generation 1 parents are indicated by black outlines. The emergence time of Generation 2 cells (whether they derive from early, middle, or late reproducers) is denoted by purple shaded bars.

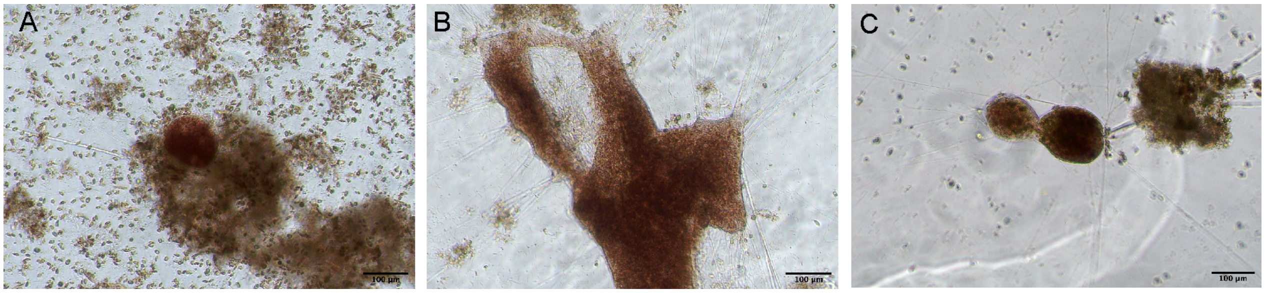

Figure S2: *A. laticollaris* CSH cells that exhibit alternative life cycle pathways.

A) An individual that remained in the adult stage for two months without undergoing Scale bars on all images are 100 µm. A) An individual (center, reddish-brown cell surrounded by brown algae) that remained in the adult stage for two months without undergoing reproduction or transitioning to any other life cycle stage. The image was taken two months after the cell emerged from its parental cell (File S2). B) Budding cell; this single offspring was the sole 'emerger' from its parental cell. The budding cell is the reddish-brown structure in the center with clear pseudopods protruding from all sides. Each prong of cytoplasm will detach to form another adult cell. C) A second cell in the process of budding. The more darkly-pigmented cell in the center is the parent cell in the process of budding, while the smaller, lighter cell to its left is its developing offspring. This cell appeared like a normal adult prior to budding.

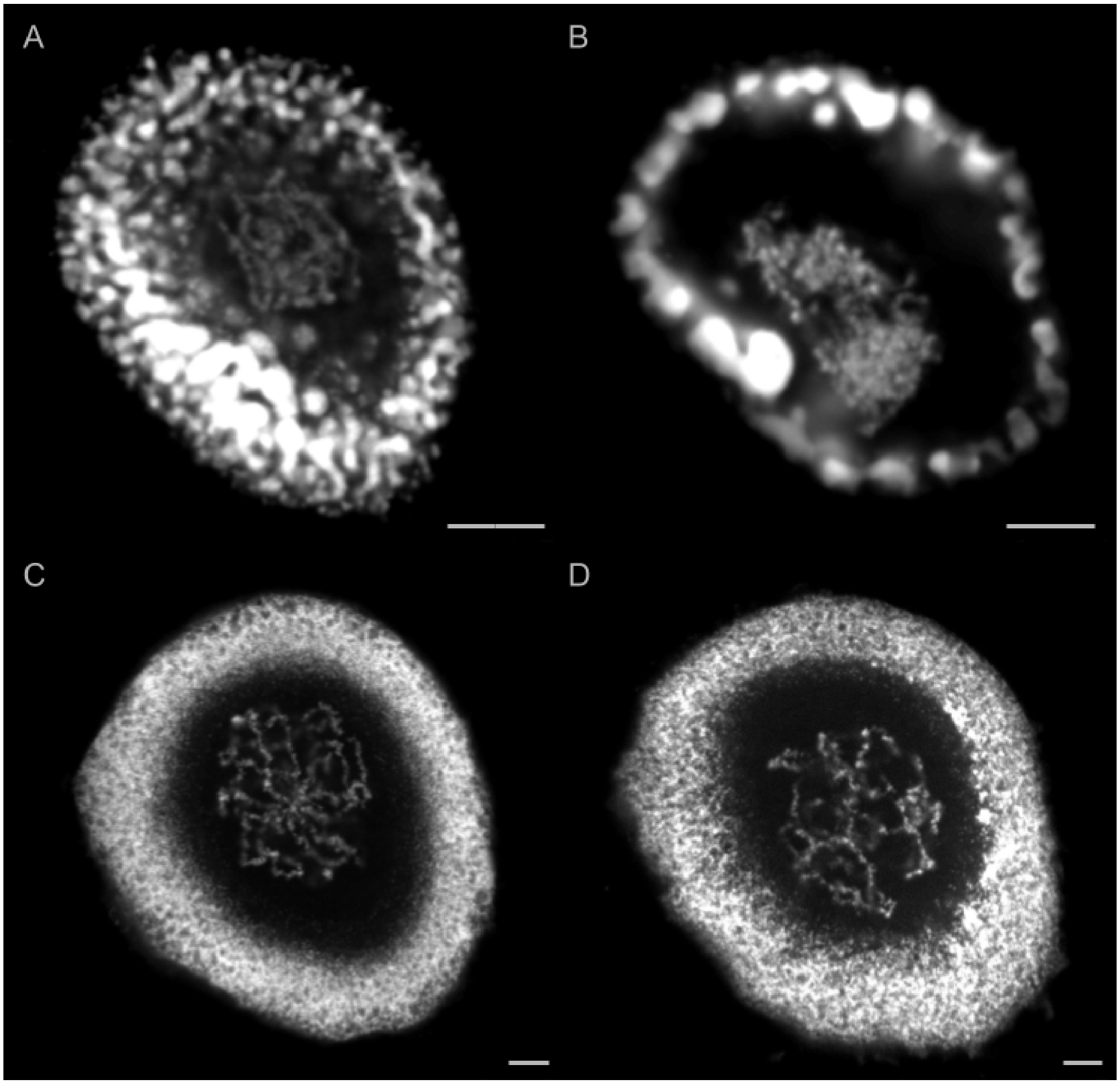

Figure S3: Representative fluorescent images of nuclei with either beaded or latticed chromatin structure rings at the nuclear periphery.

Hoechst-stained *Allogromia* nuclei, shown in grayscale with brightness and contrast enhanced. All images contain nuclei from uninucleate adult cells. All scale bars are 5 μm. A-B) Representative nuclei with beaded chromatin rings and central chromosomes. The beaded chromatin ring is comprised of ball-like structures surrounding a central DNA-poor region where filament-like chromosomes are visible. C-D) Representative nuclei with latticed chromatin rings and central chromosomes. The latticed chromatin is a thicker ring with a mesh-like structure that also surrounds a DNA-poor region with chromosomes visible. We speculate that latticed chromatin structure rings are a precursor to *Zerfall*, based on their structural similarity to the chromatin structure spread throughout *Zerfall* nuclei (Fig. S4A).

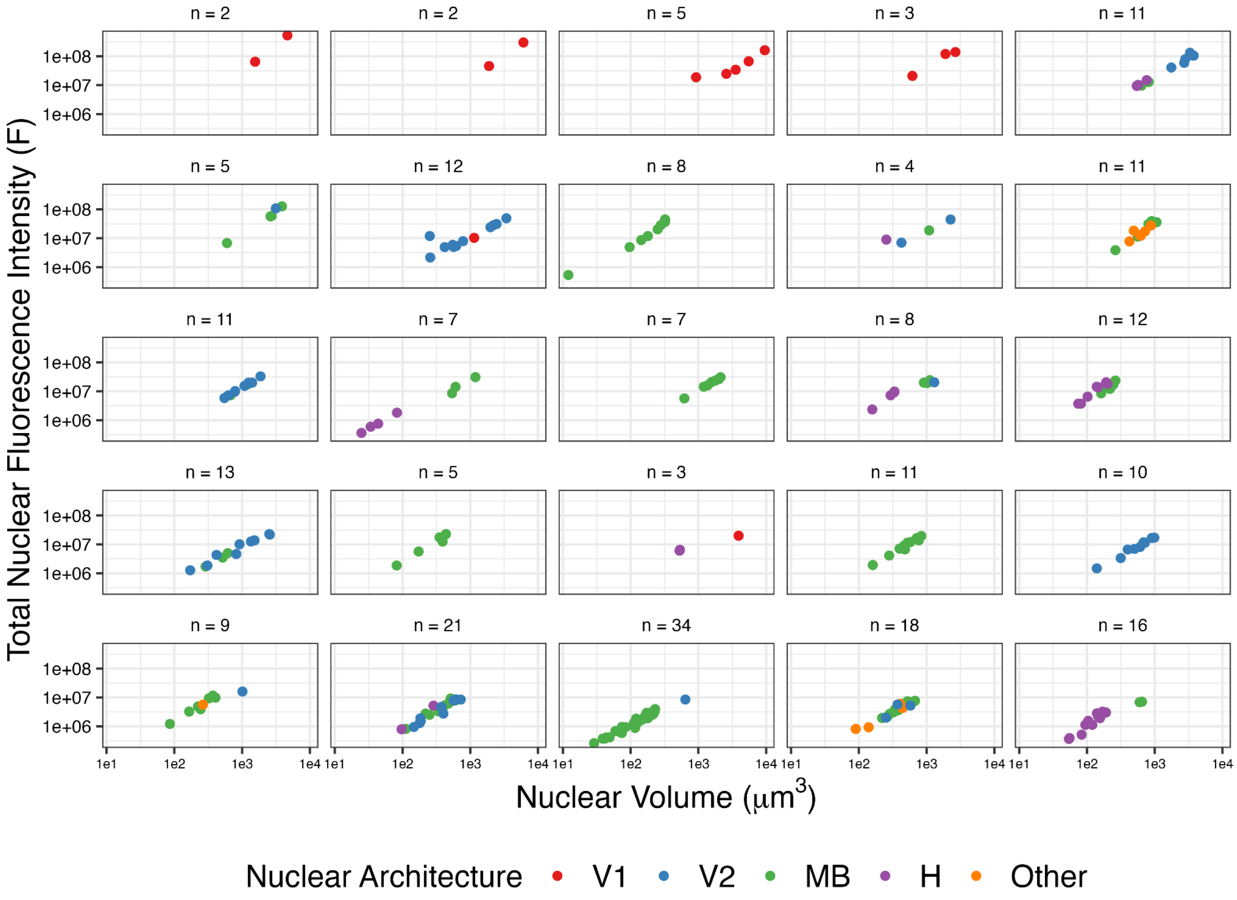

Figure S4: Variation in nuclear size and fluorescence intensity corresponds to variation in nuclear architecture within multinucleate adult cells, and suggests temporal stages from vegetative to post-meiotic nuclei.

Scatterplots compare total nuclear fluorescence intensity on the y-axis to nuclear volume on the x-axis for 25 multinucleate adult cells. Each point represents a nucleus, colored according to its architecture. V1 = Vegetative pre-division, V2 = Vegetative post-division, MB = Meiotic bouquet, H = Homogeneous (Table 1). Each facet shows all of the nuclei measured within a single cell. Facet labels denote the number of nuclei measured in each cell. Facets are arranged in L-R descending order from the top left corner by the maximum fluorescence intensity within a single nucleus in the cell. We hypothesize the homogenous nuclei are the products of meiosis, which occurs within the context of chromatin structure material that is maintained on the outer edge of nucleus through divisions

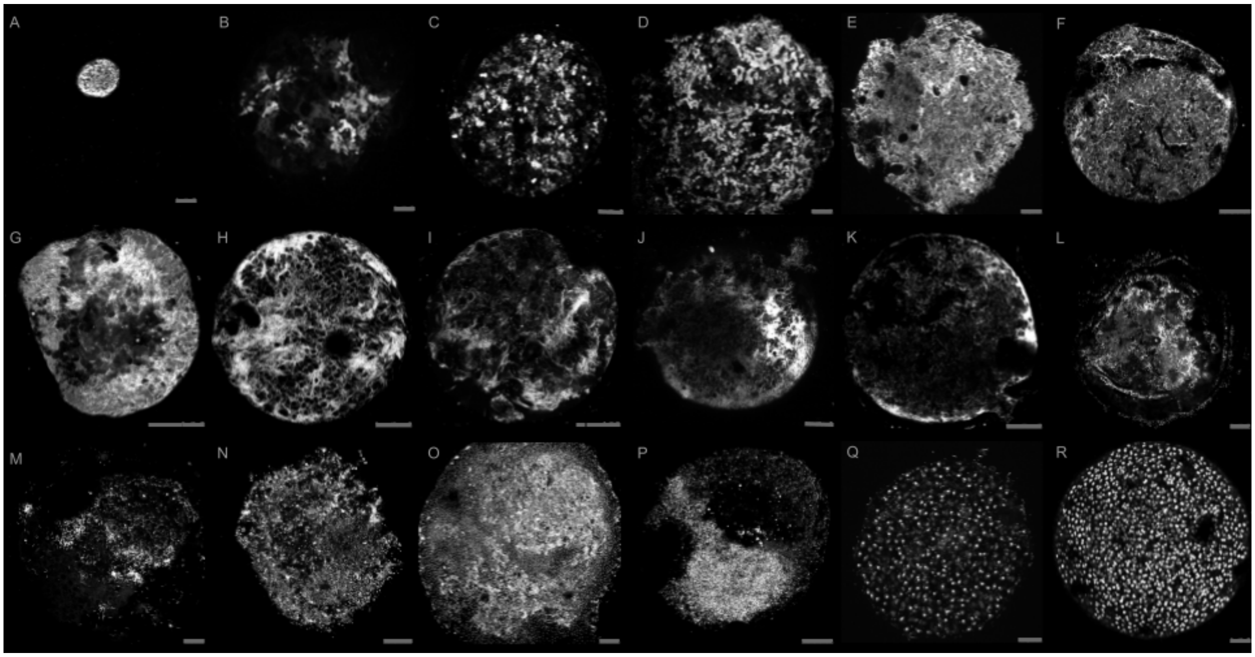

Figure S5: Inferred sequence of genome reorganization during *Zerfall* that leads to gametogenesis in *A. laticollaris* CSH.

Each image shows an *A. laticollaris* CSH cell stained with Hoechst 33342, with images converted to grayscale and the brightness and contrast enhanced; raw fluorescence data can be found in File S3 and original images in File S4. All scale bars are 25 μm. A) *Zerfall* begins when the internal nuclear architecture (i.e. condensed chromatin surrounding DNA poor center) within a uninucleate adult cell breaks down, spreading chromatin material throughout the nucleus). B-D) The nuclear envelope breaks down, releasing globular chromatin material into the cytoplasm. E-F) Globular chromatin structures begin to transform to give rise to threadlike material. G-K) Threadlike material fills the cytoplasm. L-N) Haploid genome complements arise from threadlike material in the form of small, spherical, Hoechst-positive structures interspersed among chromatin structure fragments and threads. O-P) Thousands of haploid genome complements fill the cytoplasm. Q) As haploid genome complements grow in size into haploid nuclei, parental cytoplasm divides to form amoeboid gametes. R) Haploid gametes fuse within the parent cell to form diploid zygotic nuclei; gametic cell membranes are no longer visible, consistent with the next stage of generating multinucleate offspring.
